# Supplementary material for: MicroRNA-33 inhibition ameliorates muscular dystrophy by enhancing skeletal muscle regeneration
Source: EMBO Mol Med. 2025 Jul 23;17(8):1902–25. doi: 10.1038/s44321-025-00273-9 (PMC12340133; doi:10.1038/s44321-025-00273-9)
Supplement: Supplementary file 1 — Appendix [file 44321_2025_273_MOESM1_ESM.pdf]

MicroRNA-33 inhibition ameliorates muscular dystrophy by enhancing skeletal muscle regeneration

Appendix:

Page 1. Appendix Figure S1. Relative abundance of miR-33a expression in skeletal muscle.
Pages 2-3. Appendix Figure S2. miR-33a deficiency accelerates skeletal muscle regeneration after CTX injection
Page 4. Appendix Figure S3. Cdk6 is a target of miR-33.
Page 5. Appendix Figure S4. Generation of miR-33a and *mdx* double-deficient (KO/*mdx*) mice.
Pages 6-7. Appendix Figure S5. miR-33a deficiency increases myoblast expansion and differentiation.
Pages 8-9. Appendix Figure S6. Generation of mIR-33b knock-in and *mdx* mice (miR-33b-KI/*mdx* mice).
Pages 10-11. Appendix Figure S7. AAV9-mediated rescue experiments for *Cdk6* and *Abca1*.
Pages 12-13. Appendix Figure S8. *Fst* is a target gene of miR-33.
Pages 14-15. Appendix Figure S9. Local delivery of anti-miR33a ameliorates dystrophic phenotypes in *mdx* mice.
Pages 16-17. Appendix Figure S10. Effect of systemic administration of anti-miR-33b at a dose of 20mg/kg bw.
Pages 18-19. Appendix Figure S11. Effect of systemic administration of anti-miR33b at a dose of 10mg/kg bw.
Page 20. Appendix Figure S12. Analysis of myotubes differentiated from DMD patient-derived iPS cells treated with anti-miR-33b.
Page 21. Appendix Table S1. Serum data of mice administered AMOs ata a dose of 10mg/kg bw.
Page 22. Appendix Table S2. List of upregulated genes by anti-miR33b AMO, which are predicted as miR-33 target genes.
Page 23. Appendix Table S3. Details of primer pairs.

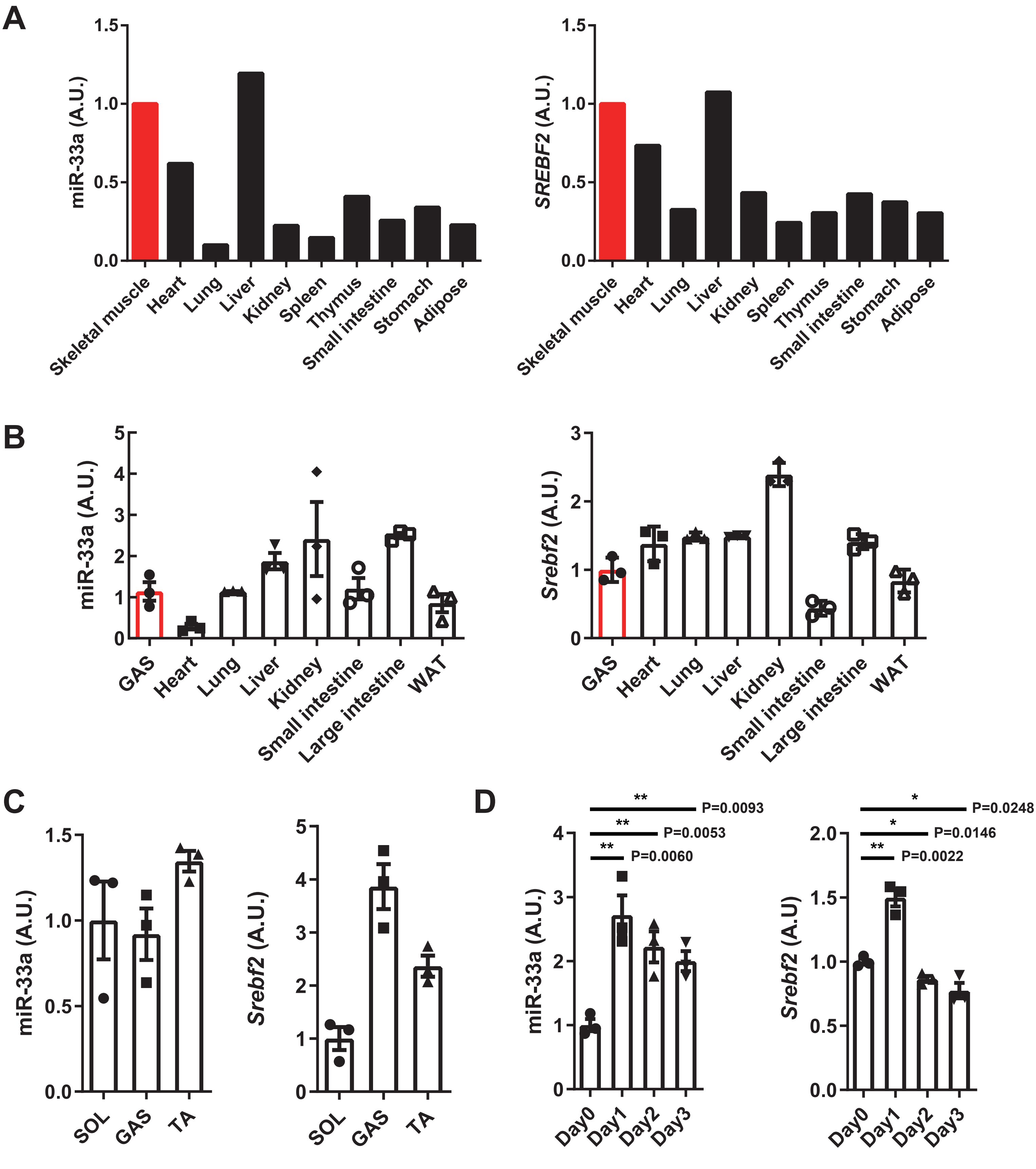

Appendix Figure S1. Relative abundance of miR-33a expression in skeletal muscle. (A) Expression of miR-33a and *SREBF2* in human organs (n = 1/group). (B) Expression of miR-33a and *Srebf2* in organs of WT mice (n = 3/group). (C) Expression of miR -33a and *Srebf2* in the soleus (SOL), gastrocnemius (GAS), and TA muscles of WT mice (n = 3/group). (D) Expression of miR-33a and *Srebf2* in C2C12 cells during myogenic differentiation (n = 3/group). One-way ANOVA withTukey post-hoc test. Data are presented as the mean ± SEM. *p < 0.05, **p < 0.01.

Appendix Figure S1

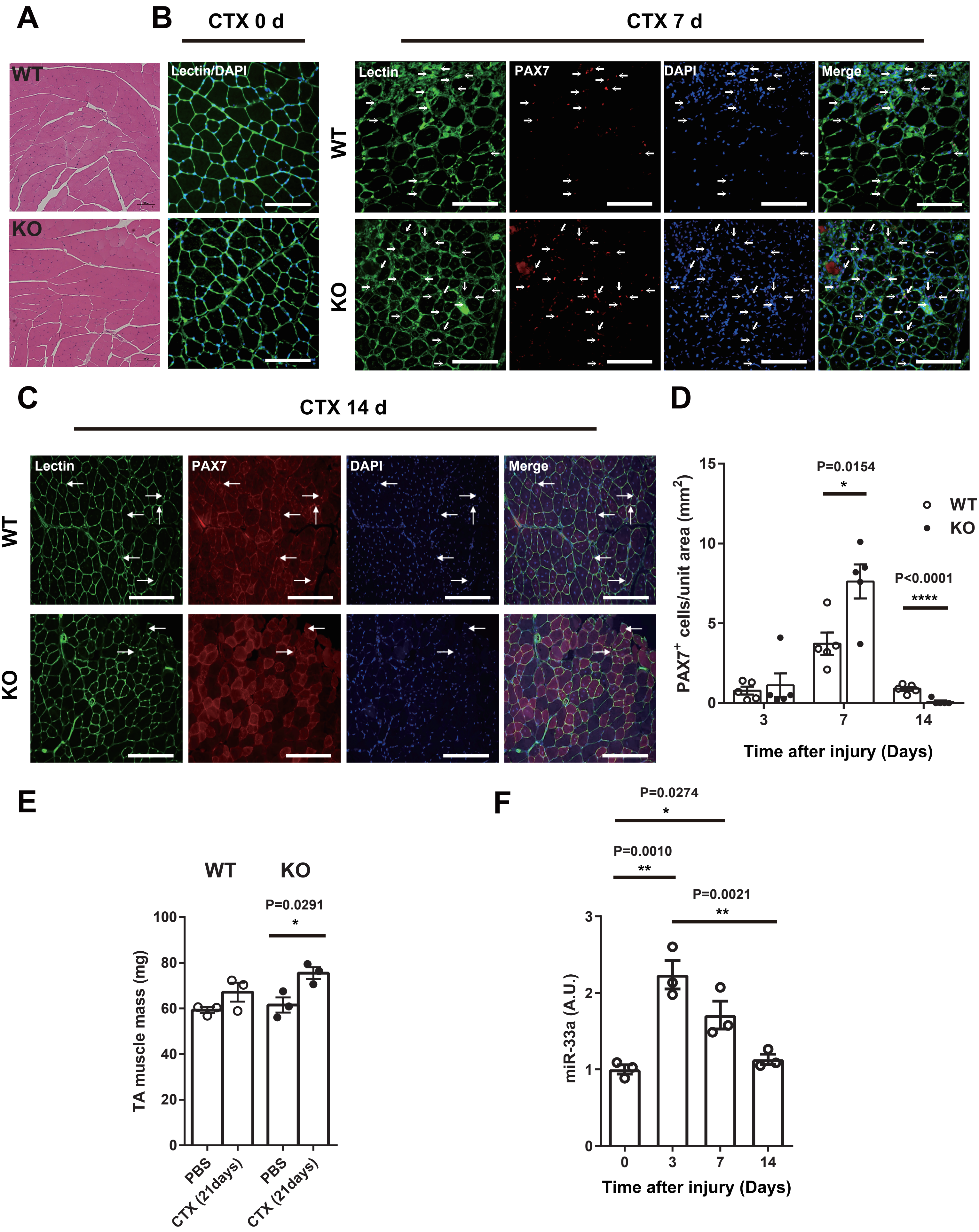

Appendix Figure S2

Appendix Figure S2. miR-33a deficiency accelerates skeletal muscle regeneration after CTX injection. (A) Representative images of HE staining of TA muscle of WT and miR-33a deficient (KO) mice under a steady state. Scale bar: 100 µm. (B) Representative fluorescent images of lectin and DAPI staining of TA muscle in WT and KO mice under a steady state and 7 days after CTX injection. Arrows indicate $Pax7^{+}$ cells. Scale bar: 100 µm. (C) Representative fluorescent images of lectin, Pax7, and DAPI staining of TA muscle in WT and KO mice 14 days after CTX injection. Arrows indicate $Pax7^{+}$ cells. Scale bar: 100 µm. (D) Number of $Pax7^{+}$ cells in the TA muscle of WT and KO mice during the regenerative response to CTX injection (n = 5/group). Unpaired *t*-test. (E) Weight of TA muscle of WT and KO mice 21 days after PBS or CTX injection (n = 3/group). Unpaired *t*-test. (F) miR-33a expression in the TA muscle of WT mice during the regenerative response to CTX injection (n = 3/group). One-way ANOVA with Tukey post-hoc test. Data are presented as the mean ± SEM. *p < 0.05, **p < 0.01, ****p < 0.0001.

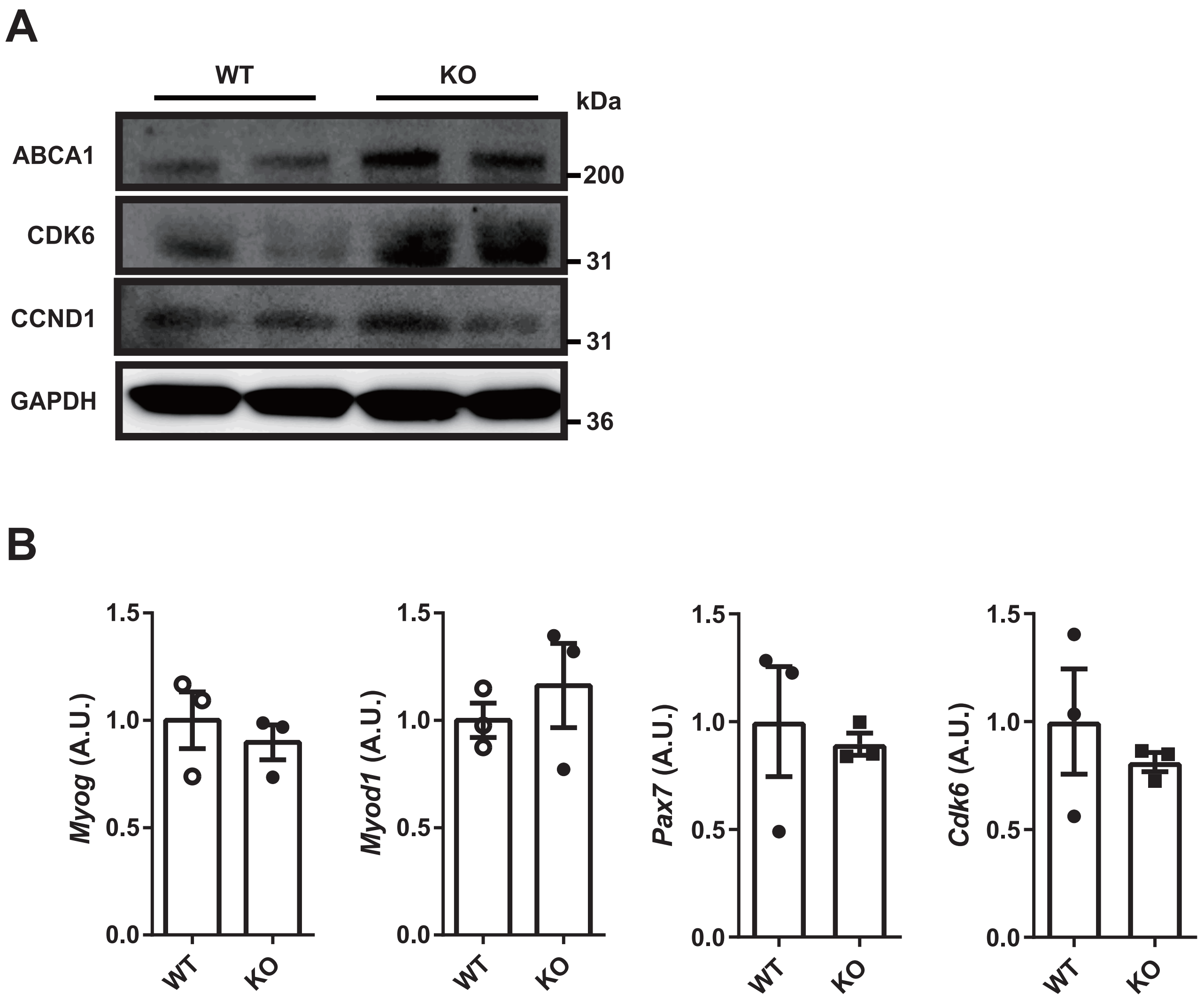

Appendix Figure S3. ***Cdk6*** **is a target of miR -33.** (A) Western blotting for ABCA1, CDK6, CCND1, and GAPDH proteins in the TA muscle of WT and KO mice. (B) Expression of *Myog*, *Myod1*, *Pax7*, and *Cdk6* in the TA muscle of WT and KO mice(n = 3/group). Data are presented as the mean ± SEM.

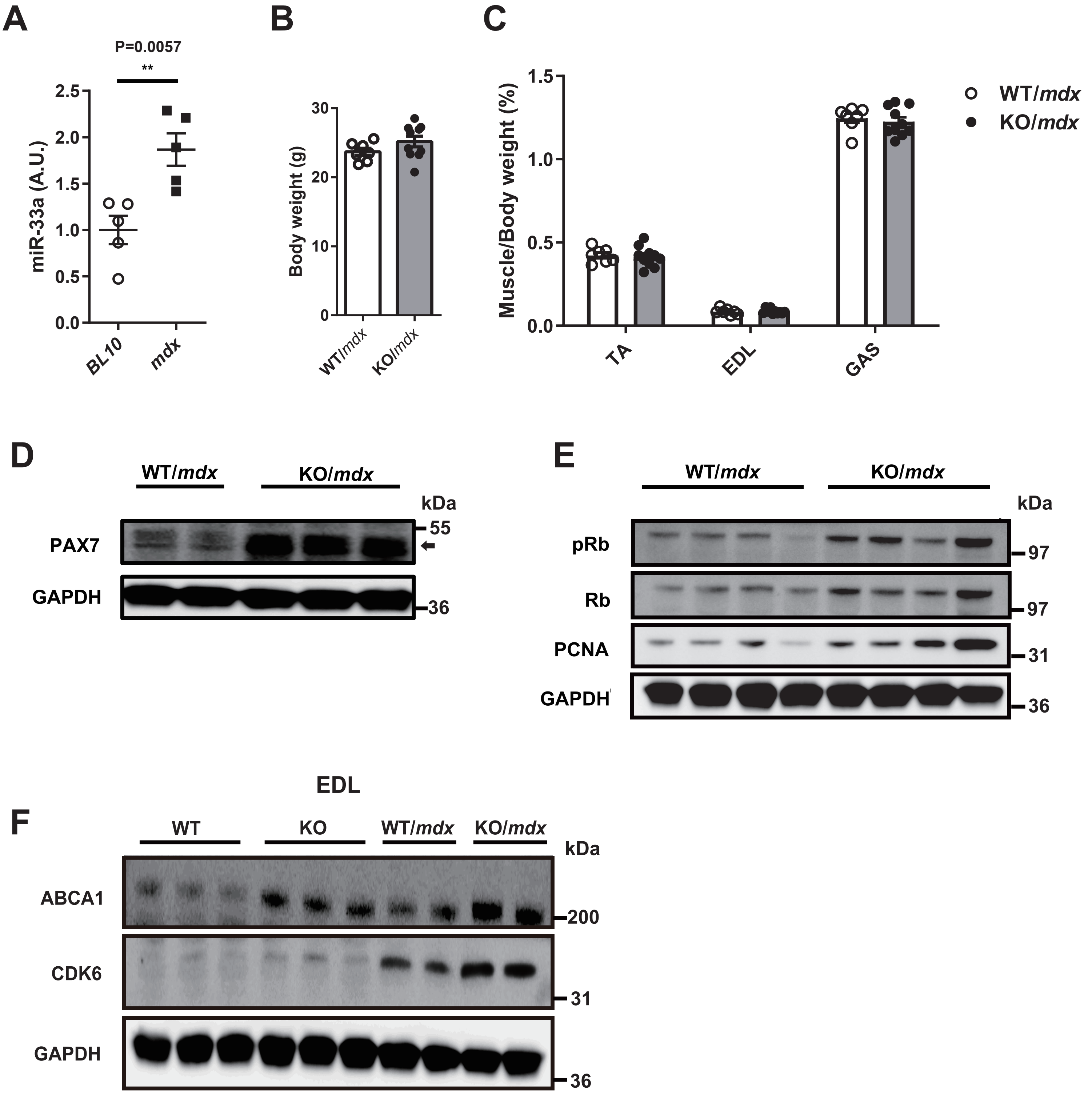

Appendix Figure S4 . Generation of miR-33a and *mdx* double-deficient (KO/*mdx*) mice. (A) miR-33a expression in TA muscle of BL10 and *mdx* mice (n = 5/group). Unpaired *t*-test. (B) Body weight of 8-week-old WT/*mdx* (n = 7) and KO/*mdx* (n = 10) mice. (C) Weight of TA, EDL, and GAS muscles in 8-week-old WT/*mdx* (n = 7) and KO/*mdx* (n = 10) mice. (D) Western blotting for PAX7 and GAPDH in TA muscle of WT/*mdx* and KO/*mdx* mice. (E) Western blotting for pRb, Rb, PCNA, and GAPDH in TA muscle of WT/*mdx* and KO/*mdx* mice. (F) Western blotting for ABCA1, CDK6, and GAPDH in EDL muscle of WT, KO, WT/*mdx*, and KO/*mdx* mice. Data are presented as the mean ± SEM. **p < 0.01.

Appendix Figure S4

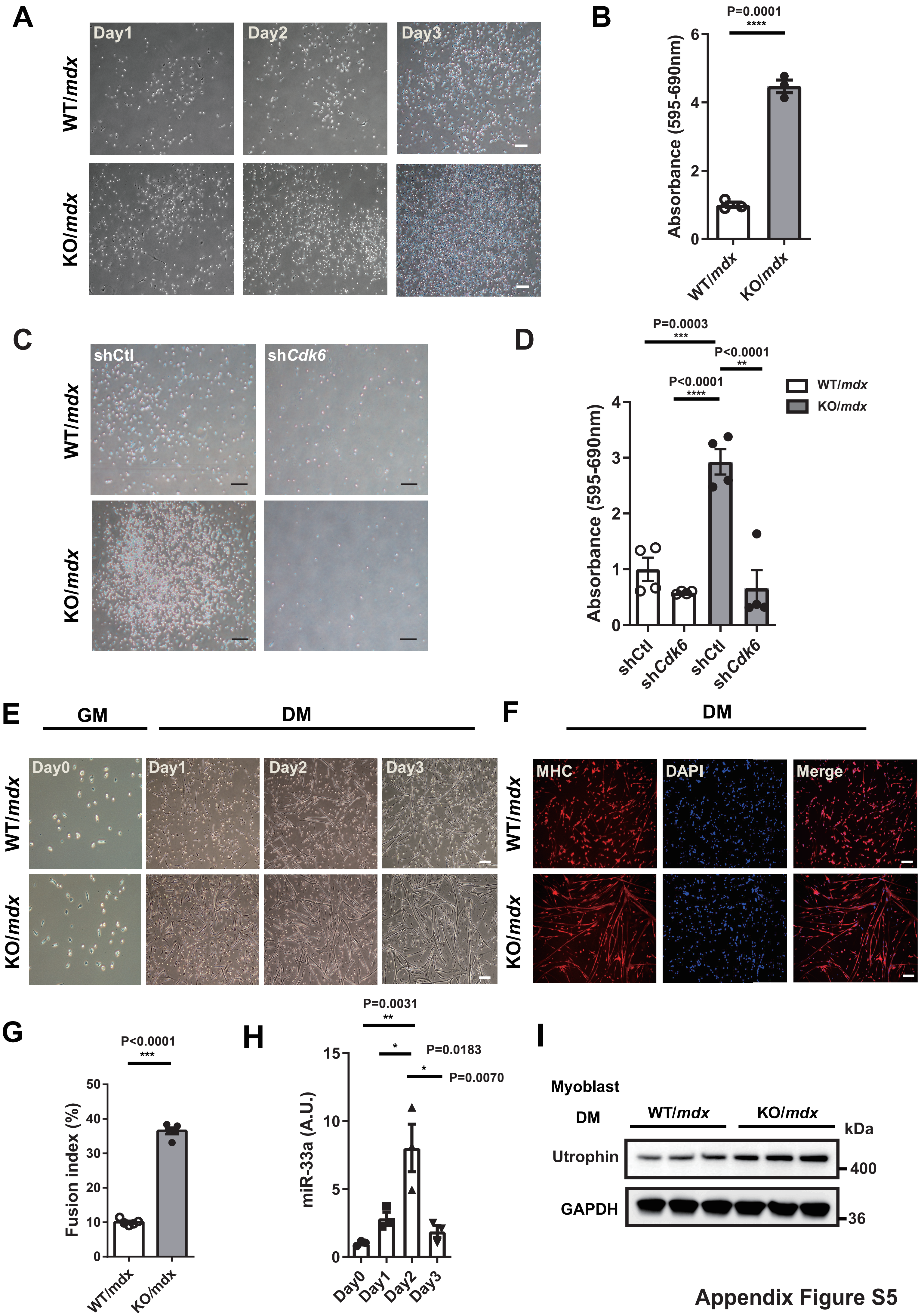

Appendix Figure S5

Appendix Figure S5. miR-33a deficiency increases myoblast expansion and differentiation. (A) Representative images of myoblasts from WT/*mdx* and KO/*mdx* mice cultured in GM for the indicated times. Scale bar: 50 μm. (B) MTT absorbance of myoblasts on day 3 of culture in GM ($n = 3$/group). Unpaired *t*-test. (C) Representative images of myoblasts from WT/*mdx* and KO/*mdx* mice in which shCtl and sh*Cdk6* were introduced by lentivirus and cultured in GM for 3 days. Scale bar: 50 μm. (D) MTT absorbance of myoblasts on day 3 of GM culture ($n = 4$/group). One-way ANOVA with Tukey post-hoc test. (E) Representative images of myoblasts from WT/*mdx* and KO/*mdx* mice differentiated into myotubes in DM for the indicated times. Scale bar: 50 μm. (F) Representative MHC immunostaining of myotubes differentiated from myoblasts of WT/*mdx* and KO/*mdx* mice for 3 days. Scale bar: 100 μm. (G) Fusion index of myotubes differentiated from myoblasts from the TA muscle of WT/*mdx* and KO/*mdx* mice for 3 days ($n = 5$/group). Unpaired *t*-test. (H) miR-33a expression during the course of myoblast differentiation ($n = 3$/group). One-way ANOVA with Tukey post-hoc test. (I) Utrophin expression in the differentiated myotubes from myoblasts of WT/*mdx* and KO/*mdx* mice ($n = 3$/group). Data are presented as the mean ± SEM. *$p < 0.05$, **$p < 0.01$, ***$p < 0.001$, ****$p < 0.0001$.

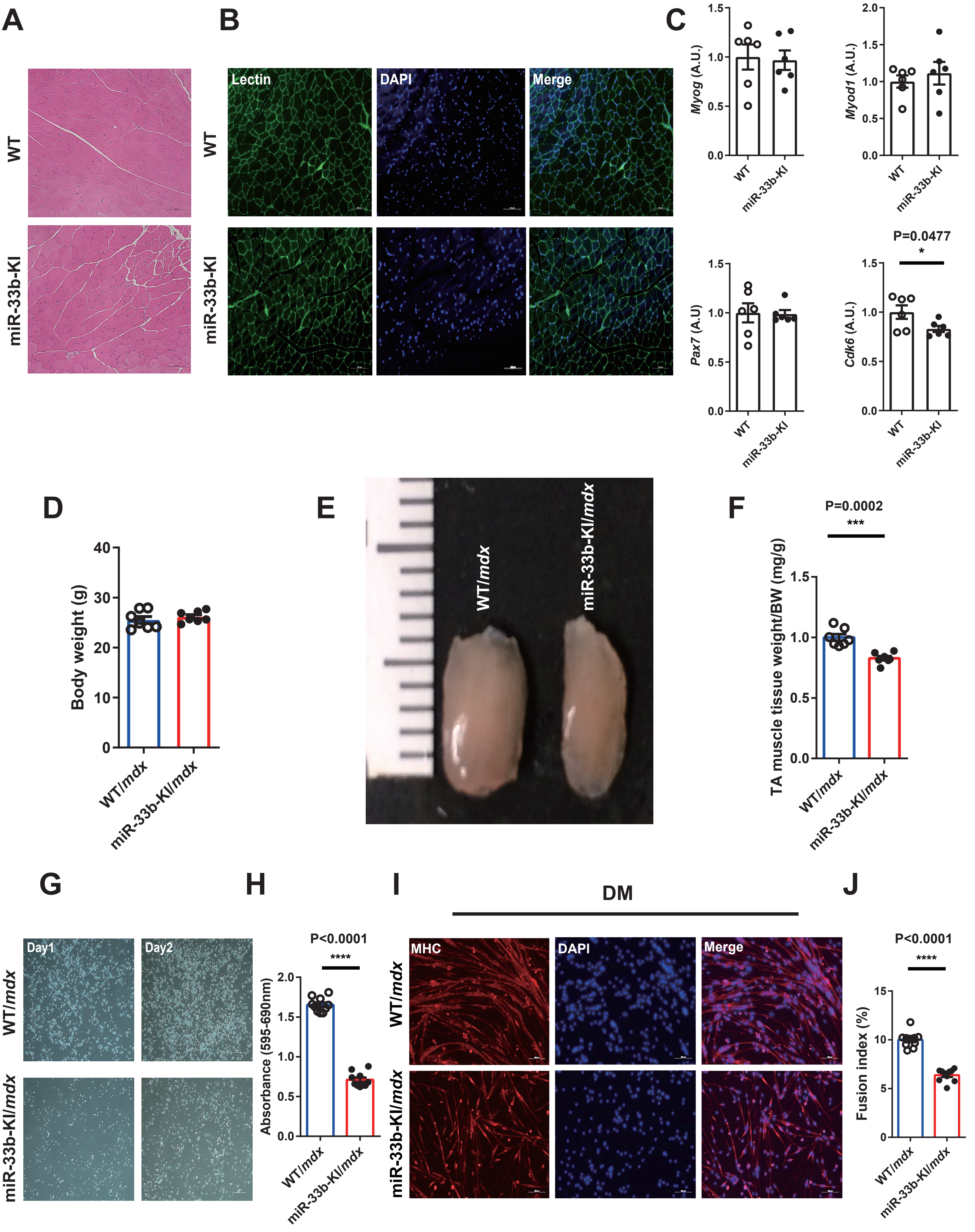

Appendix Figure S6

Appendix Figure S6. Generation of miR-33b knock-in and *mdx* mice (miR-33b-KI/*mdx* mice). (A) Representative images of HE staining of TA muscle of WT and miR-33b knock-in (miR-33b-KI) mice under a steady state. Scale bar: 100 µm. (B) Representative fluorescent images of TA muscle in WT and miR-33b-KI mice under a steady state stained with lectin and DAPI. Scale bar: 100 µm. (C) Expression of *Myog*, *Myod1*, *Pax7*, and *Cdk6* in TA muscle of WT and miR-33b-KI mice (n = 6/group). Unpaired *t*-test. (D) Body weight of 8-week-old WT/*mdx* and miR-33b-KI/*mdx* mice (n = 7/group). (E) Representative images of TA muscle from 8-week-old WT and miR-33b-KI mice. (F) Weight of TA muscle from 8-week-old WT and miR-33b-KI mice (n = 7/group). Unpaired *t*-test. (G) Representative images of myoblasts from WT/*mdx* and miR-33b-KI/*mdx* mice cultured in GM for the indicated time. Scale bar: 50 µm. (H) MTT absorbance of myoblasts from WT/*mdx* and miR-33b-KI/*mdx* mice on day 3 of culture in GM (n = 10/group). Unpaired *t*-test. (I) Representative MHC immunostaining of myotubes differentiated from myoblasts of WT/*mdx* and miR-33b-KI/*mdx* mice for 3 days. Scale bar: 100 µm. (J) Fusion index of myotubes differentiated from myoblasts of WT/*mdx* and miR-33b-KI/*mdx* mice after 3 days (n = 10/group). Unpaired *t*-test. Data are presented as the mean ± SEM. *p < 0.05, ***p < 0.001, ****p < 0.0001.

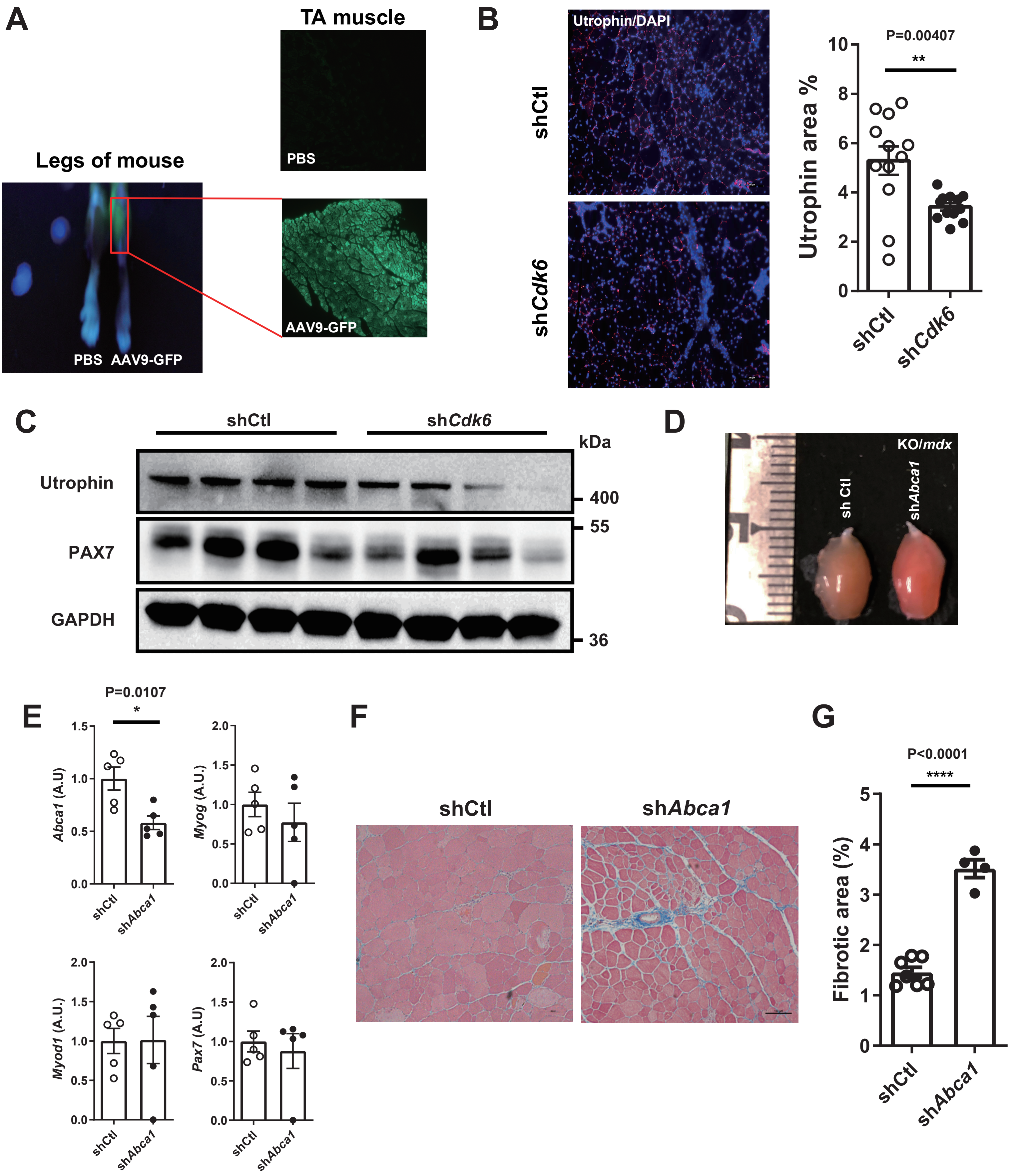

Appendix Figure S7

Appendix Figure S7. AAV9-mediated rescue experiments for *Cdk6* and *Abca1*. (A) Representative fluorescent images of leg and TA muscle injected with PBS or AAV9-GFP vector in WT mice. The mice were euthanized for analysis 1 week after AAV9 injection. (B, left) Representative fluorescent images of TA muscle from KO/*mdx* mice injected with AAV9 shCtl or sh*Cdk6* stained with utrophin and DAPI. Scale bar: 100 µm. (B, right) Quantification of utrophin-positive area in TA muscle from KO/*mdx* mice injected with AAV9 shCtl or sh*Cdk6*. Two fields of view/mouse (n = 6/group). Unpaired *t*-test. (C) Western blotting for utrophin, PAX7, and GAPDH in TA muscle from KO/*mdx* mice injected with AAV9 shCtl or sh*Cdk6*. (D) Representative images of TA muscle from KO/*mdx* mice injected with PBS, AAV9 shCtl, and AAV9 shRNA against *Abca1* (sh*Abca1*). (E) Expression of *Abca1*, *Myog*, *Myod1*, and *Pax7* in the TA muscle of KO/*mdx* mice injected with AAV9 shCtl or sh*Abca1* (n = 5/group) Unpaired *t*-test. (F) Representative images of Masson trichrome staining of TA muscle of KO/*mdx* mice injected with AAV9 shCtl or sh*Abca1*. Scale bars: 500 µm (left) and 100 µm (right). (G) Percentage of fibrotic area in the TA muscle of KO/*mdx* mice injected with AAV9 shCtl (n = 7) or sh*Abca1* (n = 4). Unpaired *t*-test. Data are presented as the mean ± SEM.*$p < 0.05$, **$p < 0.01$, ****$p < 0.0001$.

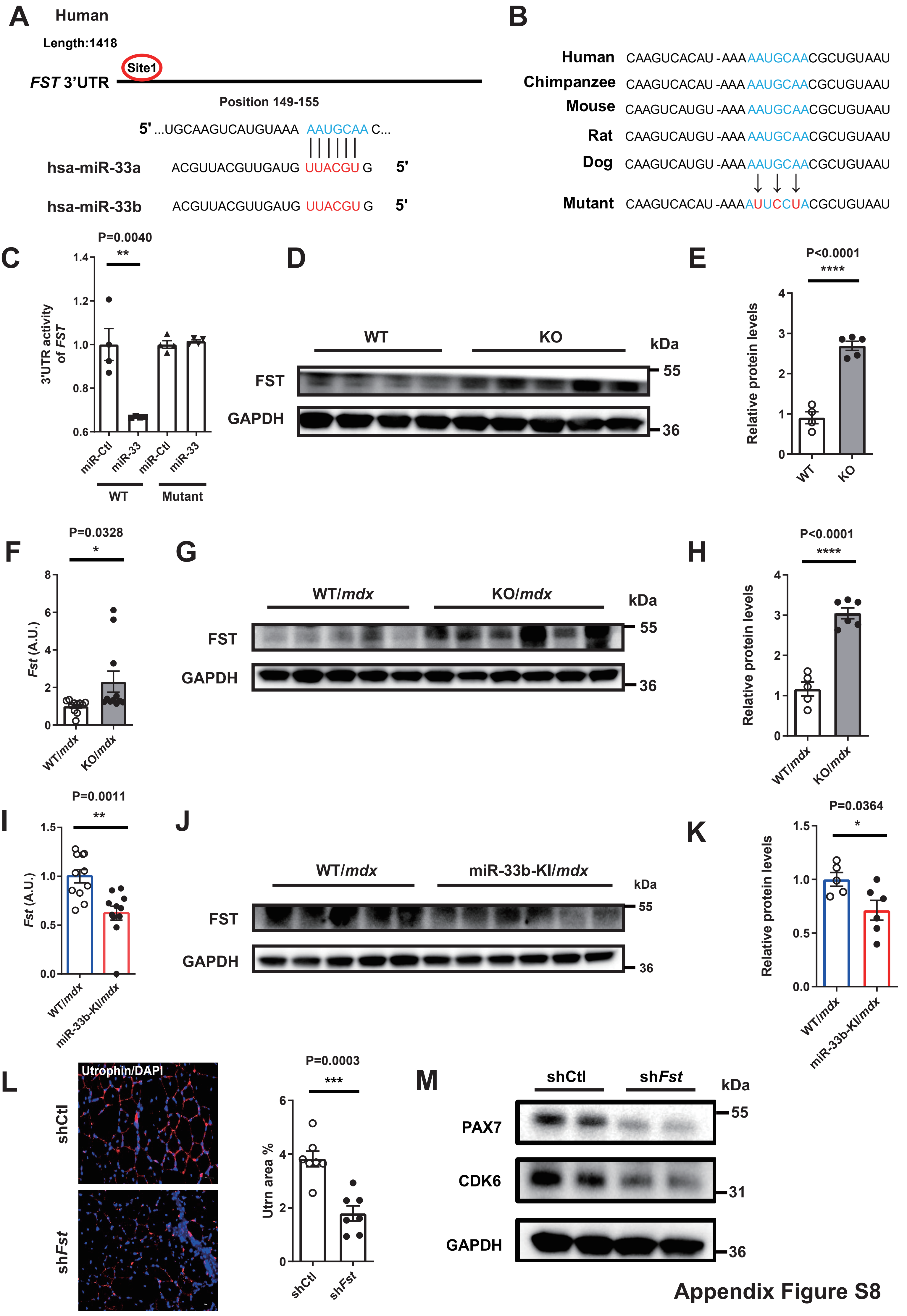

Appendix Figure S8

Appendix Figure S8. *Fst* is a target gene of miR-33. (A) Scheme of the miR-33 binding sites in the 3′-UTR of human *FST*. Blue sequences are potential binding sites of miR-33 seed sequences. (B) Conservation of miR-33 target regions in the 3′-UTR of *FST*. Blue sequences are potential binding sites of miR-33 seed sequences. Mutant 3′-UTR sequences are shown in red. (C) *FST* 3′-UTR luciferase reporter activity in HEK293T cells cotransfected with miR-Ctl and miR-33 (n = 4/group). Unpaired *t*-test. (D) Western blotting for FST and GAPDH in the TA muscle of WT and KO mice. (E) Densitometric analysis of FST levels in the TA muscle of WT (n = 4) and KO (n = 5) mice. Unpaired *t*-test. (F) *FST* expression in the TA muscle of WT/*mdx* and KO/*mdx* mice (n = 11/group). Unpaired *t*-test. (G) Western blotting for FST and GAPDH in the TA muscle of WT/*mdx* and KO/*mdx* mice. (H) Densitometric analysis of FST levels in the TA muscle of WT/*mdx* (n = 5) and KO/*mdx* (n = 6) mice. Unpaired *t*-test. (I) *Fst* expression in the TA muscle of WT/*mdx* (n = 11) and miR-33b-KI/*mdx* mice (n = 10). Unpaired *t*-test. (J) Western blotting for FST and GAPDH in the TA muscle of WT/*mdx* and miR-33b-KI/*mdx* mice. (K) Densitometric analysis of FST in the TA muscle of WT/*mdx* (n = 5) and miR-33b-KI/*mdx* (n = 6) mice. Unpaired *t*-test. (L, left) Representative fluorescent images of TA muscle from KO/*mdx* mice injected with AAV9 shCtl or sh*Fst* and stained with utrophin and DAPI. Scale bar: 50 μm. (L, right) Quantification of the utrophin-positive area (n = 7/group). Unpaired *t*-test. (M) Western blotting for PAX7, CDK6, and GAPDH in TA muscle of KO/*mdx* mice injected with AAV9 shCtl or sh*Fst*. Data are presented as the mean ± SEM. $^{*}p < 0.05$, $^{**}p < 0.01$, $^{***}p < 0.001$, $^{****}p < 0.0001$.

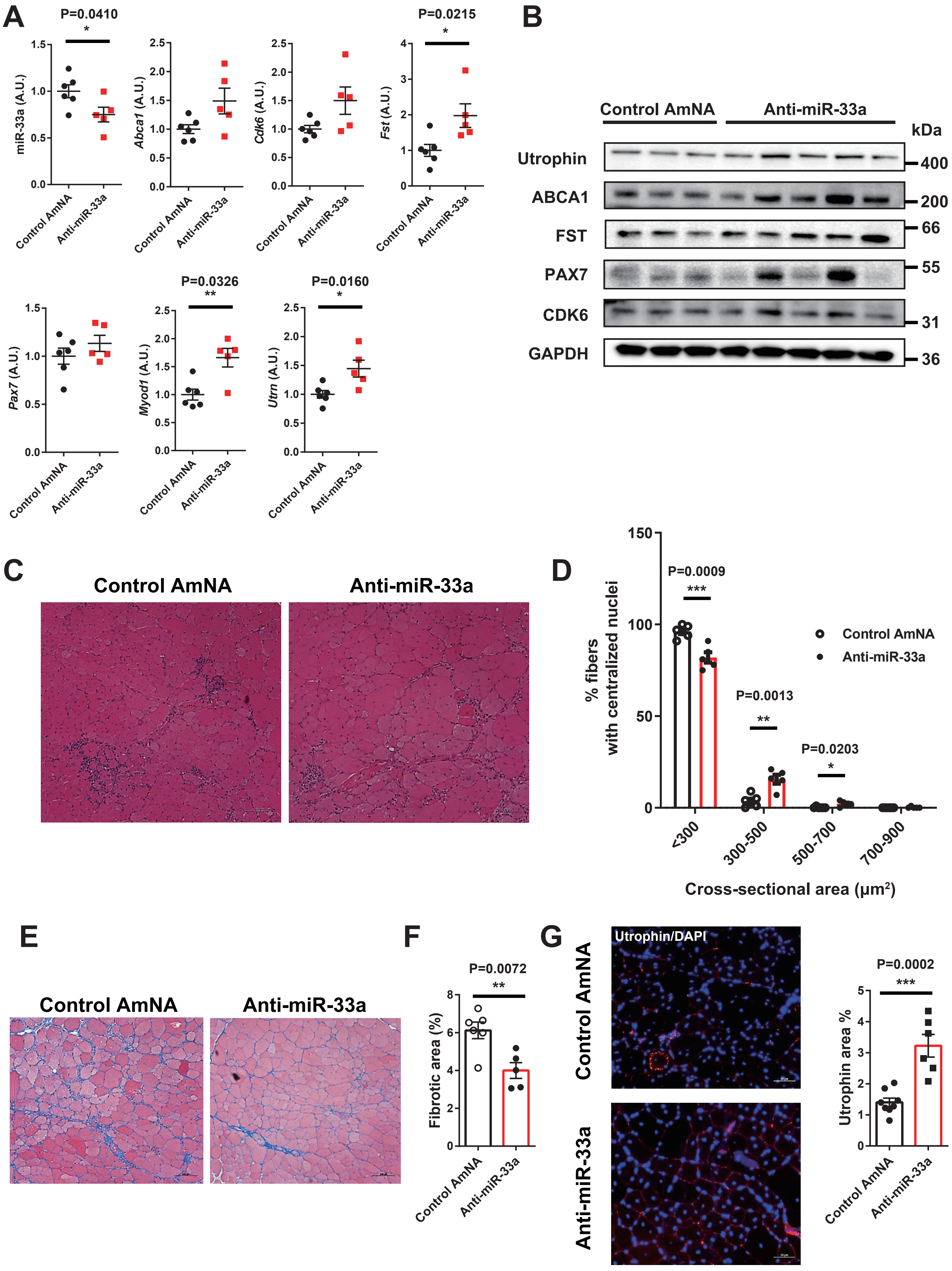

Appendix Figure S9

Appendix Figure S9. Local delivery of anti-miR-33a ameliorates dystrophic phenotypes in *mdx* mice. (A) Expression of miR-33a, *Abca1*, *Cdk6*, *Fst, Pax7*, *Myod1*, and *Utrn* in the TA muscle of *mdx* mice injected with control AmNA (n = 6) or anti-miR-33a (n = 5). Unpaired *t*-test. (B) Western blotting for utrophin, ABCA1, FST, PAX7, CDK6, and GAPDH in the TA muscle of *mdx* mice injected with control AmNA or anti-miR-33a. (C) Representative images of HE staining of TA muscle of *mdx* mice injected with control AmNA or anti-miR-33a. Scale bar: 100 µm. (D) Size distribution of muscle fibers in the TA muscle of *mdx* mice injected with control AmNA (n = 6) or anti-miR-33a (n = 5). Unpaired *t*-test. (E) Representative images of Masson trichrome staining of TA muscle of *mdx* mice injected with control AmNA or anti-miR-33a. Scale bar: 100 µm. (F) Percentage of fibrotic area in the TA muscle of *mdx* mice injected with control AmNA (n = 6) or anti-miR-33a (n = 5). Unpaired *t*-test. (G, left) Representative fluorescent images of TA muscle from *mdx* mice injected with control AmNA or anti-miR-33a and stained with utrophin and DAPI. Scale bar: 50 µm. (G, right) Quantification of the utrophin-positive area in *mdx* mice injected with control AmNA (n = 8) or anti-miR-33a (n = 6). Unpaired *t*-test. Data are presented as the mean ± SEM. *$p < 0.05$, **$p < 0.01$, ***$p < 0.001$.

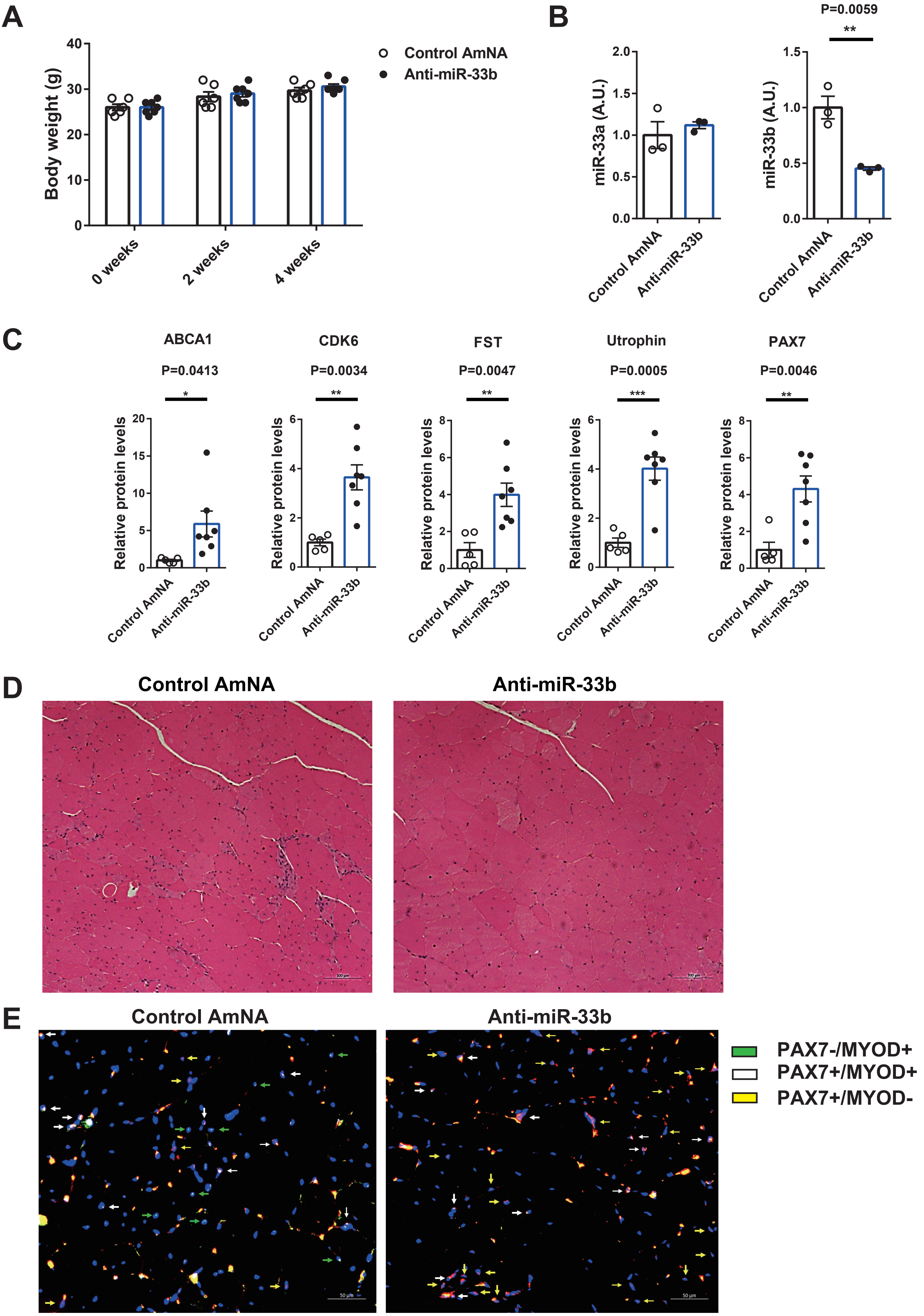

Appendix Figure S10

Appendix Figure S10. Effect of systemic administration of anti-miR-33b at a dose of 20 mg/kg bw. (A) Body weight changes in miR-33b-KI/*mdx* mice injected with 20 mg/kg bw control AmNA ($n = 6$) or anti-miR-33b ($n = 7$). (B) Expression of miR-33a and miR-33b in GAS muscle after subcutaneous injection of control AmNA or anti-miR-33b ($n = 3$/group). Unpaired t-test. (C) Densitometric analysis of ABCA1, utrophin, PAX7, FST, and CDK6 in the TA muscle of miR-33b-KI/*mdx* mice treated with control AmNA ($n = 5$) or anti-miR-33b ($n = 7$). Unpaired *t*-test. (D) Representative images of HE staining of the TA muscle of miR-33b-KI/*mdx* mice treated with control AmNA or anti-miR-33b. Scale bar: 100 µm. (E) Representative fluorescent images of TA muscle of miR-33b-KI/*mdx* mice injected with control AmNA or anti-miR-33b and stained with MyoD, Pax7, and DAPI. Green, white and yellow arrows indicate $Pax7^{-}/MyoD^{+}$, $Pax7^{+}/MyoD^{+}$, and $Pax7^{+}/MyoD^{-}$ cells, respectively. Scale bar: 50 µm. Data are presented as the mean ± SEM. *$p < 0.05$, **$p < 0.01$, ***$p < 0.001$.

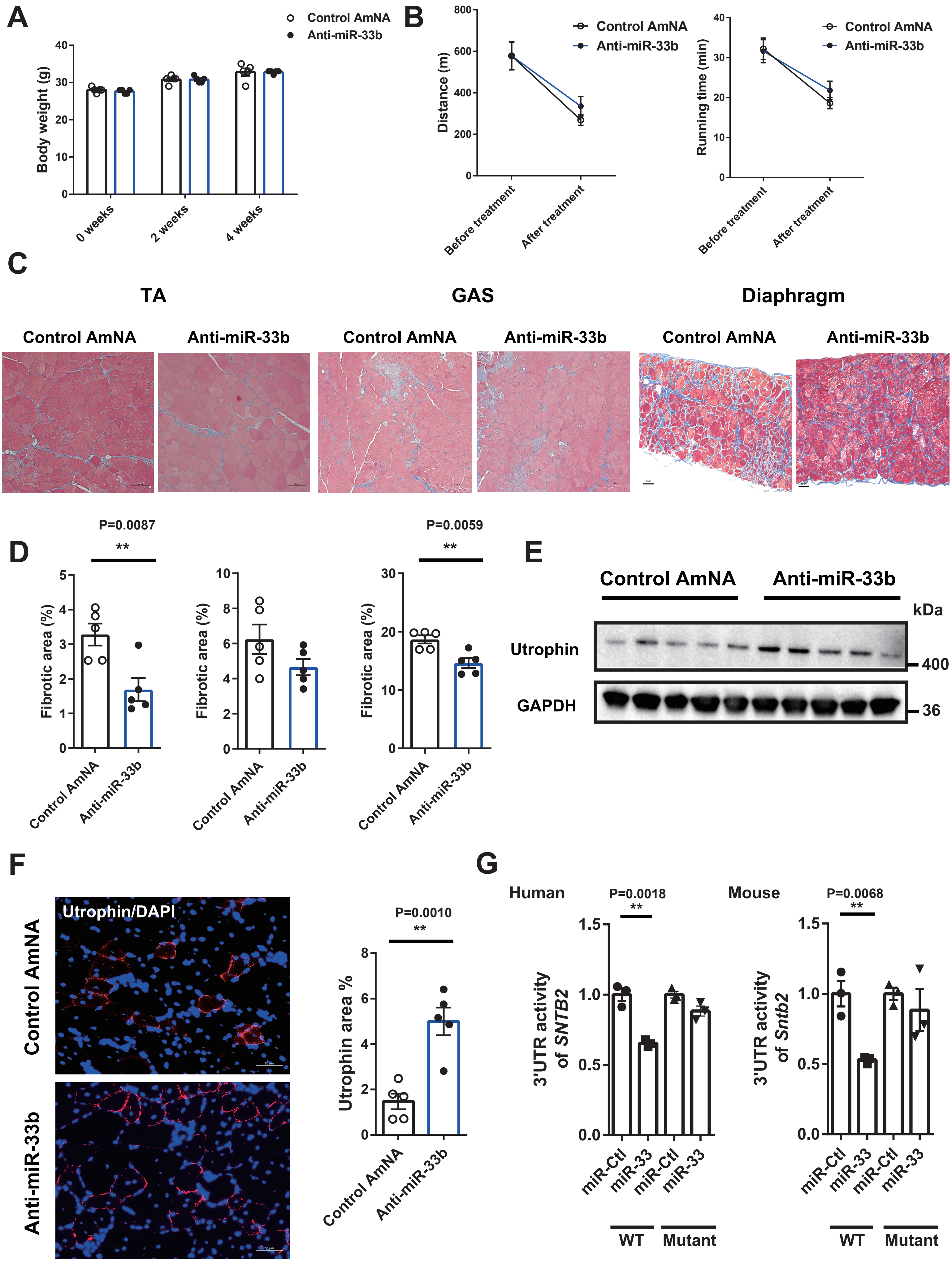

Appendix Figure S11

Appendix Figure S11. Effect of systemic administration of anti-miR-33b at a dose of 10 mg/kg bw. (A) Body weight changes in miR-33b-KI/*mdx* mice injected with control AmNA or anti-miR-33b at a dose of 10 mg/kg bw (n = 5/group). (B) Changes in running distance and running time during the treadmill endurance test in miR-33b-KI/*mdx* mice treated with control AmNA or anti-miR-33b (n = 5/group). (C) Representative images of Masson trichrome staining of TA muscle, GAS muscle, and diaphragm of miR-33b-KI/*mdx* mice treated with control AmNA or anti-miR-33b. Scale bars: 100 μm (left and middle) and 50 μm (right). (D) Percentage of fibrotic area in the TA muscle, GAS muscle, and diaphragm of miR-33b-KI/*mdx* mice treated with control AmNA or anti-miR-33b (n = 5/group). Unpaired *t*-test. (E) Western blotting for utrophin and GAPDH levels in TA muscle from miR-33b-KI/*mdx* mice treated with control AmNA or anti-miR-33b. (F, left) Representative fluorescent images of TA muscle from miR-33b-KI/*mdx* mice treated with control AmNA or anti-miR-33b and stained with utrophin and DAPI. Scale bar: 50 μm. (F, right) Quantification of the utrophin-positive area of TA muscle from miR-33b-KI/*mdx* mice treated with control AmNA or anti-miR-33b (n = 5/group). Unpaired *t*-test. (G) Human and mouse *SNTB2* 3′-UTR luciferase reporter activity in HEK293T cells cotransfected with miR-Ctl or miR-33 (n = 3/group). Unpaired *t*-test. Data are presented as the mean ± SEM. *$p < 0.05$, **$p < 0.01$.

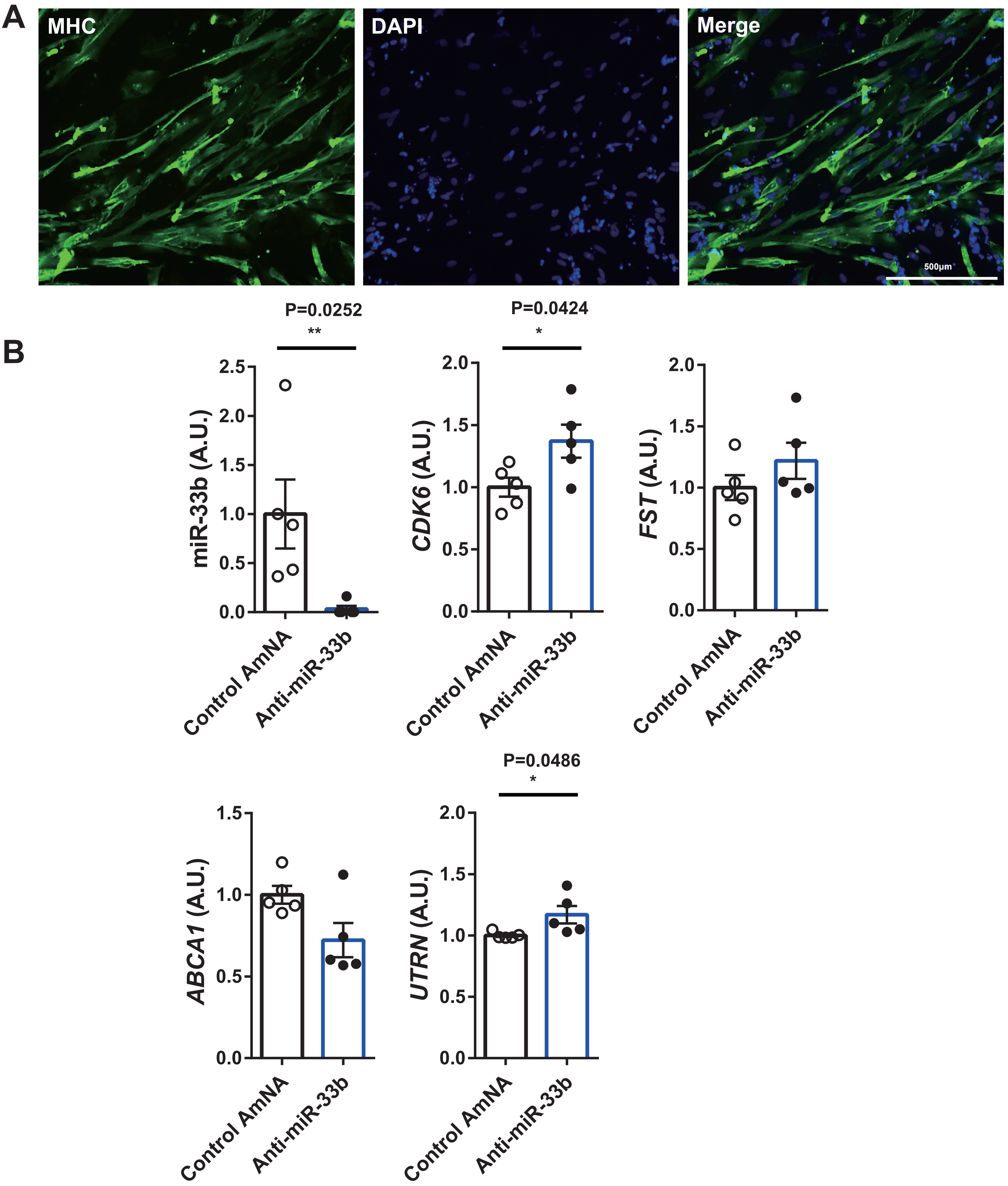

Appendix Figure S12. Analysis of myotubes differentiated from DMD patient-derived iPS cells treated with anti-miR-33b. (A) Representative fluorescent images of myotubes differentiated from human iPS cells of a patient with DMD (CiRA00111) stained with MHC and DAPI. Scale bar: 500 µm. (B)Expression of miR-33b, *CDK6*, *FST,* *ABCA1,* and *UTRN* in myotubes differentiated from human iPS cells of a patient with DMD (CiRA00111) treated with control-AmNA and anti-miR-33b (n = 5/group). Unpaired *t*-test. Data are presented as the mean ± SEM. $^{*}p < 0.05$

Appendix Table S1. Serum data of mice administered AMOs dose of 10mg/kg bw.

	Control AmNA	Anti-miR-33b	
AST (IU/L)	693 ± 264	504 ± 191	
ALT (IU/L)	113 ± 23	101 ± 33	
LDH (IU/L)	3927 ± 1836	1914 ± 799	
CK (IU/L)	5629 ± 3898	2846 ± 1483	
T-CHO (mg/dL)	64 ± 5.3	89 ± 4.9	★★★★
HDL-C (mg/dL)	37 ± 2.1	47 ± 1.6	★★★★

Serum was obtained from miR-33b-KI mdx mice systemically administered with 10 mg/kg of control AmNA or anti-miR-33b weekly for 4weeks. (n = 5 group). Unpaired t-test . **** $P<0.0001$.

Appendix Table S2. List of upregulated genes by anti-miR33b AMO, which are predicted as miR-33 target genes.

Gene_ID	Transcript_ID	Gene_Symbol
15364	NM_010441	Hmga2
329154	NM_001081433	Ankrd44
338349	NM_177385	Cntln
56177	NM_001038612	Olfm1
12571	NM_009873	Cdk6
74145	NM_028784	F13a1
75974	XM_006541406	Dock11
192198	NM_138682	Lrrc4
14017	NM_001033711	Evi2a
12894	NM_013495	Cpt1a
54608	NM_018811	Abhd2
20750	NM_001204201	Spp1
68169	NM_172399	Ndnf
13003	NM_001081249	Vcan
67216	NM_026037	Mboat2
238252	NM_181752	Gpr135
16576	NM_001291222	Kif7
16172	NM_008359	Il17ra
14130	NM_001077189	Fcgr2b
235442	NM_173413	Rab8b
11695	NM_007442	Alx4
12289	NM_001083616	Cacna1d
14009	NM_001347379	Etv1
53623	NM_001281929	Gria3
11551	NM_007417	Adra2a
16764	NM_001290814	Aff3
23969	NM_001286743	Pacsin1
19221	NM_011197	Ptgfrn
20288	NM_031195	Msr1
18605	NM_001308327	Enpp1
72391	NM_001360041	Cdkn3
68404	NM_153529	Nrn1
13797	NM_010132	Emx2
12447	NM_007633	Ccne1
14313	NM_001301373	Fst
73173	NM_130448	Pcdh18
329324	NM_001301370	Syt14

Appendix Table S3. Details of primer pairs

Human

Target gene	Forward Primer	Reverse Primer
ACTB	AGGCACTCTTCCAGCCTTCC	GCACTGTGTTGGCGTACAGG
SREBF1	AACAGTCCCACTGGTCGTAGAT	TGTTGCAGAAAGCGAATGTAGT
SREBF2	AACGGTCATTCACCCAGGTC	GGCTGAAGAATAGGAGTTGCC

Mouse

Pax7	GAGTTCGATTAGCCGAGTGC	GTCGGGTTCTGATTCCACAT
Myog	CTTGCTCAGCTCCCTCAACC	GTTGGGACCGAACTCCAGTG
Myod1	CTTCTACGCACCTGGACCG	ACTGTAGTAGGCGGTGTCGT
Srebf1	TAGAGCATATCCCCCAGGTG	GGTACGGGCCACAAGAAGTA
Srebf2	GTGGAGCAGTCTCAACGTCA	TGGTAGGTCTCACCCAGGAG
Cdk6	TGTTTCAGCTTCTCCGAGGT	CTGGACTGGAGCAGGACTTC
Abca1	AACAGTTTGTGGCCCTTTTG	AGTTCCAGGCTGGGGTACTT
Fst	ATGGACCGAGGAGGATGTGA	TTGCATCTGGCCTTGAGGAG
Utrn	GCCCTCCCTGCAGATTATTTGG	CTGTCCAGTTGACCTTTGATACTCTTC
Gapdh	AAATGGTGAAGGTCGGTGTG	AATCTCCACTTTGCCACTGC
