# Supplementary figures and images for: MicroRNA-33 inhibition ameliorates muscular dystrophy by enhancing skeletal muscle regeneration
Source: EMBO Mol Med. 2025 Jul 23;17(8):1902–25. doi: 10.1038/s44321-025-00273-9 (PMC12340133; doi:10.1038/s44321-025-00273-9)

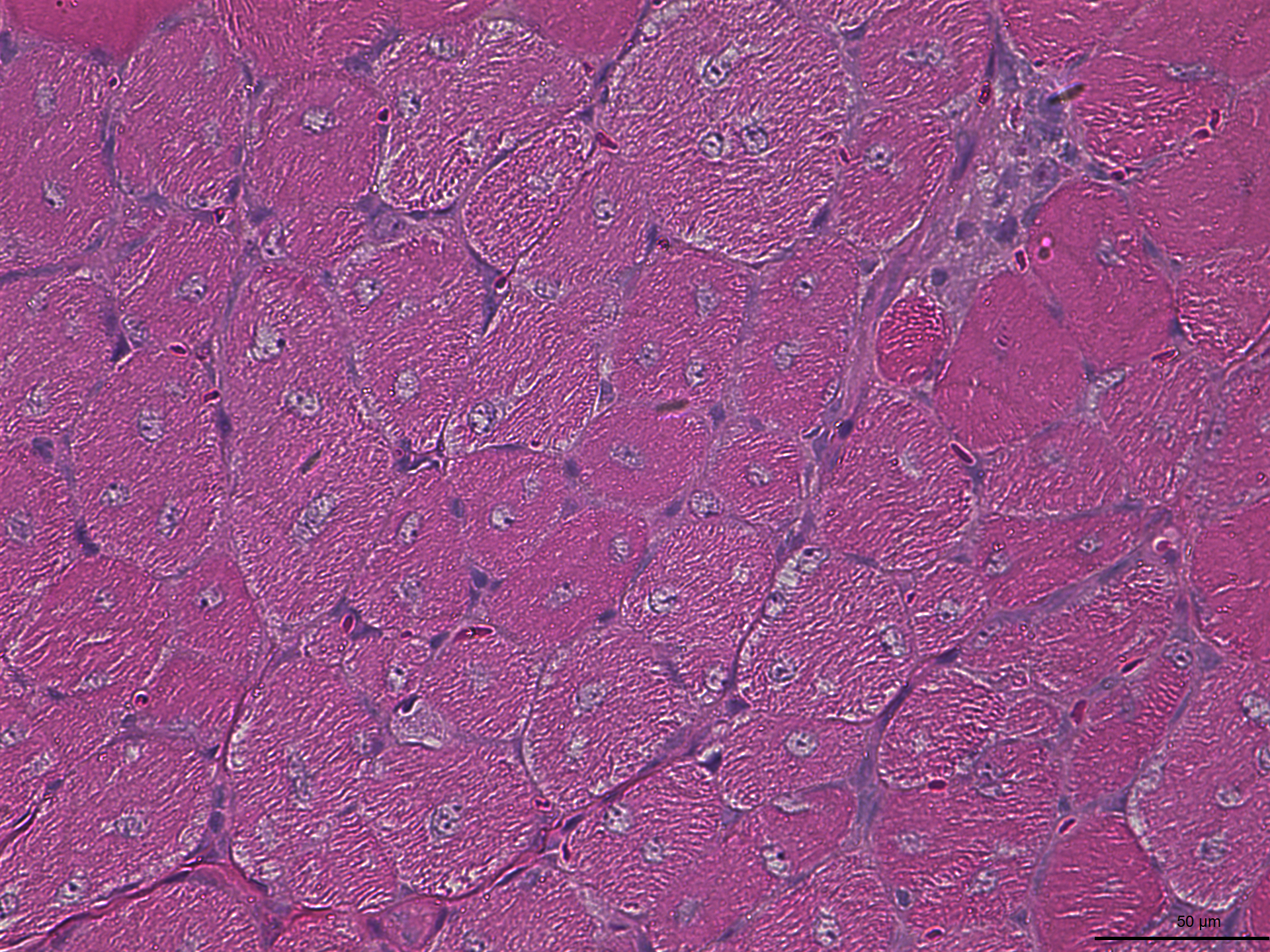

Supplement: Supplementary file 3 — Source data Fig. 1 [file 44321_2025_273_MOESM3_ESM.zip › Fig.1/Fig1-A/WT-HE.tif]

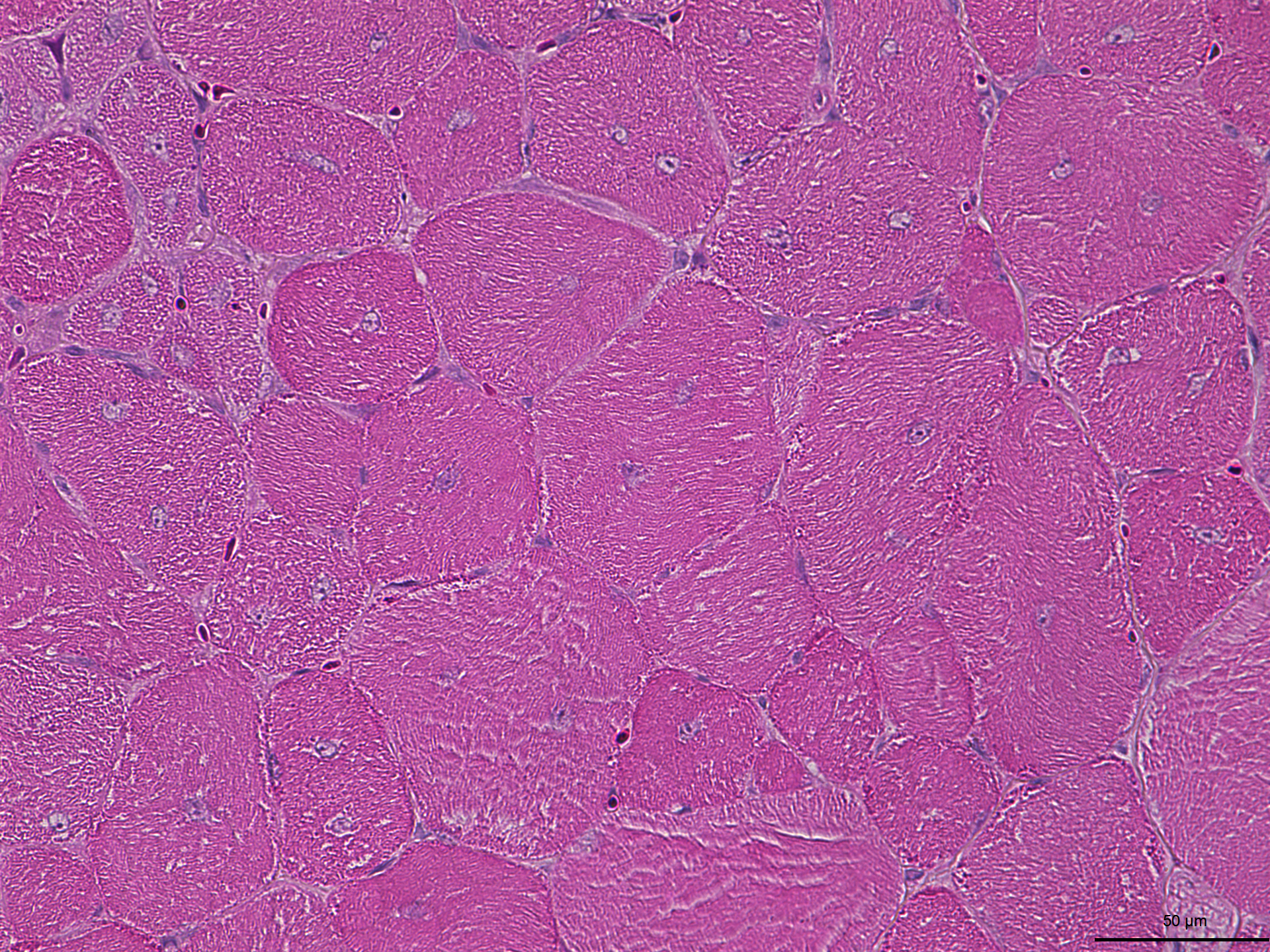

Supplement: Supplementary file 3 — Source data Fig. 1 [file 44321_2025_273_MOESM3_ESM.zip › Fig.1/Fig1-A/KO-HE.tif]

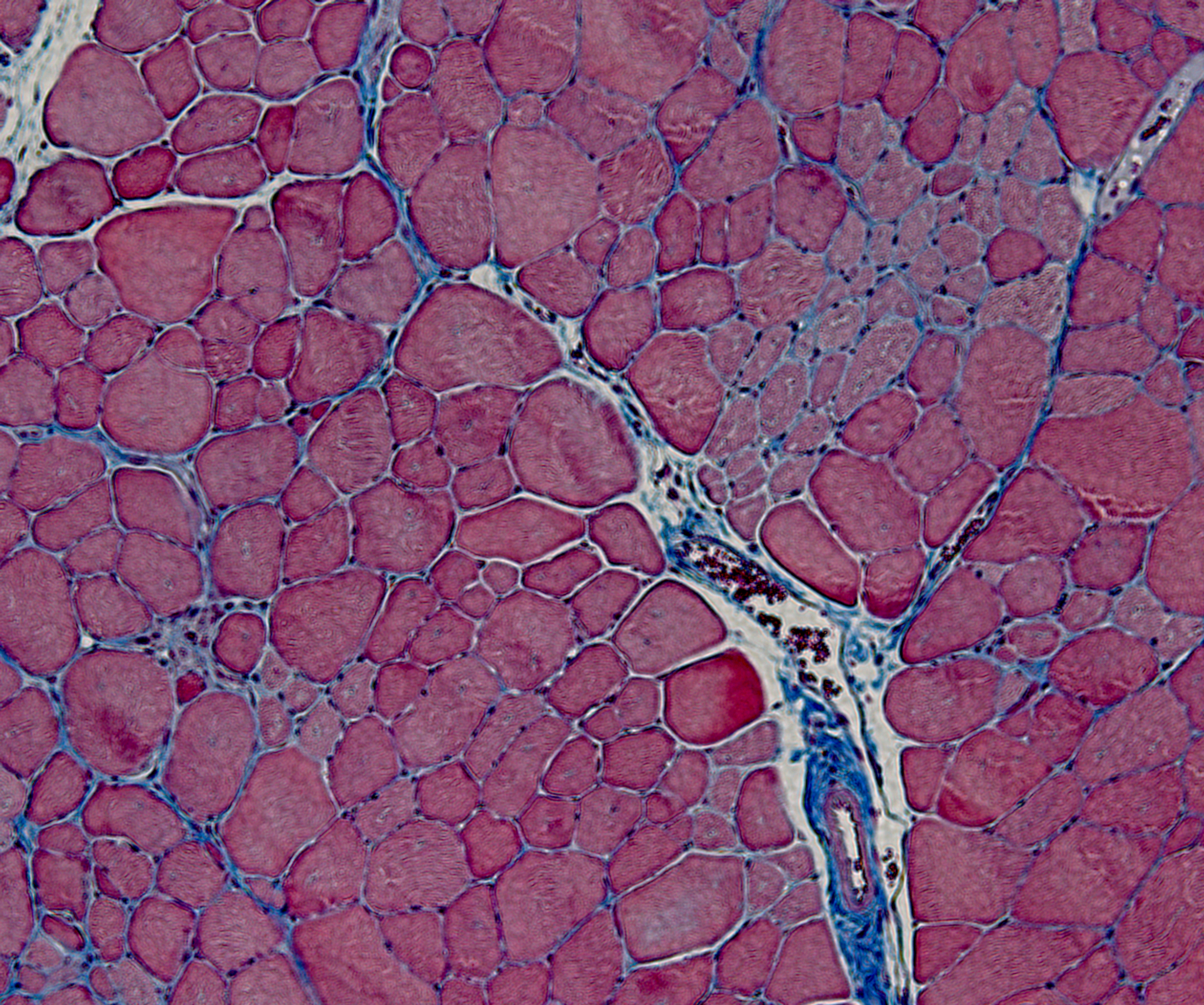

Supplement: Supplementary file 3 — Source data Fig. 1 [file 44321_2025_273_MOESM3_ESM.zip › Fig.1/Fig1-C/WT-mdx.tif]

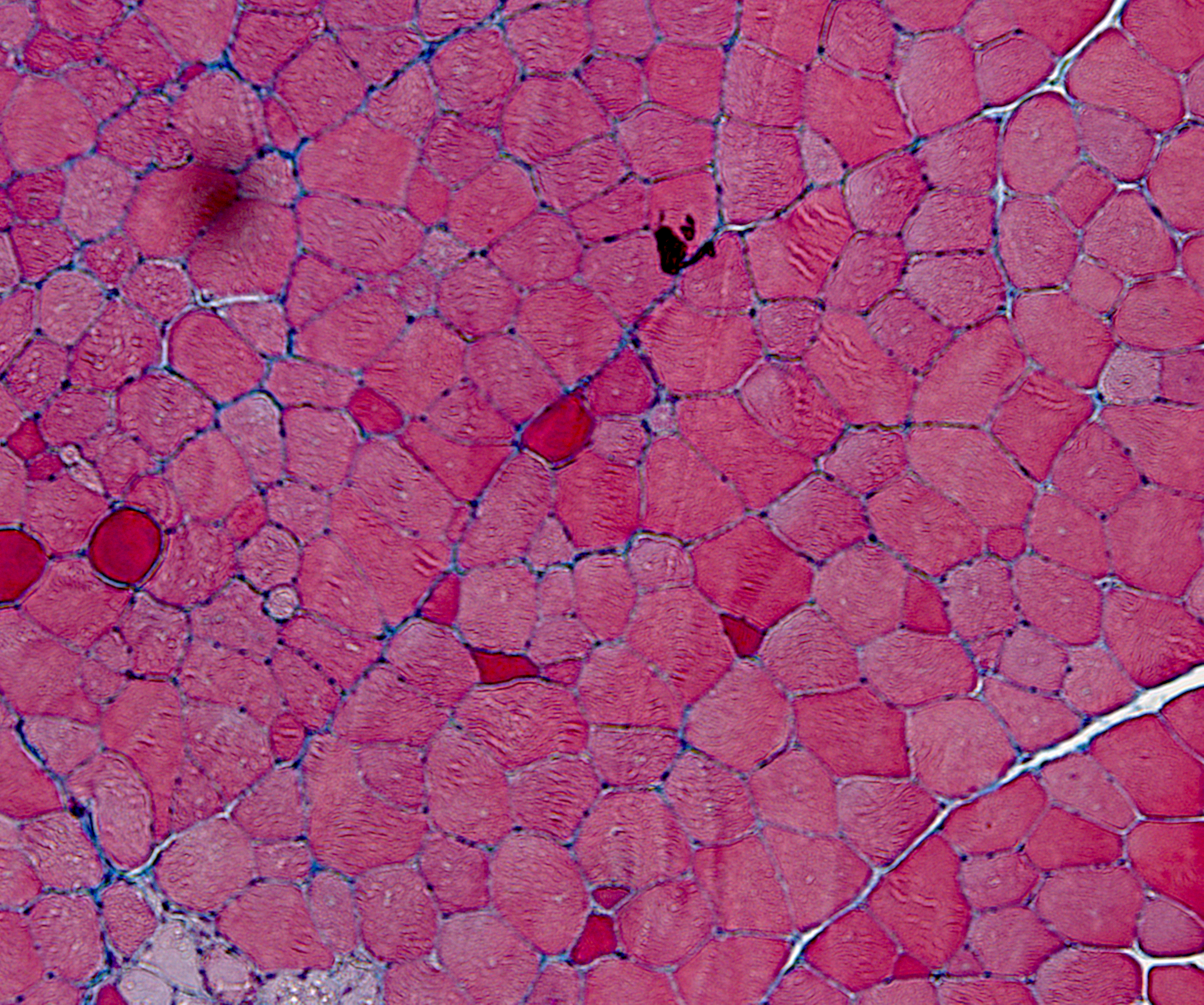

Supplement: Supplementary file 3 — Source data Fig. 1 [file 44321_2025_273_MOESM3_ESM.zip › Fig.1/Fig1-C/KO-mdx.tif]

Fig1-K

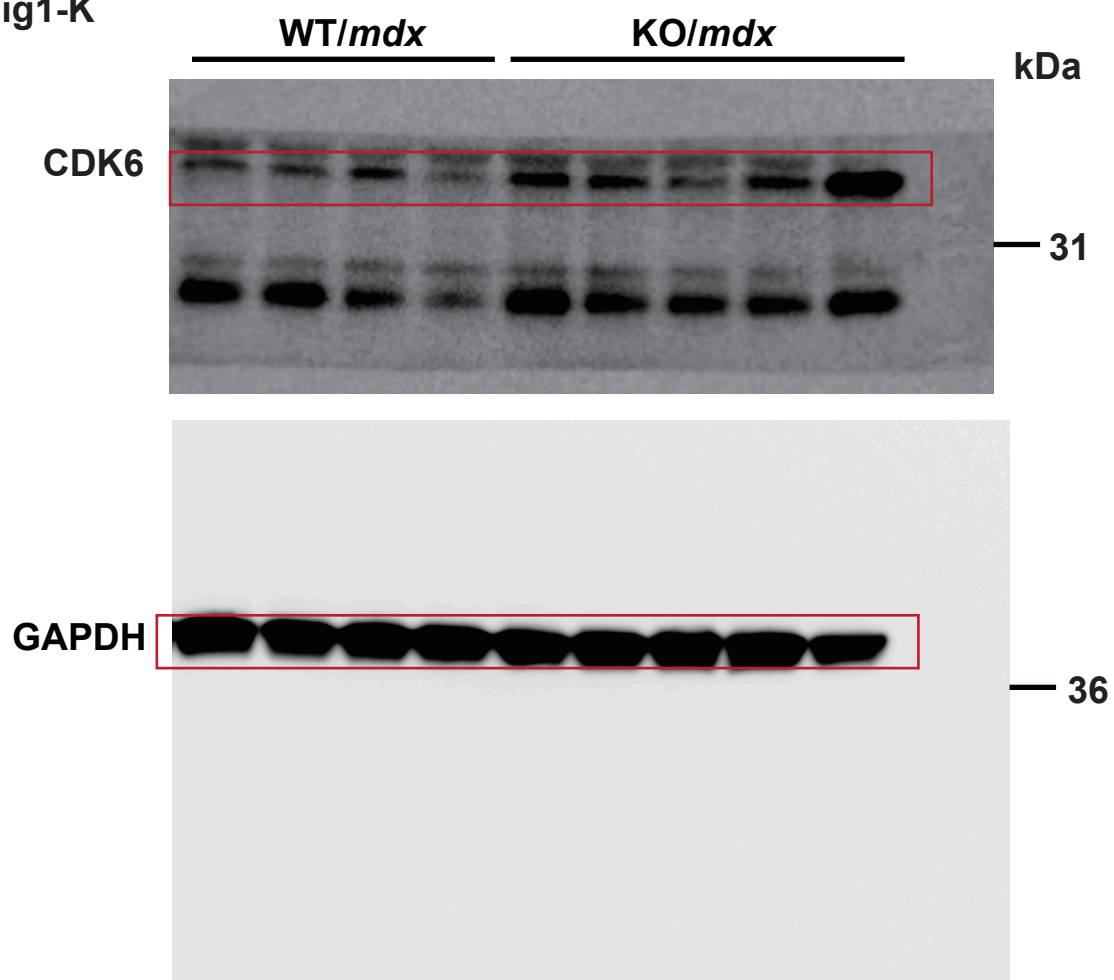

Supplement: Supplementary file 3 — Source data Fig. 1 [file 44321_2025_273_MOESM3_ESM.zip › Fig.1/Fig1-K/source-data-Fig1.pdf]

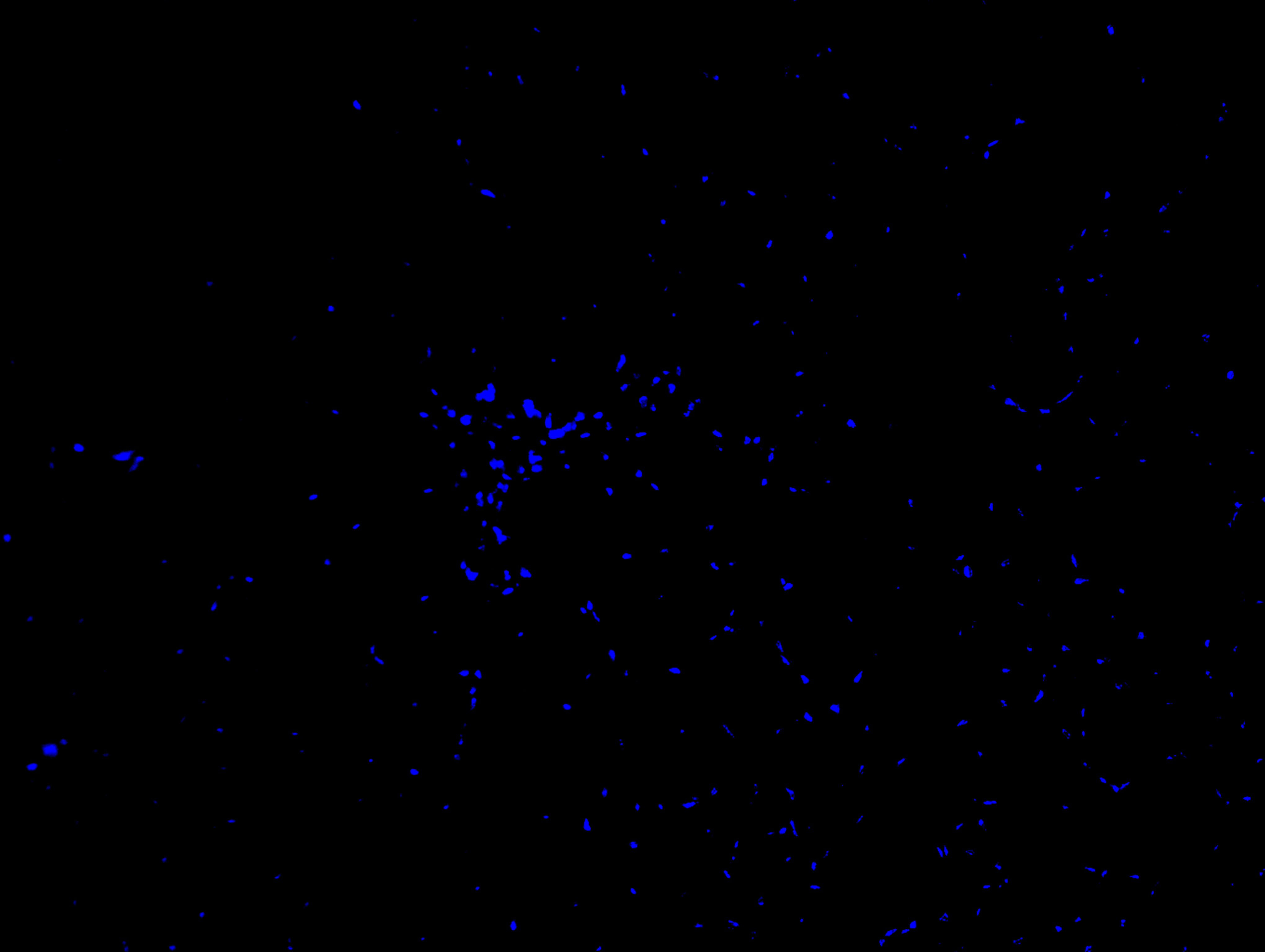

Supplement: Supplementary file 4 — Source data Fig. 2 [file 44321_2025_273_MOESM4_ESM.zip › Fig.2/Fig2-F/KO-mdx_DAPI.tif]

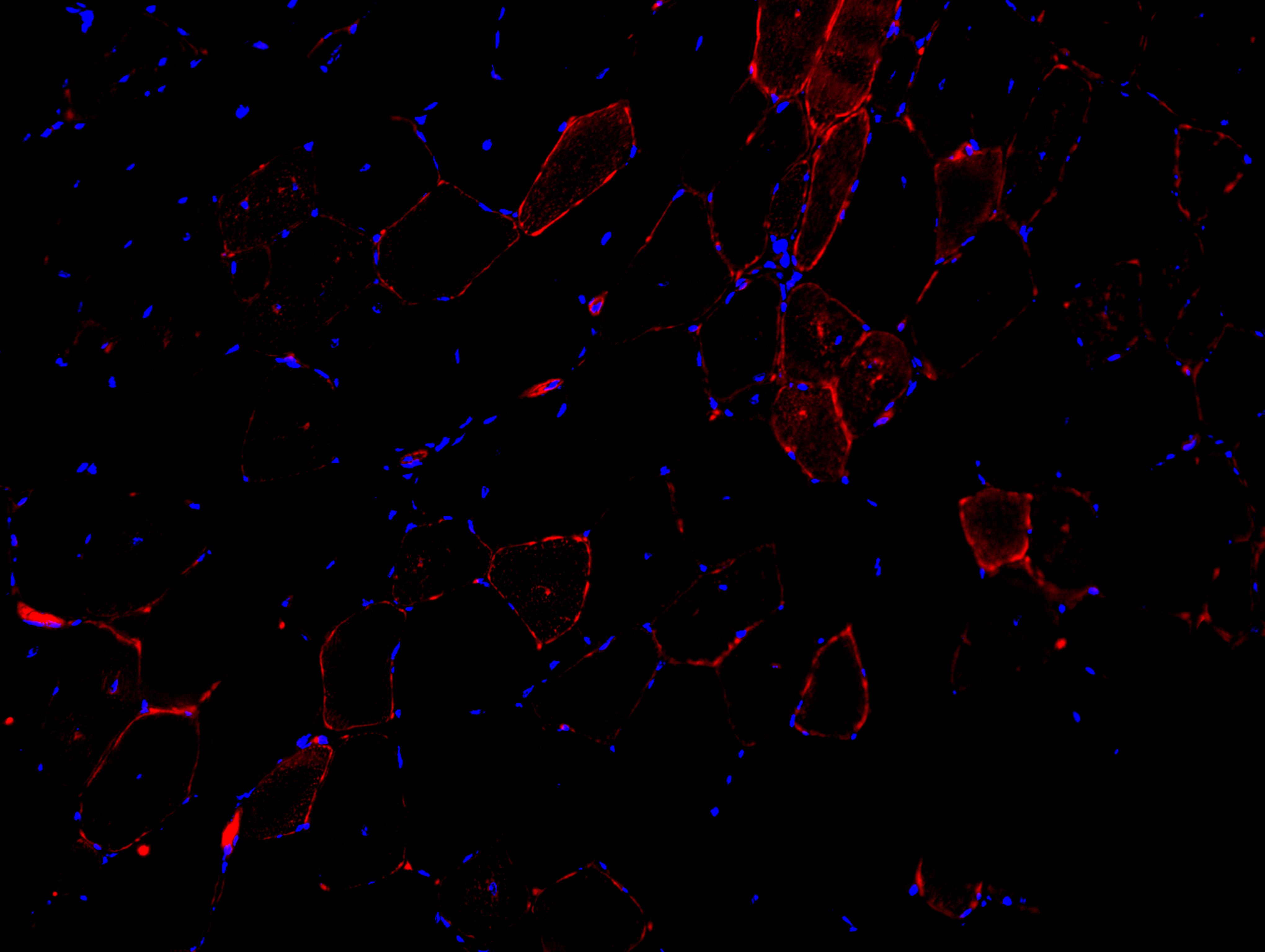

Supplement: Supplementary file 4 — Source data Fig. 2 [file 44321_2025_273_MOESM4_ESM.zip › Fig.2/Fig2-F/WT-mdx_Merge.tif]

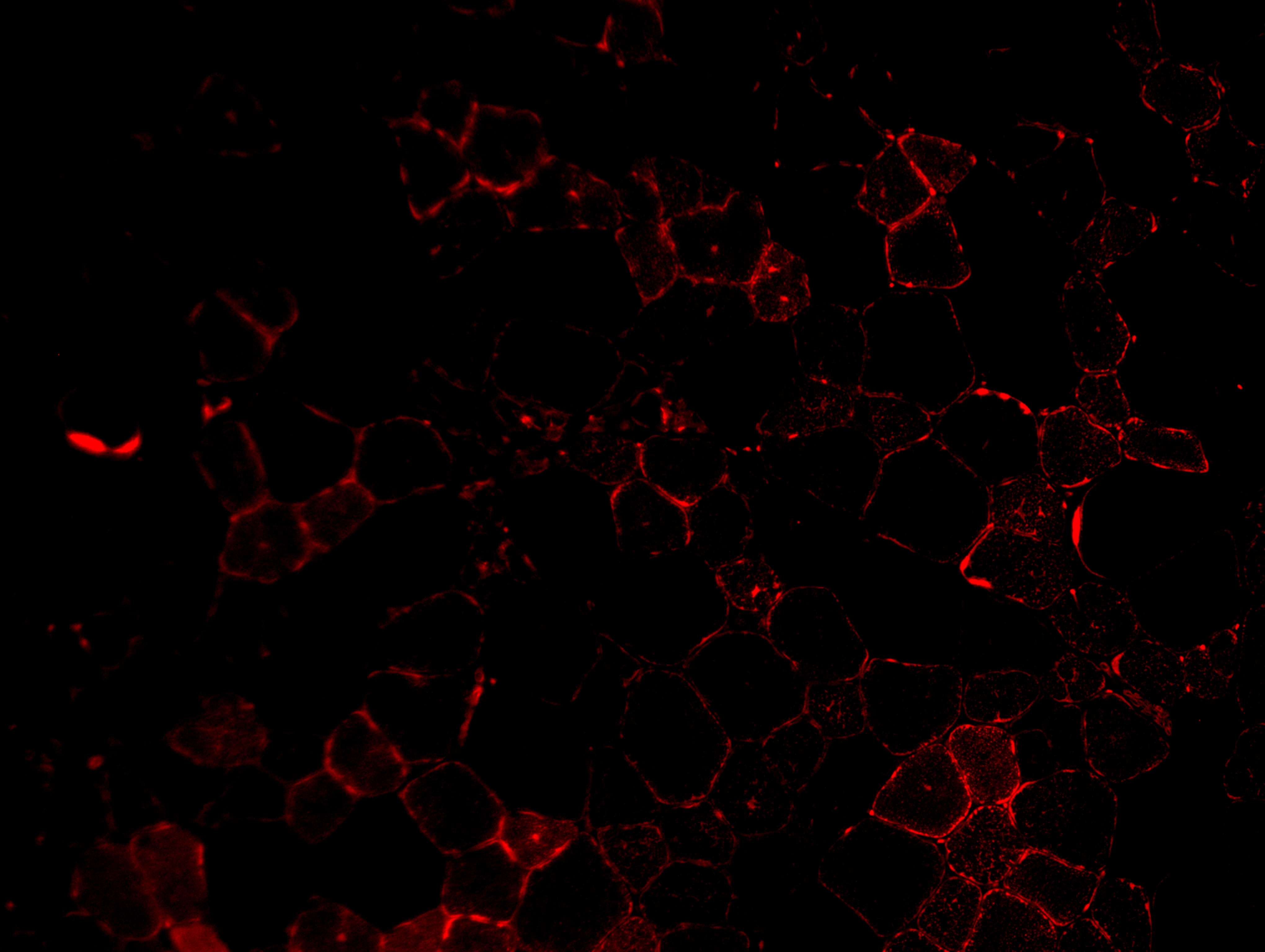

Supplement: Supplementary file 4 — Source data Fig. 2 [file 44321_2025_273_MOESM4_ESM.zip › Fig.2/Fig2-F/KO-mdx_Pax7.TIF]

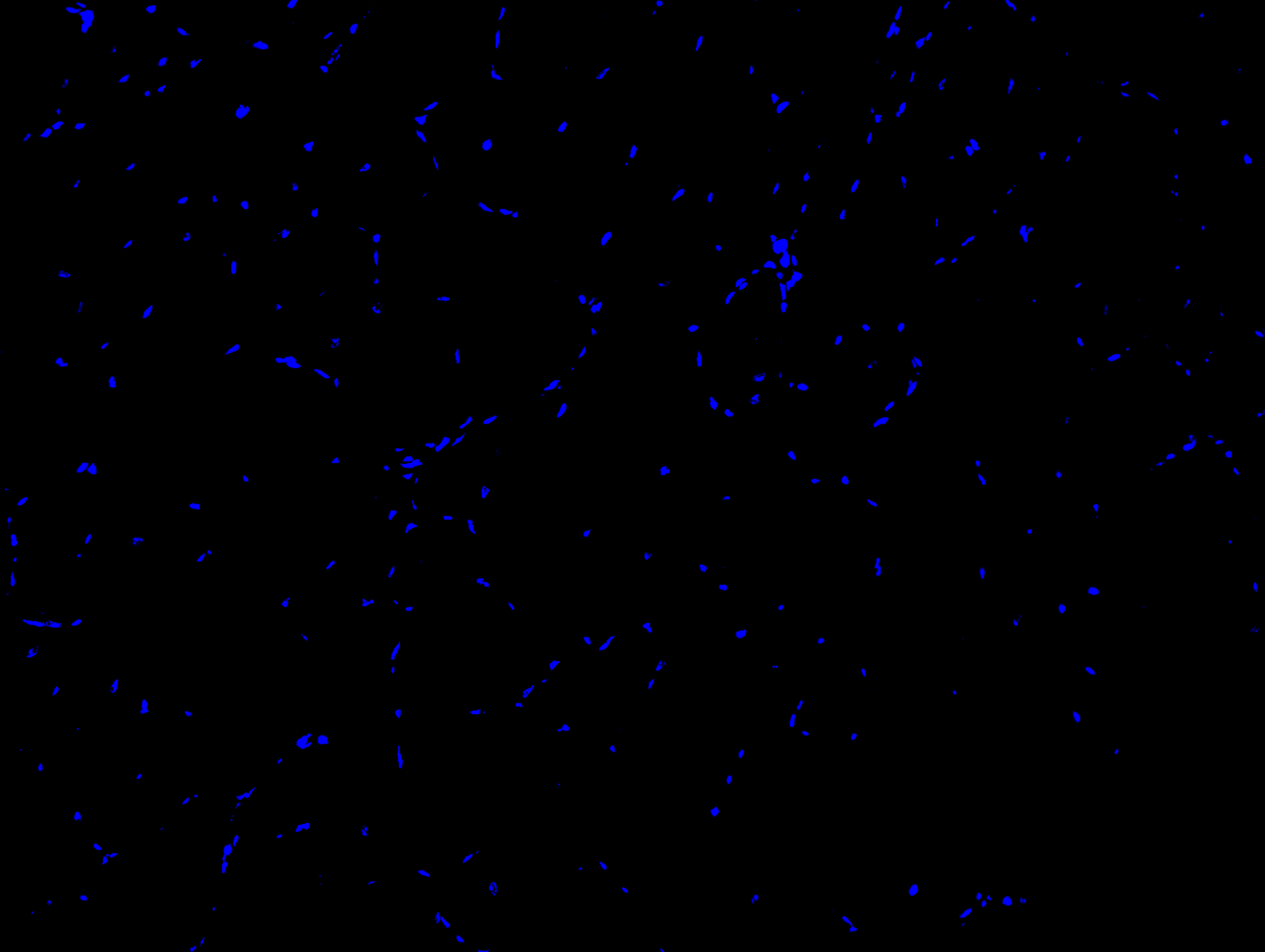

Supplement: Supplementary file 4 — Source data Fig. 2 [file 44321_2025_273_MOESM4_ESM.zip › Fig.2/Fig2-F/WT-mdx_DAPI.tif]

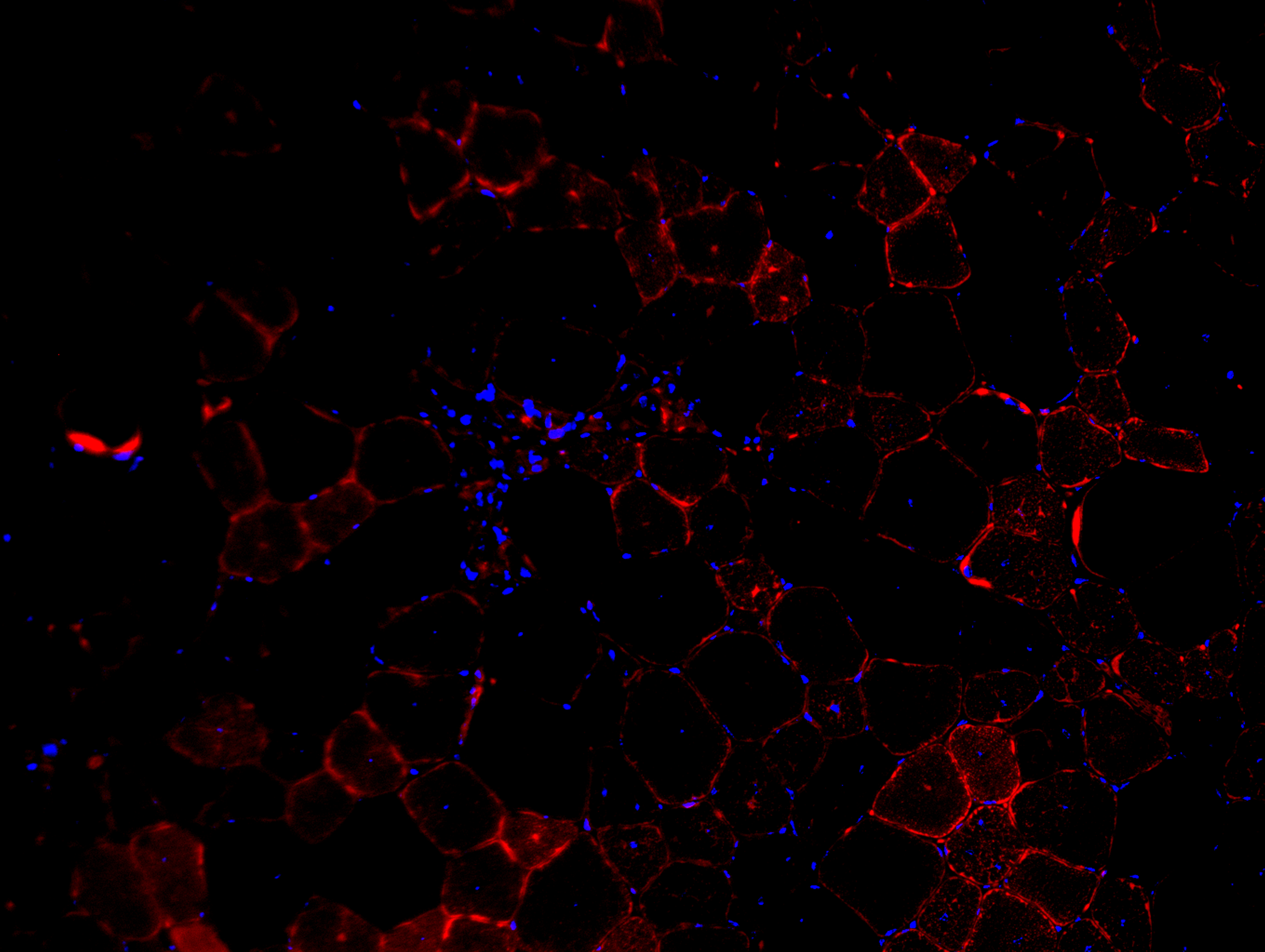

Supplement: Supplementary file 4 — Source data Fig. 2 [file 44321_2025_273_MOESM4_ESM.zip › Fig.2/Fig2-F/KO-mdx_Merge.tif]

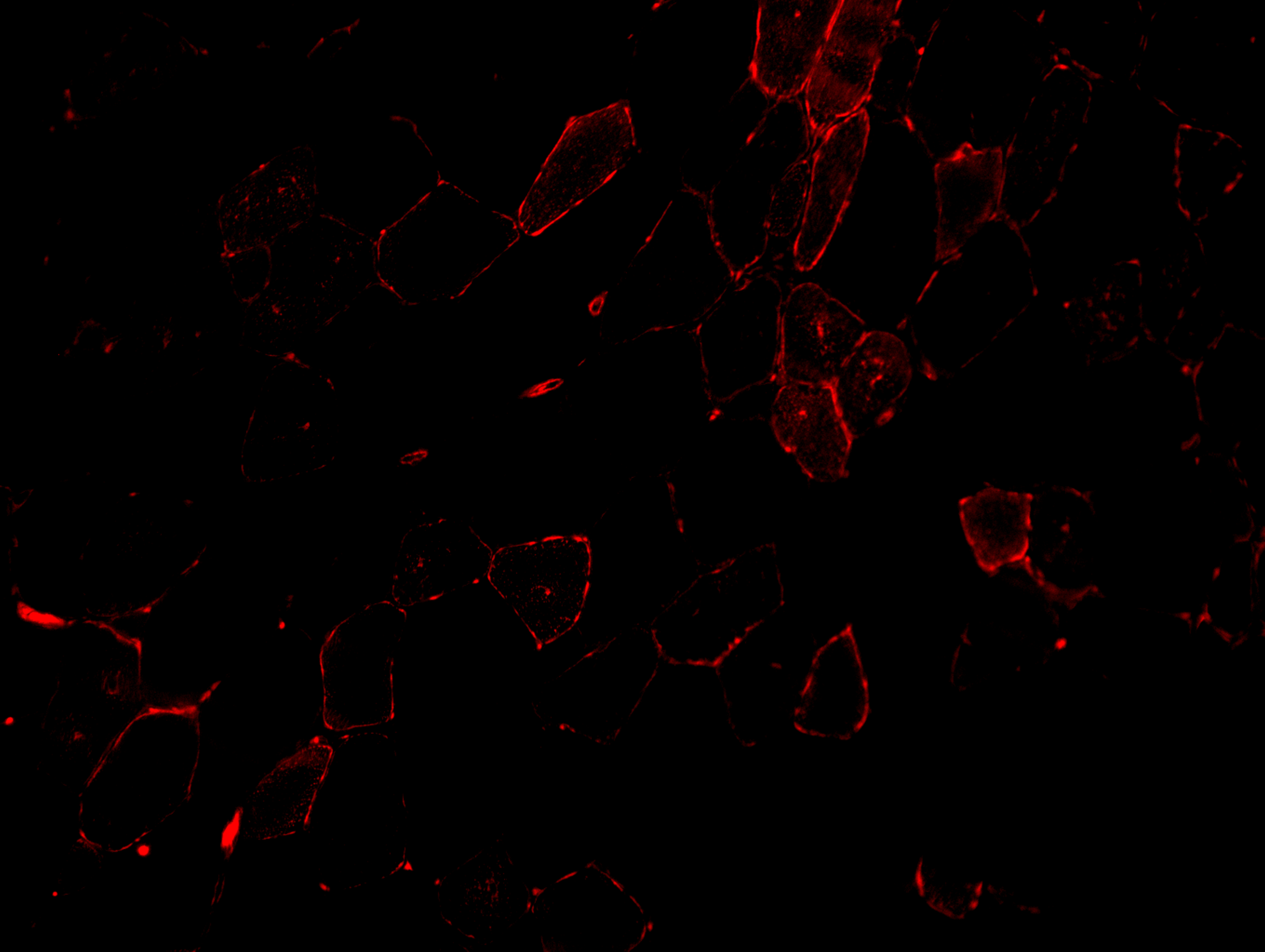

Supplement: Supplementary file 4 — Source data Fig. 2 [file 44321_2025_273_MOESM4_ESM.zip › Fig.2/Fig2-F/WT-mdx_Pax7.tif]

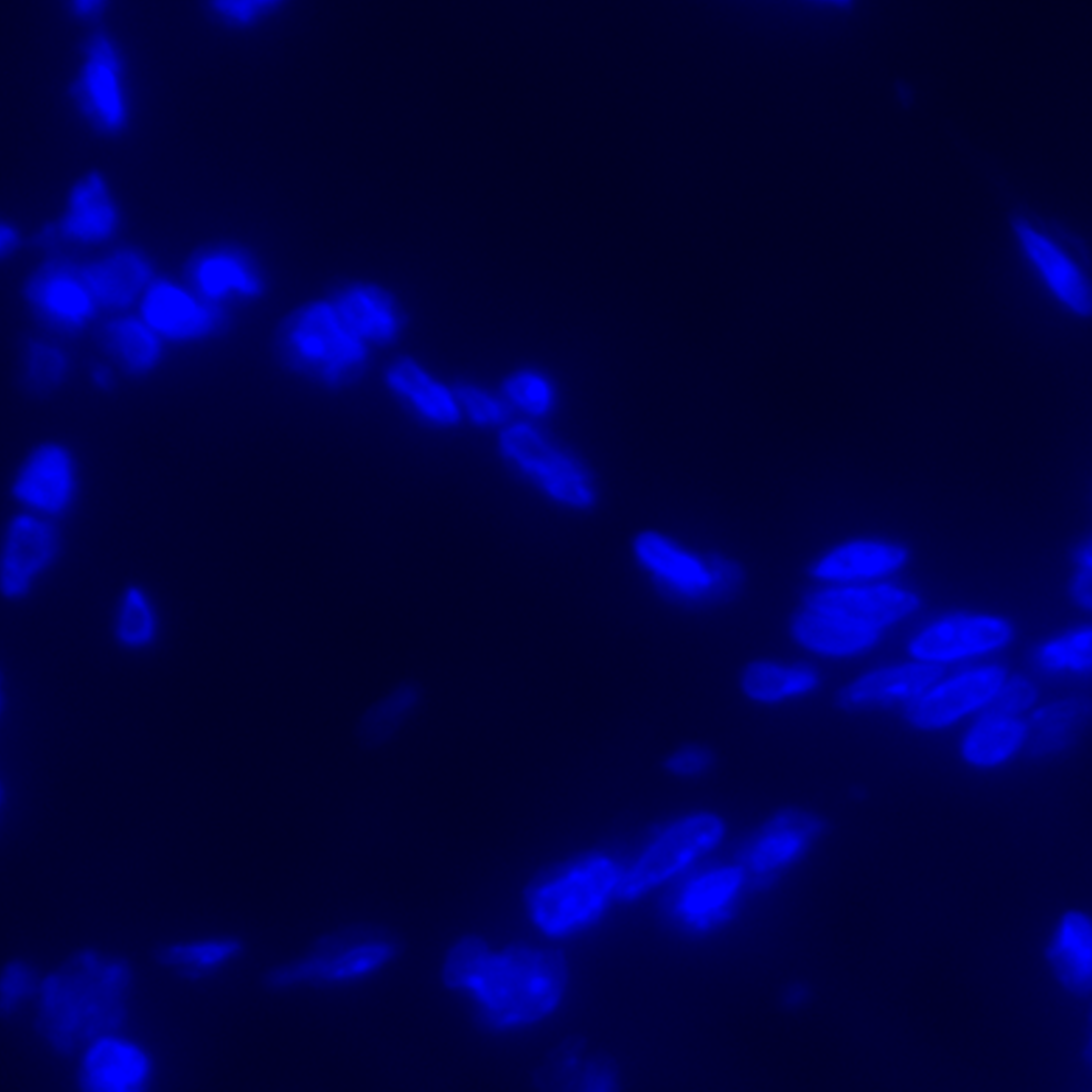

Supplement: Supplementary file 4 — Source data Fig. 2 [file 44321_2025_273_MOESM4_ESM.zip › Fig.2/Fig2-C/33KO-mdx-DAPI.tif]

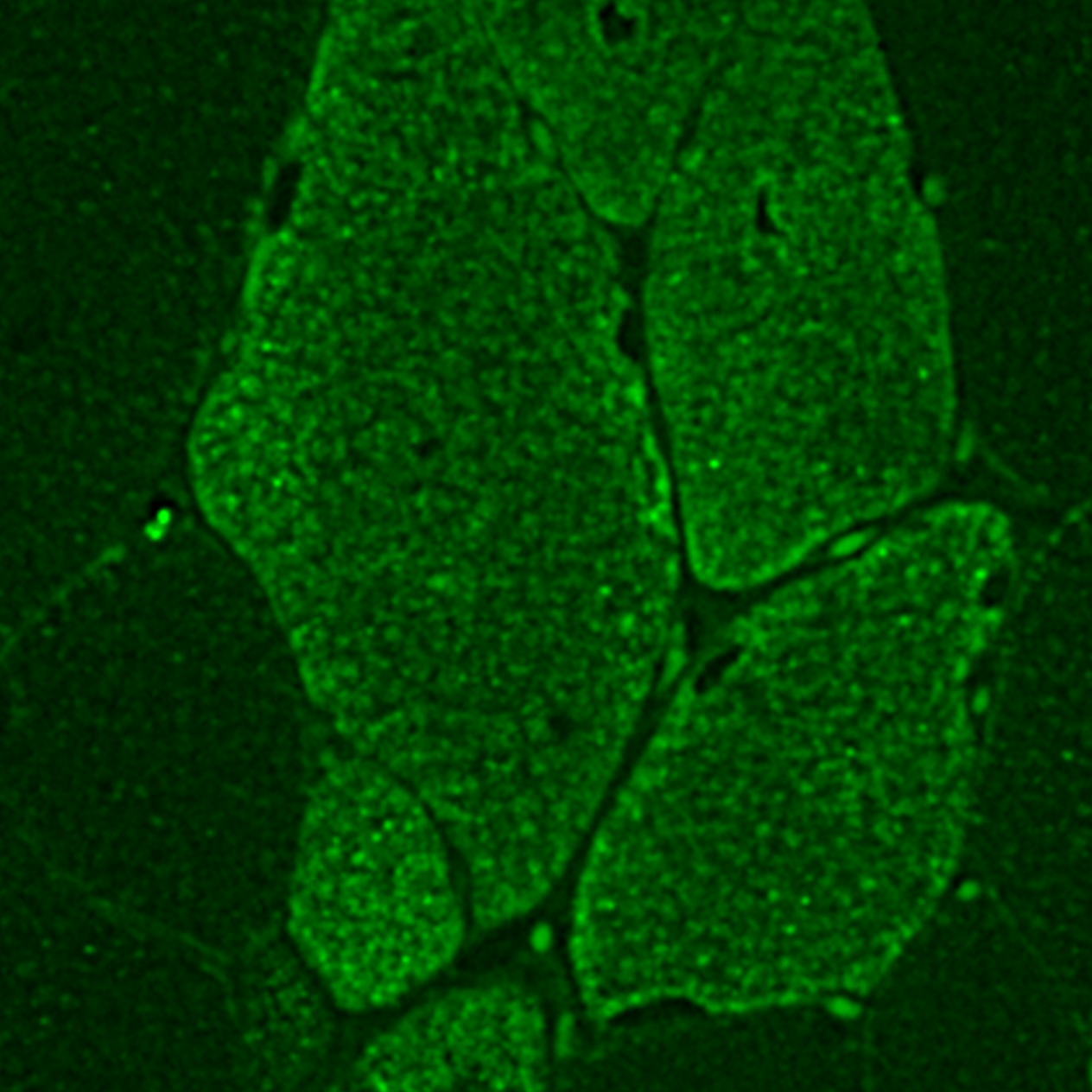

Supplement: Supplementary file 4 — Source data Fig. 2 [file 44321_2025_273_MOESM4_ESM.zip › Fig.2/Fig2-C/WT-mdx-Ki67.tif]

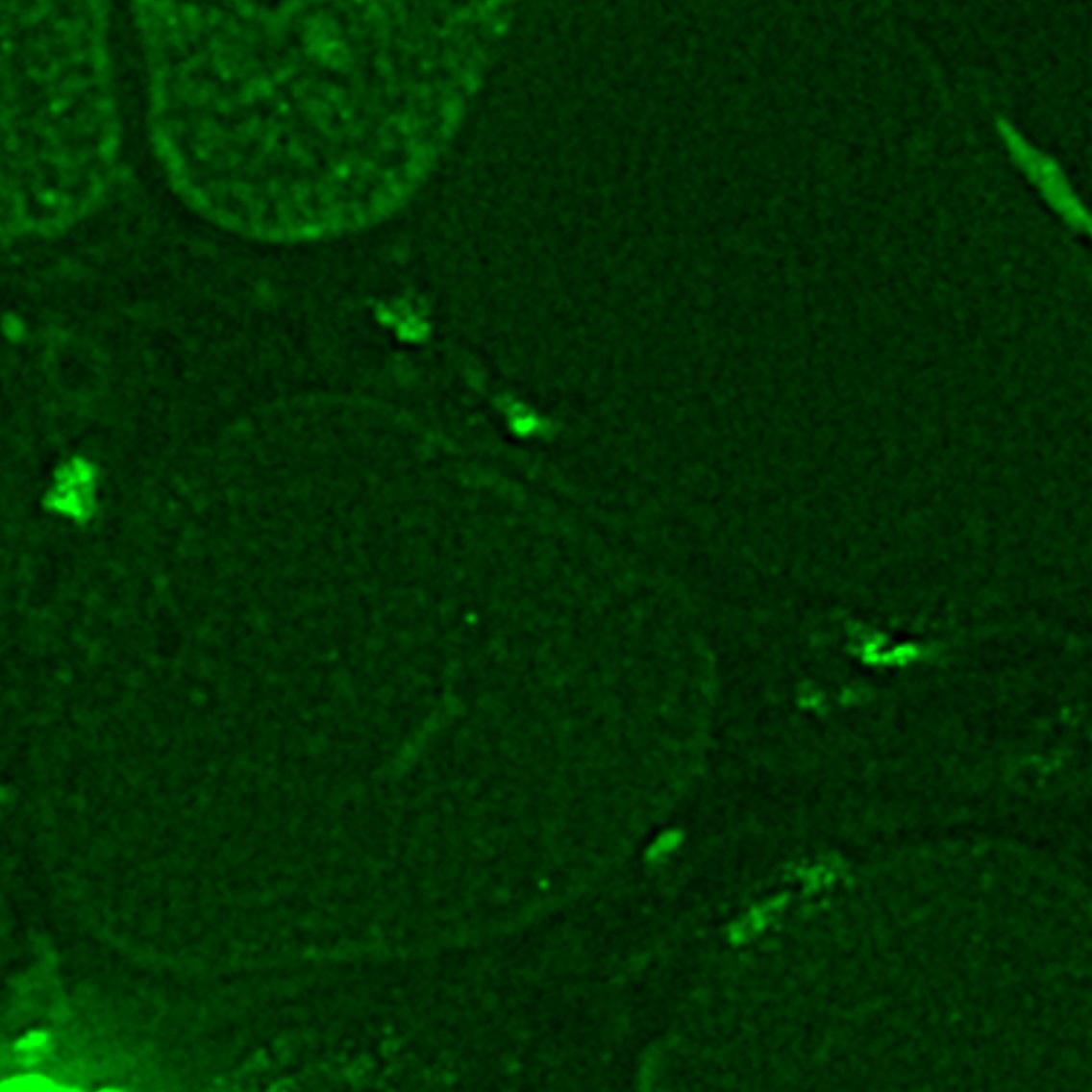

Supplement: Supplementary file 4 — Source data Fig. 2 [file 44321_2025_273_MOESM4_ESM.zip › Fig.2/Fig2-C/33KO-mdx-Ki67.tif]

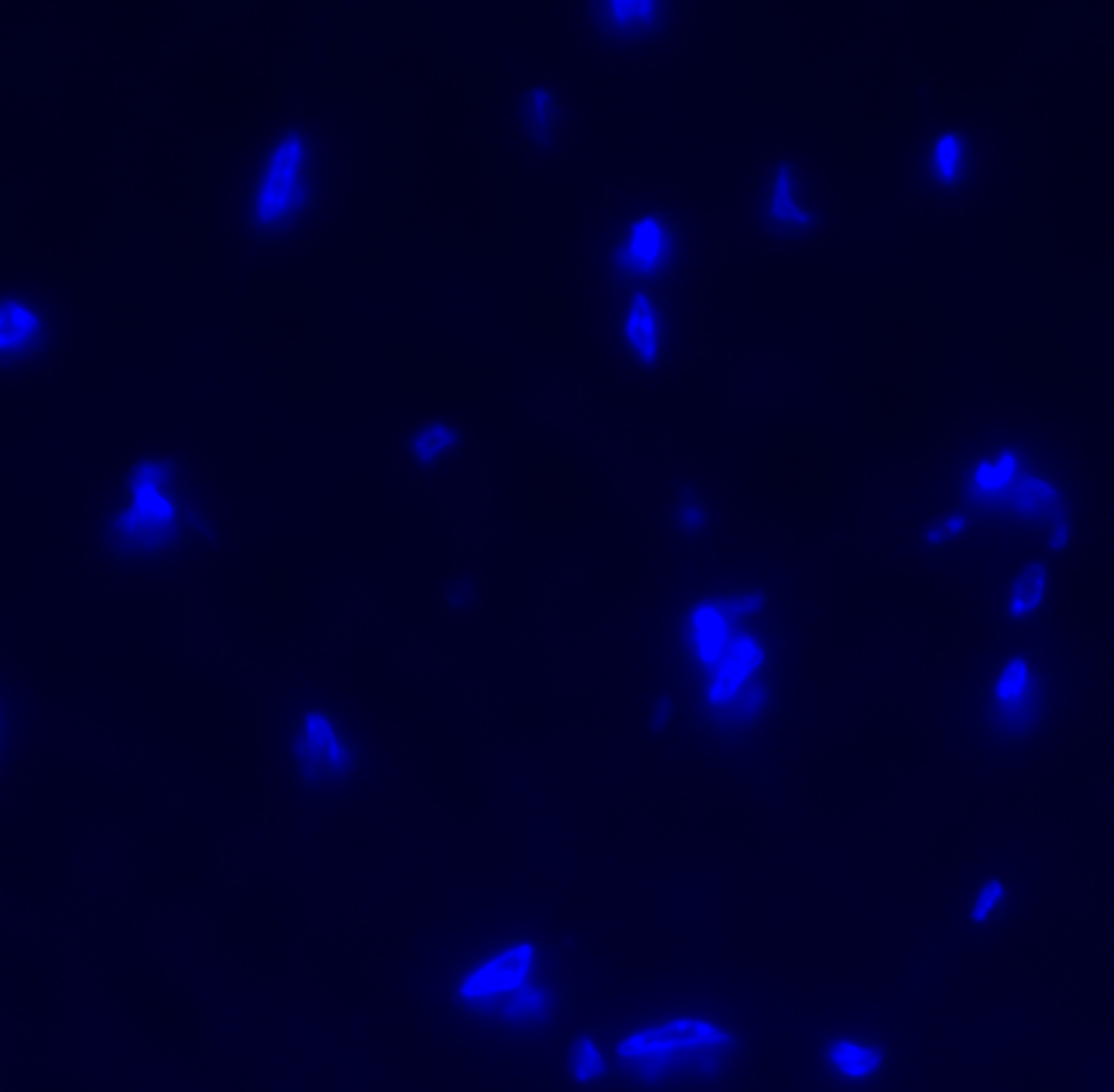

Supplement: Supplementary file 4 — Source data Fig. 2 [file 44321_2025_273_MOESM4_ESM.zip › Fig.2/Fig2-C/WT-mdx-DAPI.tif]

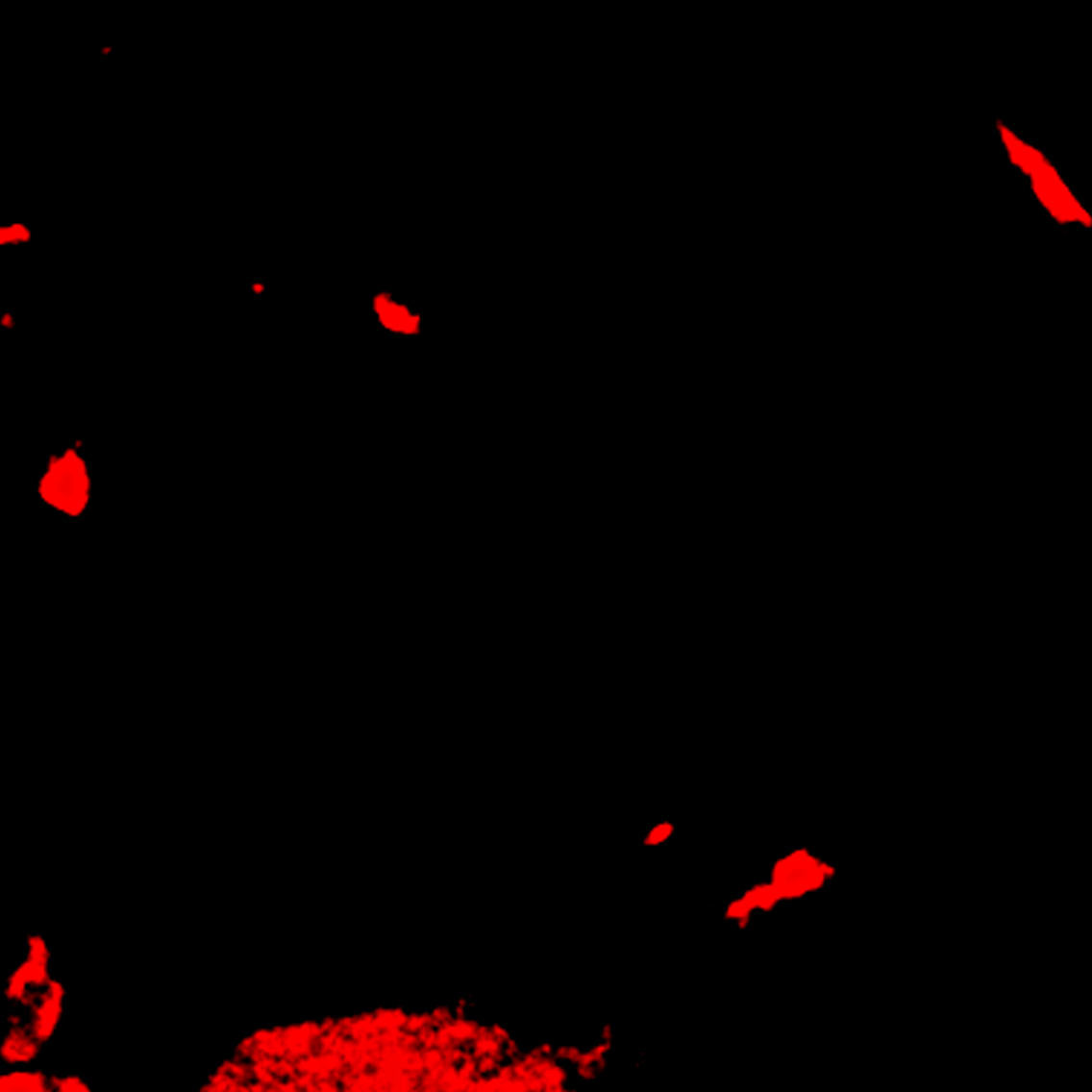

Supplement: Supplementary file 4 — Source data Fig. 2 [file 44321_2025_273_MOESM4_ESM.zip › Fig.2/Fig2-C/33KO-mdx-Pax7.tif]

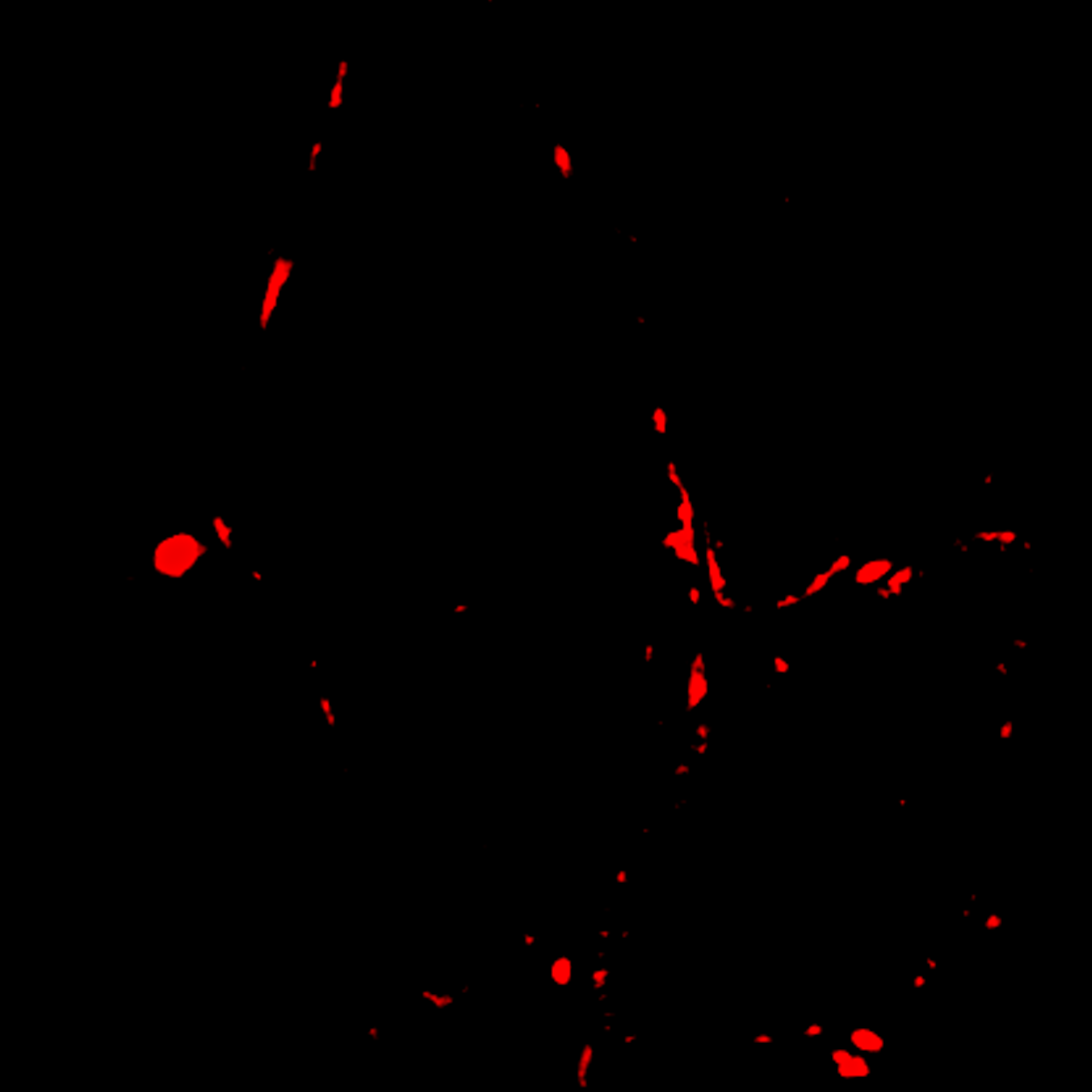

Supplement: Supplementary file 4 — Source data Fig. 2 [file 44321_2025_273_MOESM4_ESM.zip › Fig.2/Fig2-C/WT-mdx-Pax7.tif]

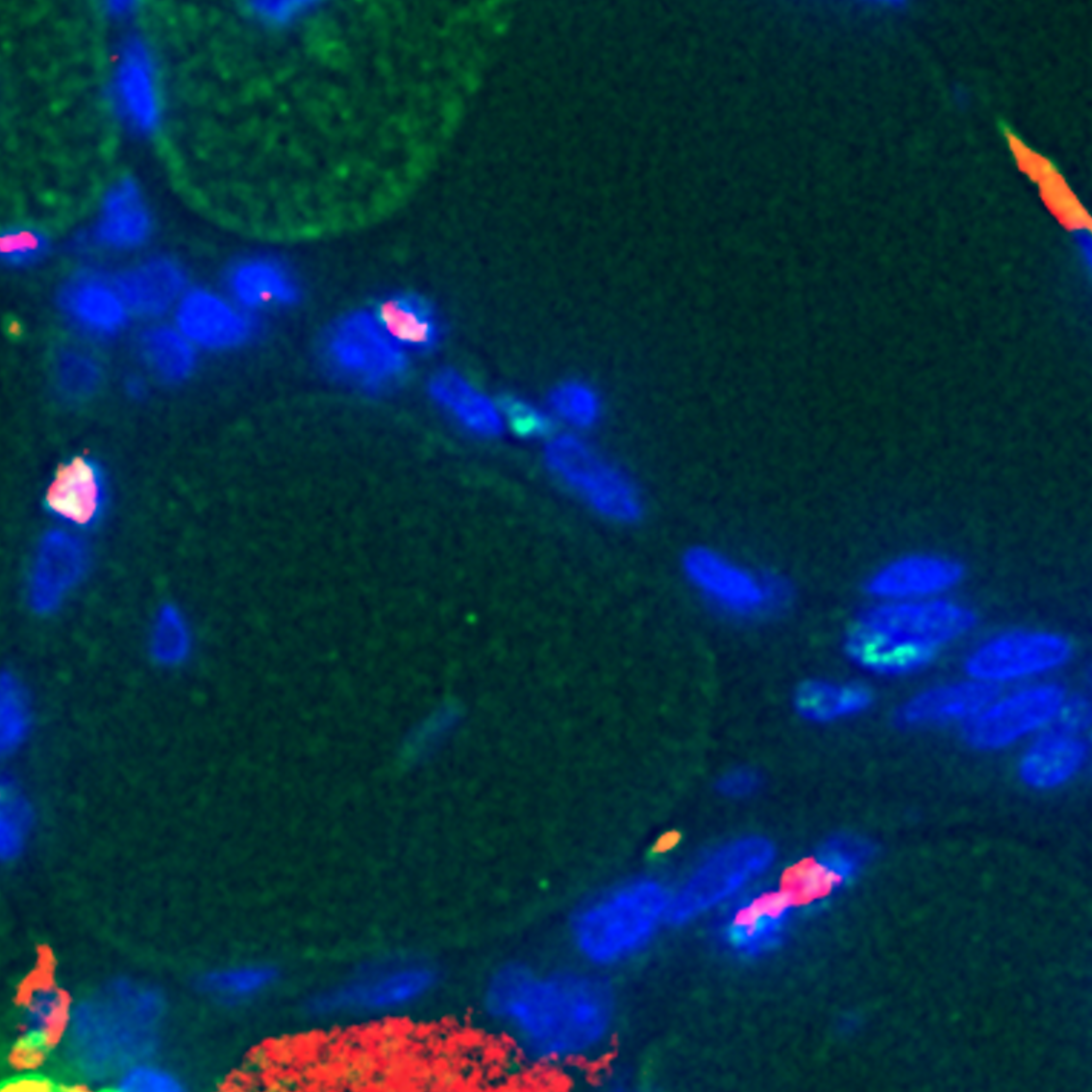

Supplement: Supplementary file 4 — Source data Fig. 2 [file 44321_2025_273_MOESM4_ESM.zip › Fig.2/Fig2-C/33KO-mdx-Merge.tif]

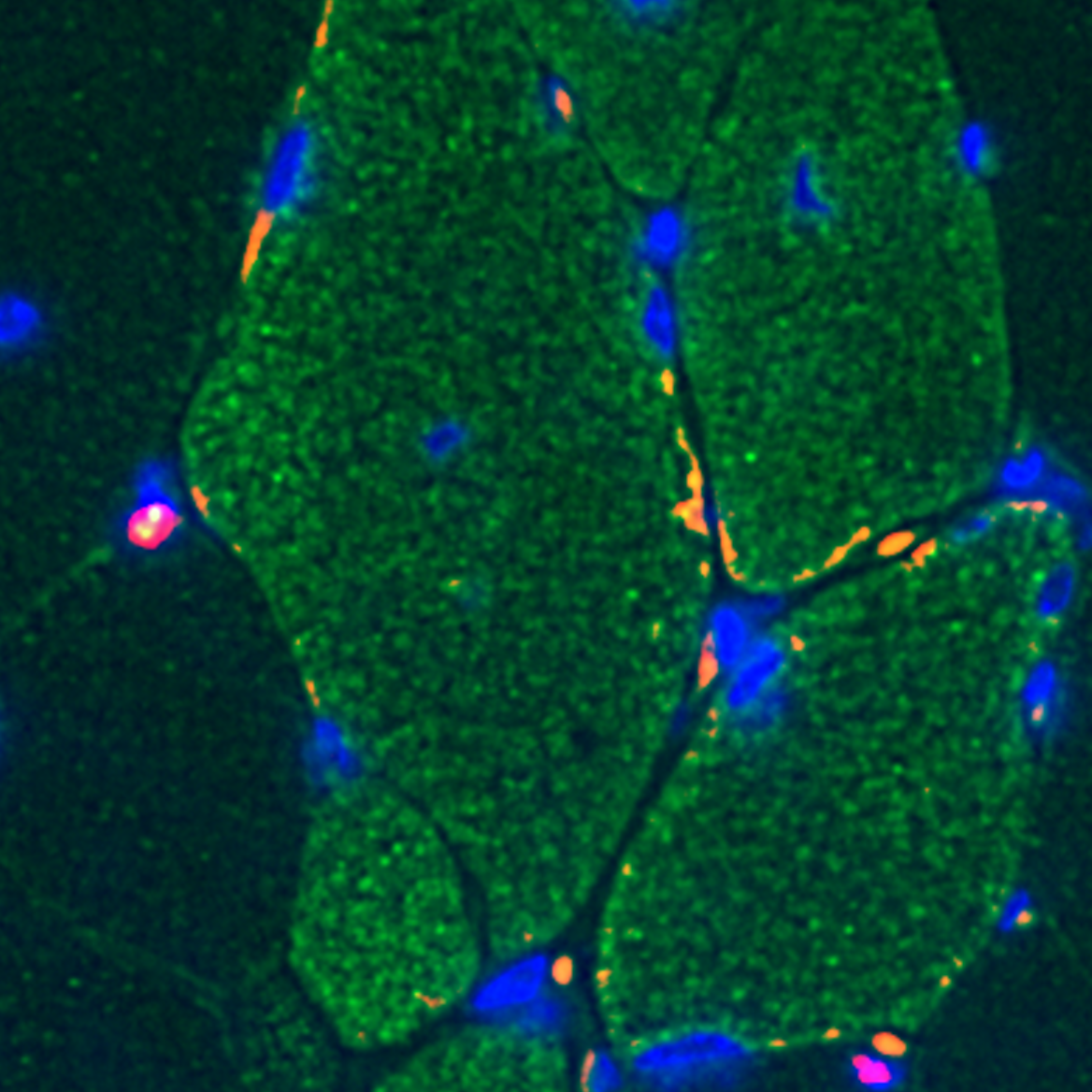

Supplement: Supplementary file 4 — Source data Fig. 2 [file 44321_2025_273_MOESM4_ESM.zip › Fig.2/Fig2-C/WT-mdx-Merge.tif]

Fig2-E

WT/*mdx*

KO/*mdx*

kDa

Utrophin

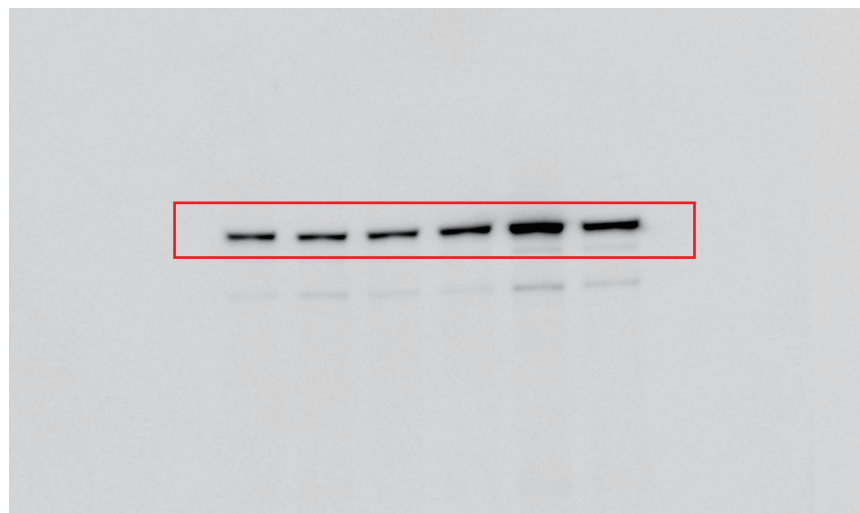

— 400

GAPDH

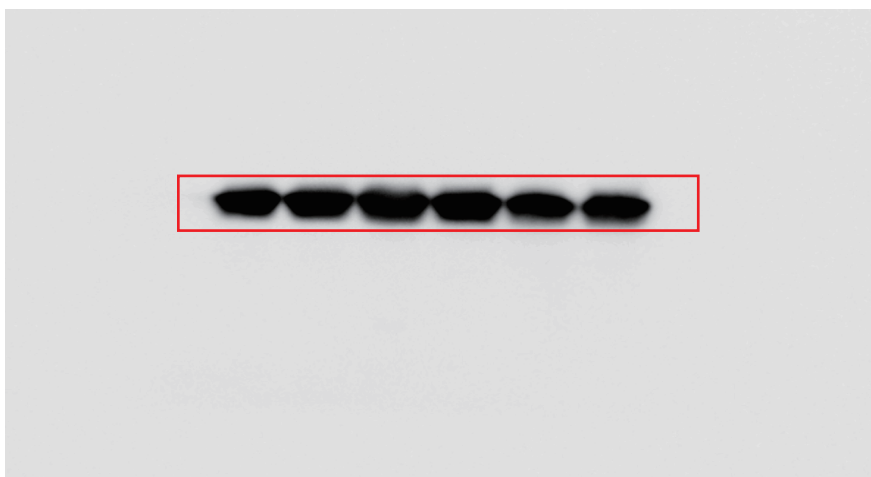

— 36

Supplement: Supplementary file 4 — Source data Fig. 2 [file 44321_2025_273_MOESM4_ESM.zip › Fig.2/Fig2-E/Fig2-E.pdf]

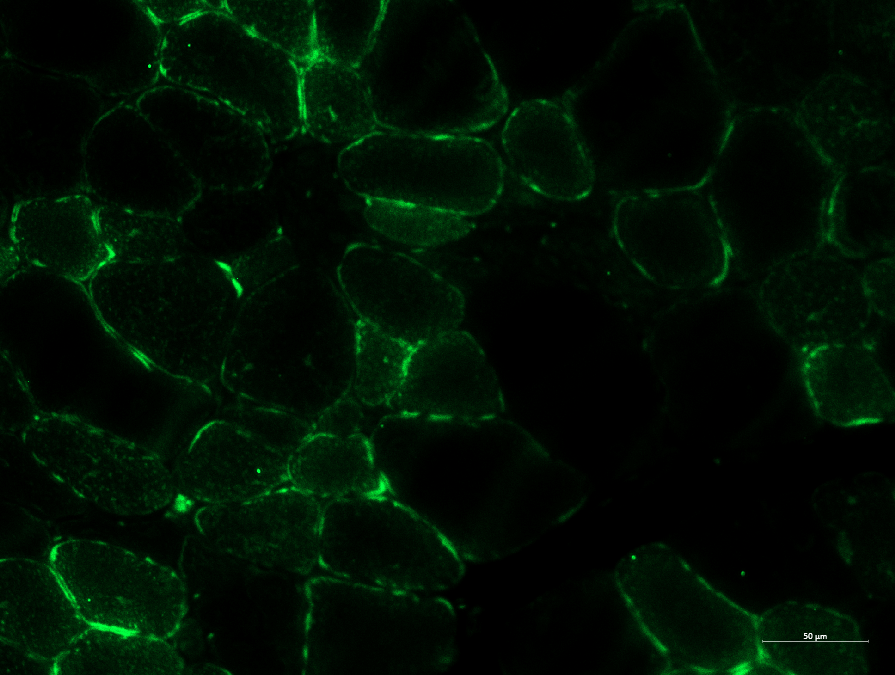

Supplement: Supplementary file 4 — Source data Fig. 2 [file 44321_2025_273_MOESM4_ESM.zip › Fig.2/Fig2-A/Fig2-A/WT-mdx-MyoD.tif]

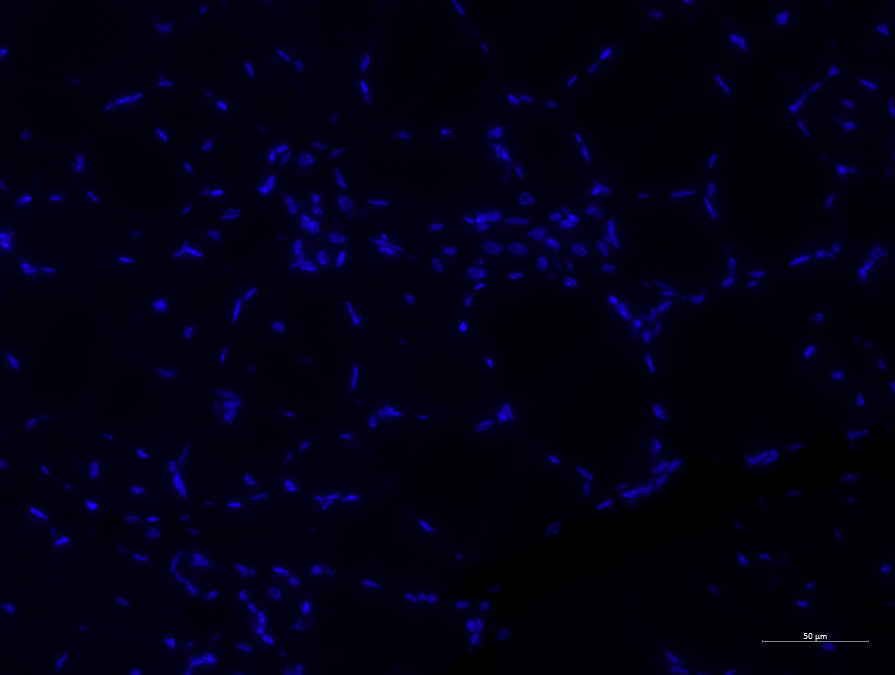

Supplement: Supplementary file 4 — Source data Fig. 2 [file 44321_2025_273_MOESM4_ESM.zip › Fig.2/Fig2-A/Fig2-A/WT-mdx-DAPI.tif]

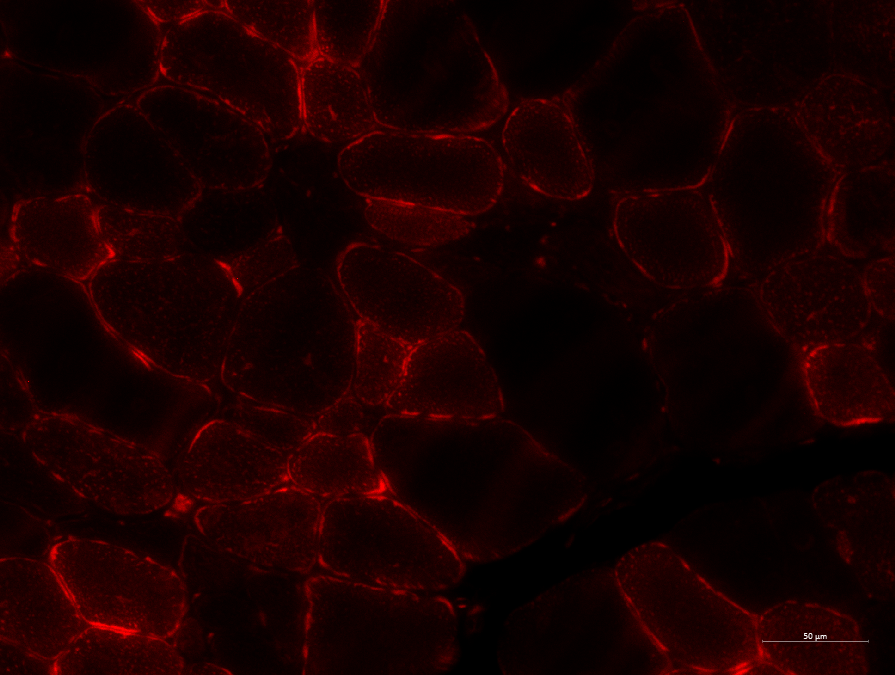

Supplement: Supplementary file 4 — Source data Fig. 2 [file 44321_2025_273_MOESM4_ESM.zip › Fig.2/Fig2-A/Fig2-A/WT-mdx-pax7.tif]

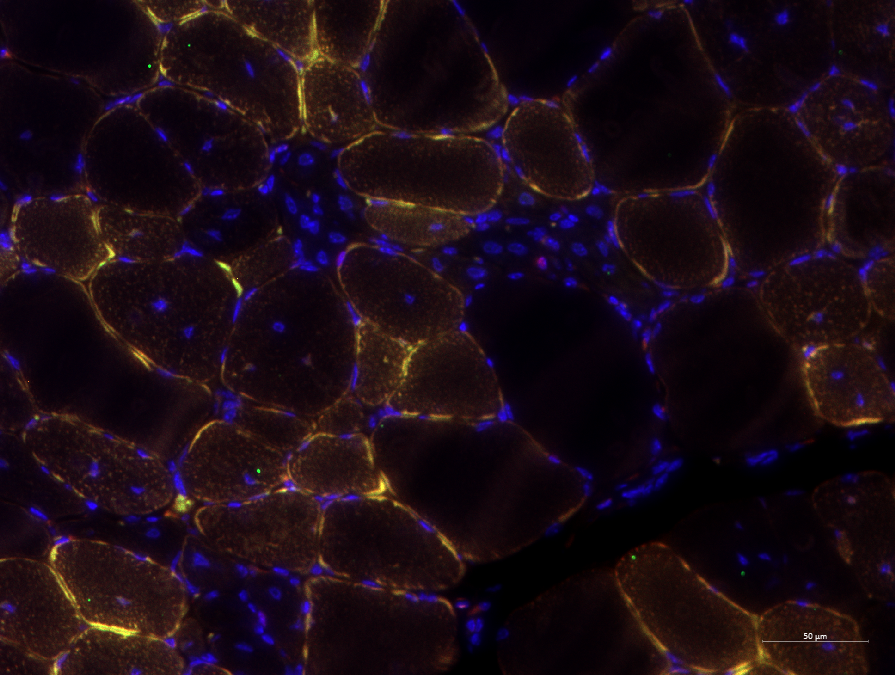

Supplement: Supplementary file 4 — Source data Fig. 2 [file 44321_2025_273_MOESM4_ESM.zip › Fig.2/Fig2-A/Fig2-A/WT-mdx-Merge.tif]

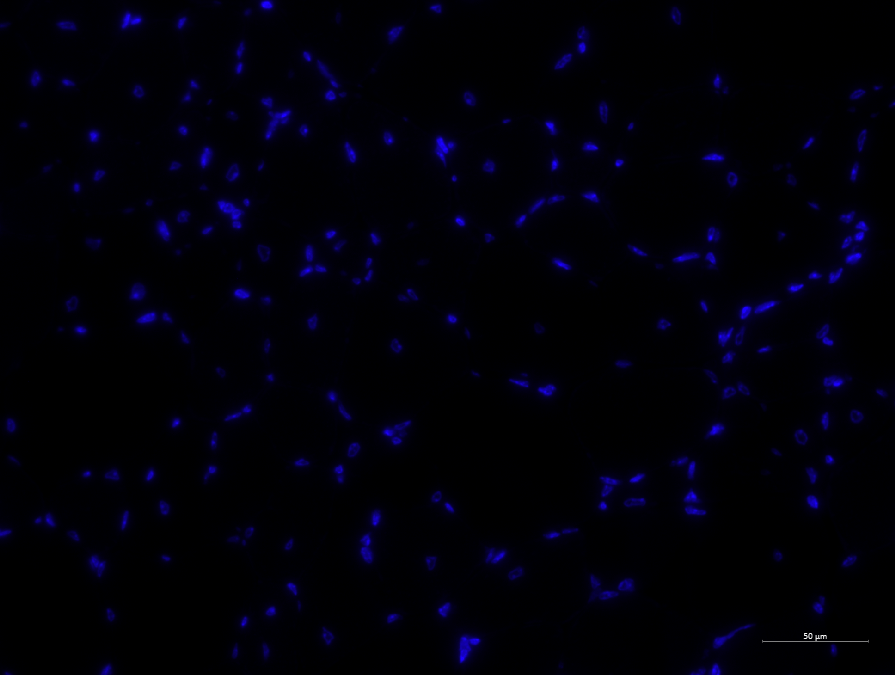

Supplement: Supplementary file 4 — Source data Fig. 2 [file 44321_2025_273_MOESM4_ESM.zip › Fig.2/Fig2-A/Fig2-A/KO-mdx-DAPI.tif]

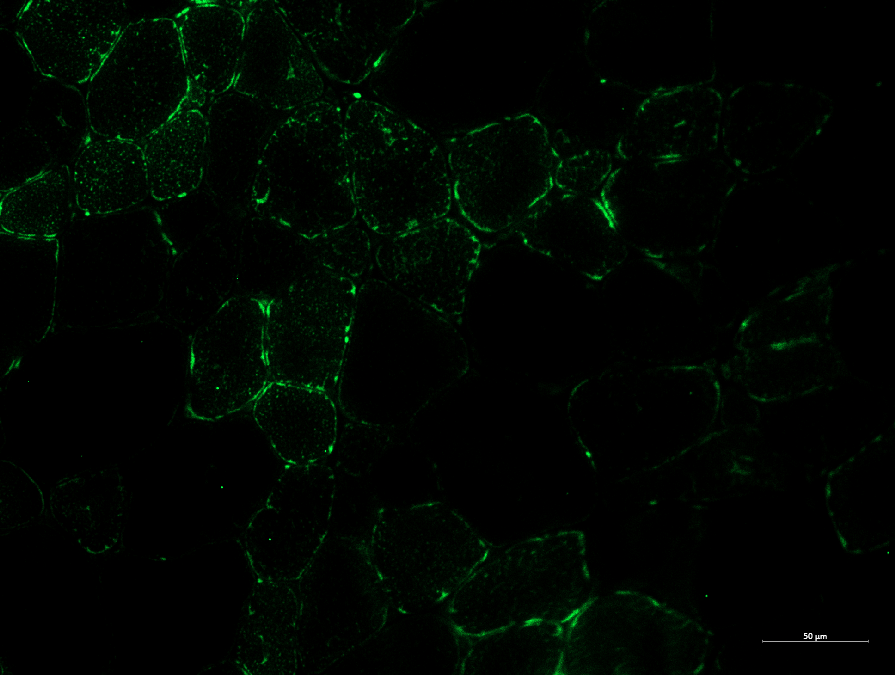

Supplement: Supplementary file 4 — Source data Fig. 2 [file 44321_2025_273_MOESM4_ESM.zip › Fig.2/Fig2-A/Fig2-A/KO-mdx-MyoD.tif]

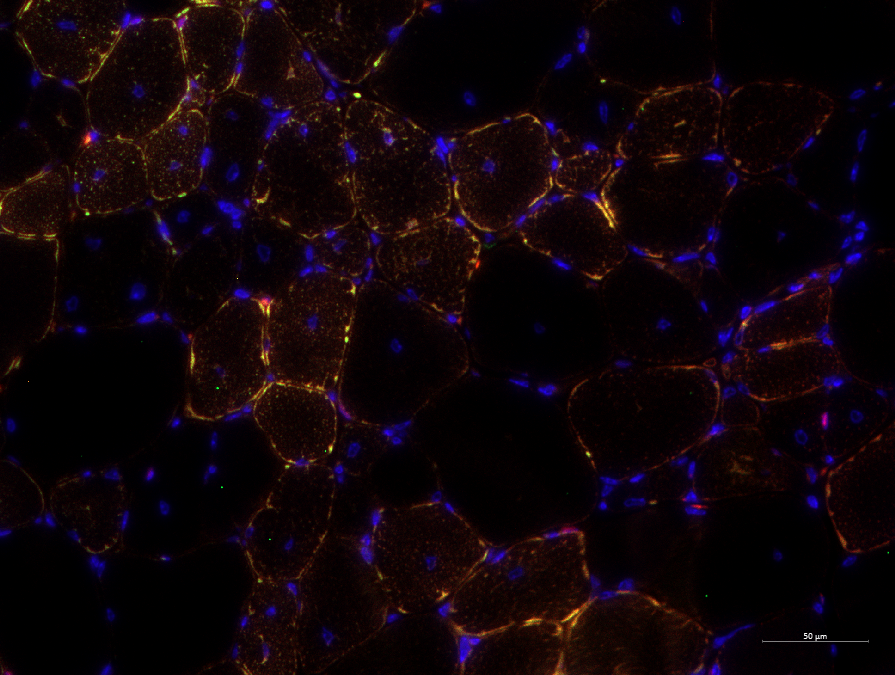

Supplement: Supplementary file 4 — Source data Fig. 2 [file 44321_2025_273_MOESM4_ESM.zip › Fig.2/Fig2-A/Fig2-A/KO-mdx-Merge.tif]

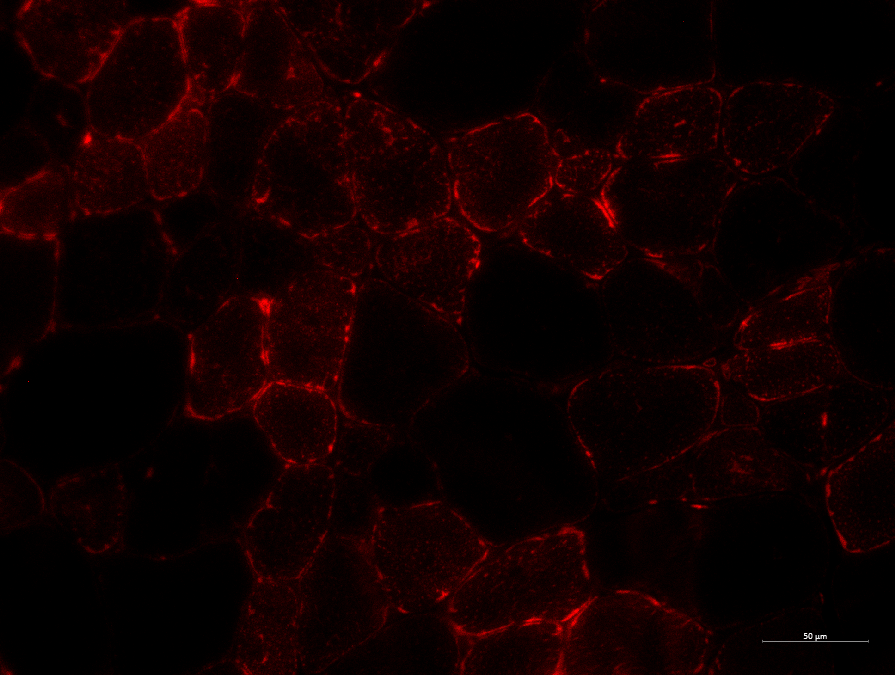

Supplement: Supplementary file 4 — Source data Fig. 2 [file 44321_2025_273_MOESM4_ESM.zip › Fig.2/Fig2-A/Fig2-A/KO-mdx-pax7.tif]

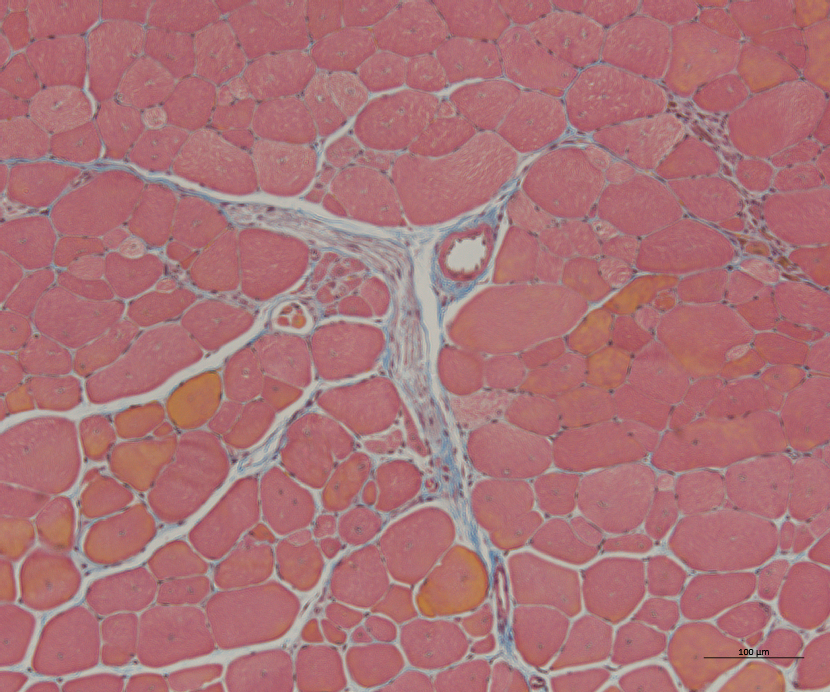

Supplement: Supplementary file 5 — Source data Fig. 3 [file 44321_2025_273_MOESM5_ESM.zip › Fig.3/Fig3-C/33bKI-mdx-3665-211-10-2.tif]

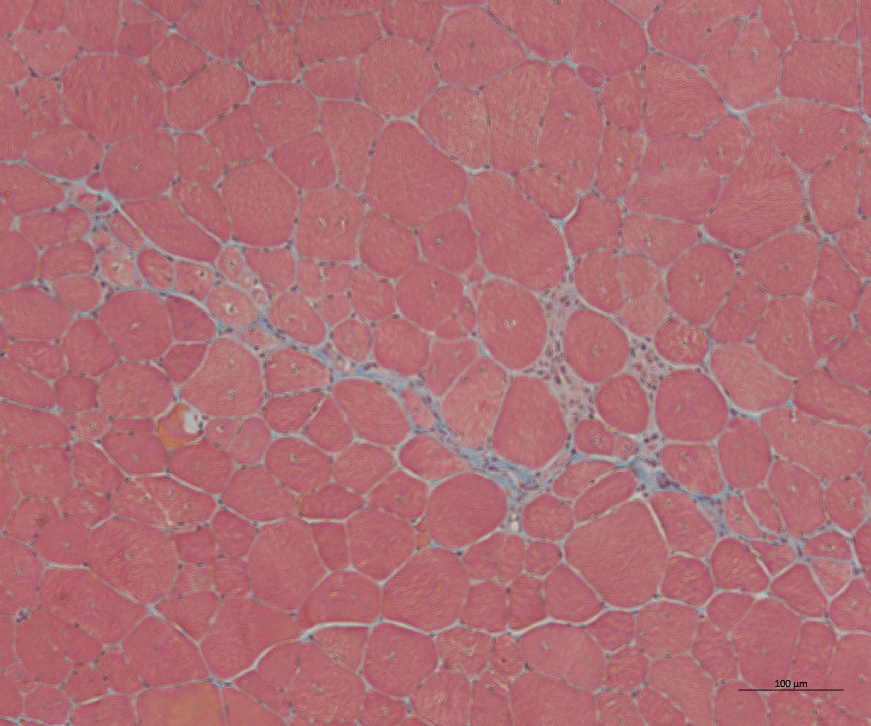

Supplement: Supplementary file 5 — Source data Fig. 3 [file 44321_2025_273_MOESM5_ESM.zip › Fig.3/Fig3-C/33bWT-mdx-3655-207-10-3.tif]

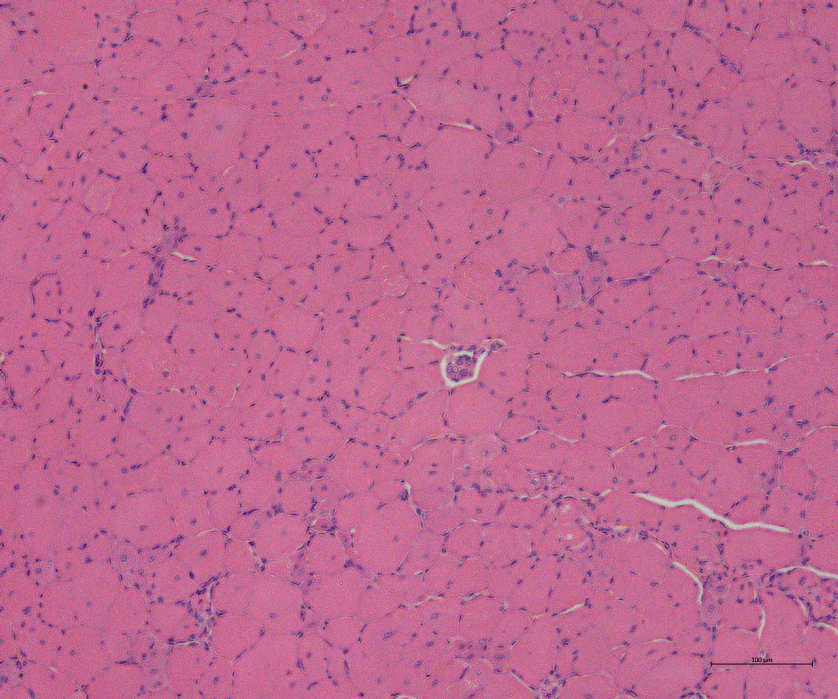

Supplement: Supplementary file 5 — Source data Fig. 3 [file 44321_2025_273_MOESM5_ESM.zip › Fig.3/Fig3-A/miR-33b-KI-mdx.tif]

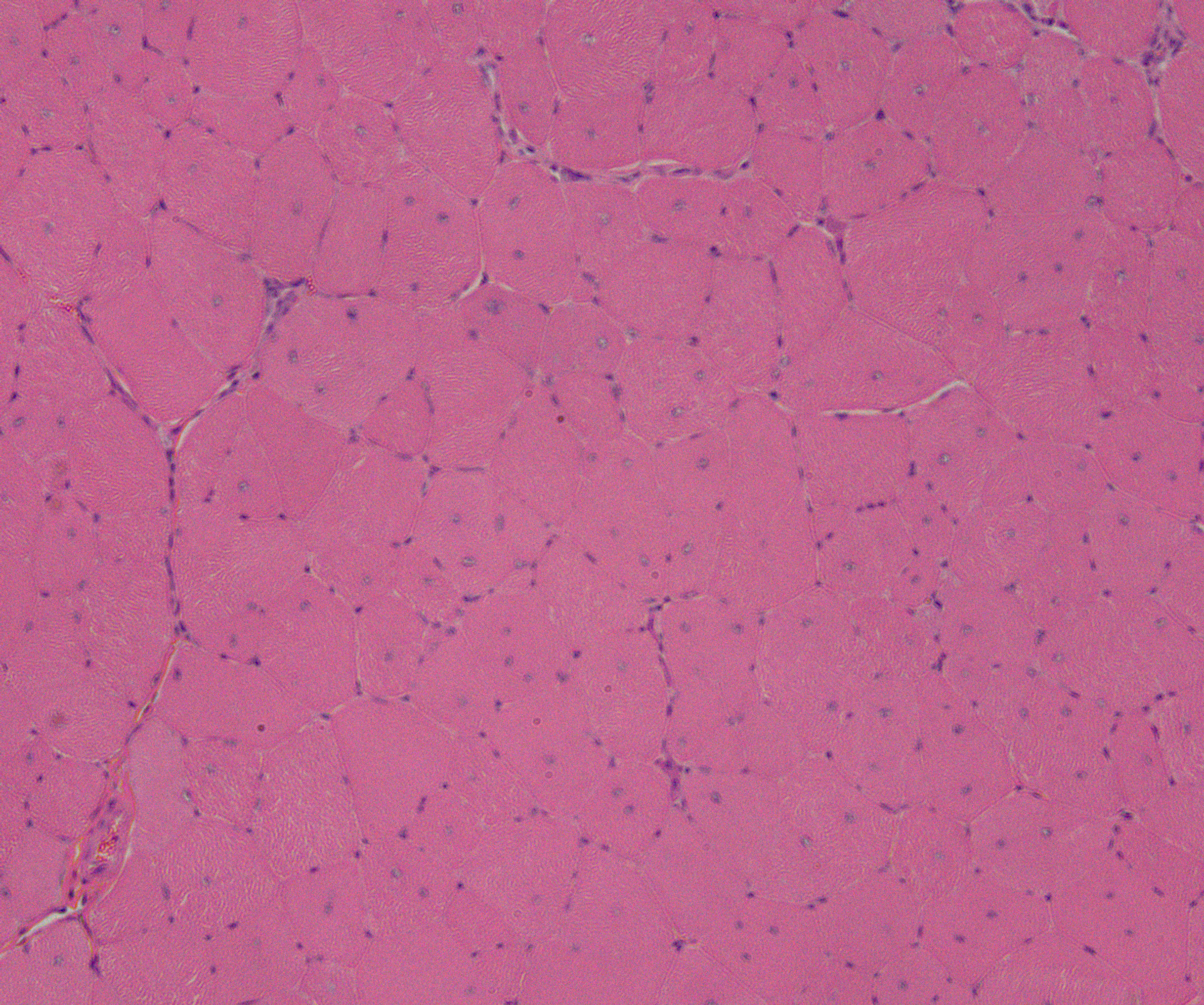

Supplement: Supplementary file 5 — Source data Fig. 3 [file 44321_2025_273_MOESM5_ESM.zip › Fig.3/Fig3-A/WT-mdx.tif]

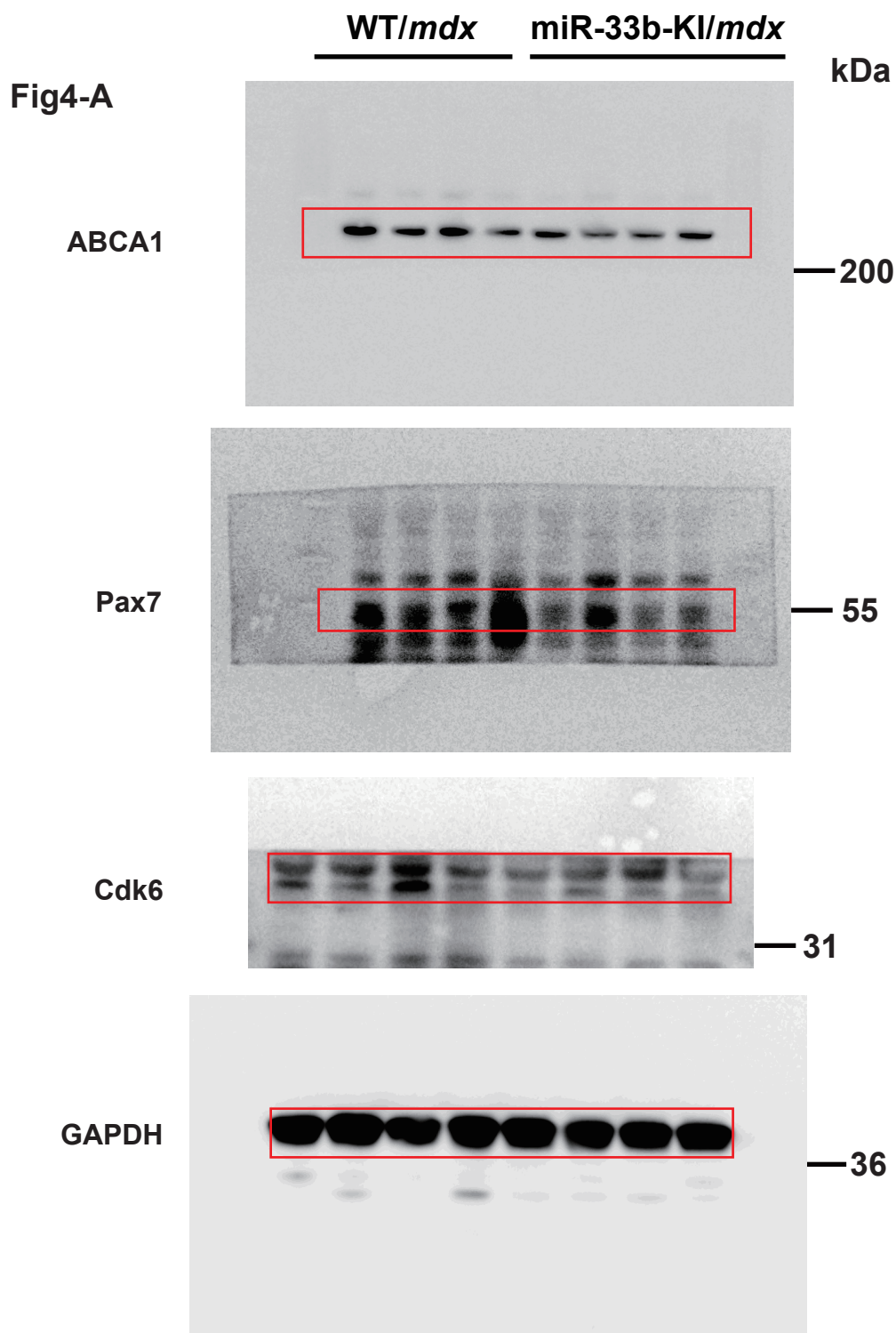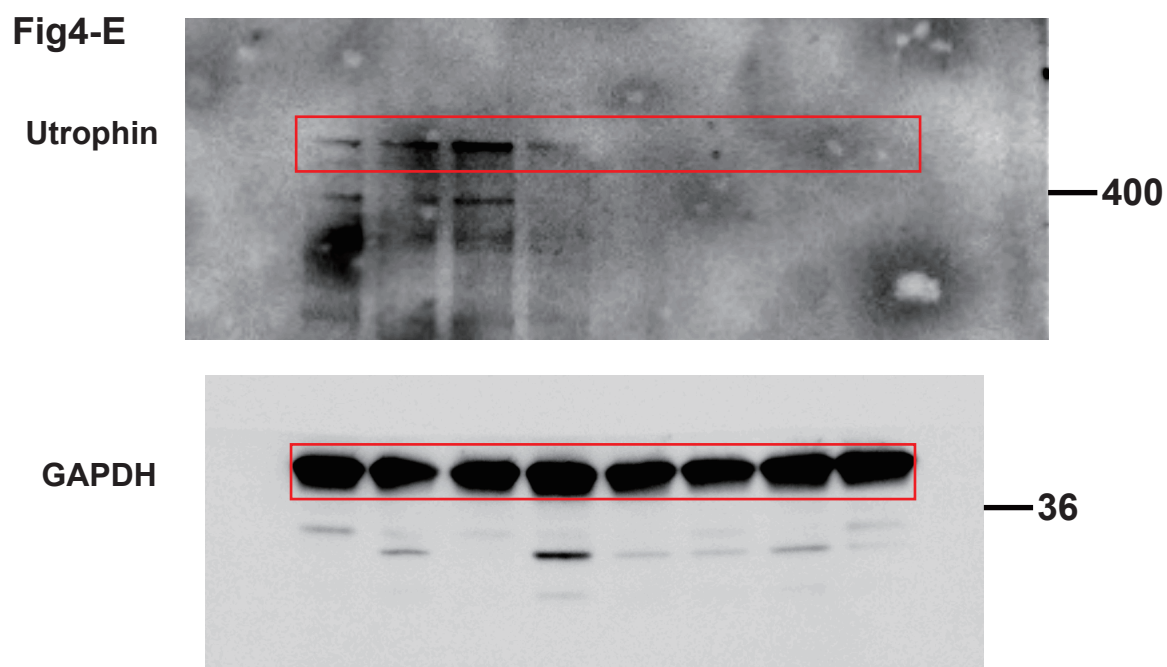

Supplement: Supplementary file 6 — Source data Fig. 4 [file 44321_2025_273_MOESM6_ESM.zip › Fig.4/Fig4-A-E/Fig4-A-E.pdf]

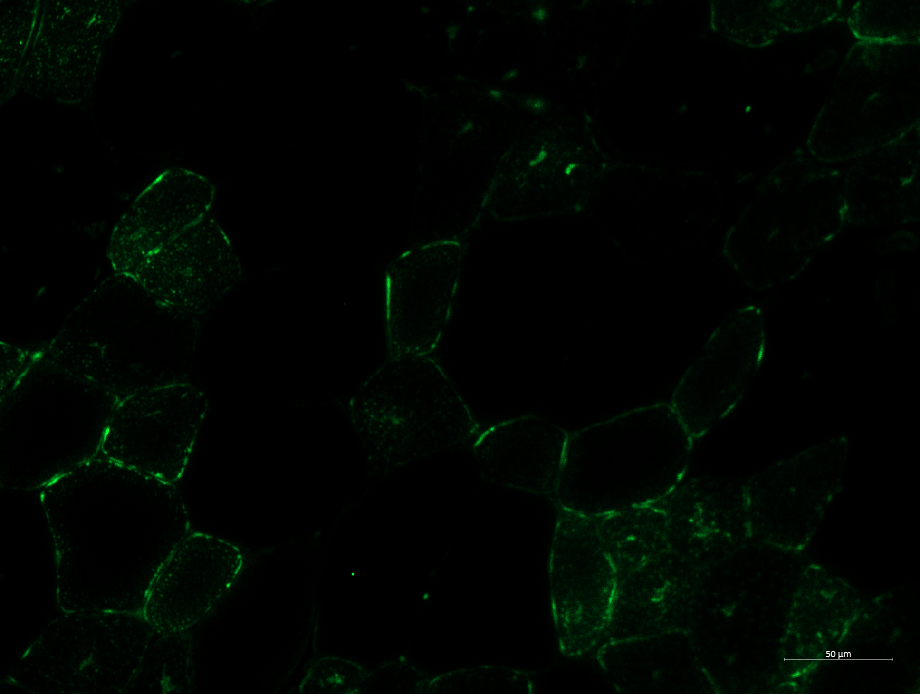

Supplement: Supplementary file 6 — Source data Fig. 4 [file 44321_2025_273_MOESM6_ESM.zip › Fig.4/Fig4-C/WT-mdx-MyoD.tif]

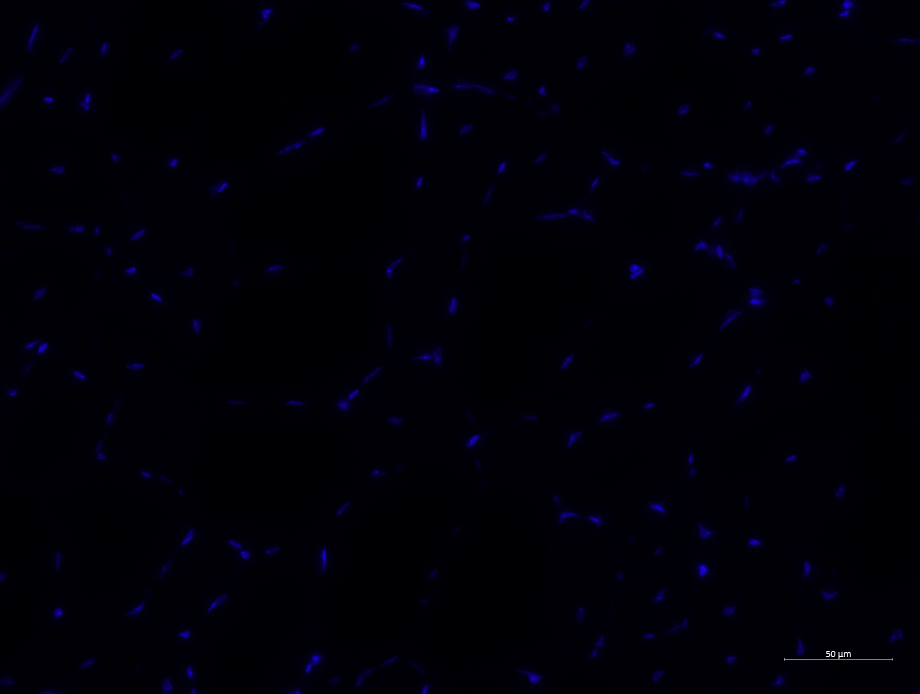

Supplement: Supplementary file 6 — Source data Fig. 4 [file 44321_2025_273_MOESM6_ESM.zip › Fig.4/Fig4-C/WT-mdx-DAPI.tif]

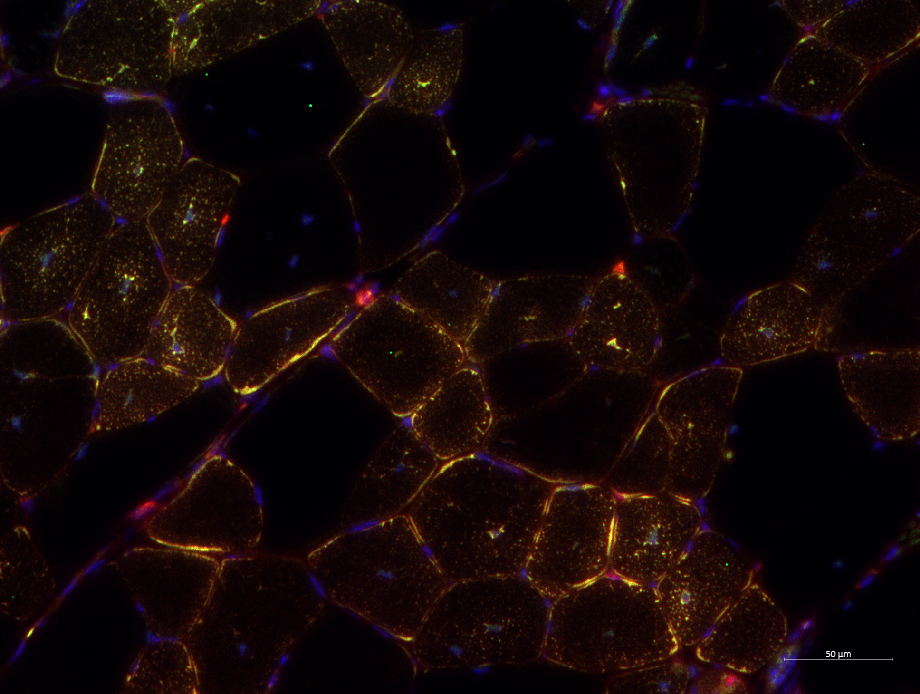

Supplement: Supplementary file 6 — Source data Fig. 4 [file 44321_2025_273_MOESM6_ESM.zip › Fig.4/Fig4-C/miR-33b-KI-mdx-Merge.tif]

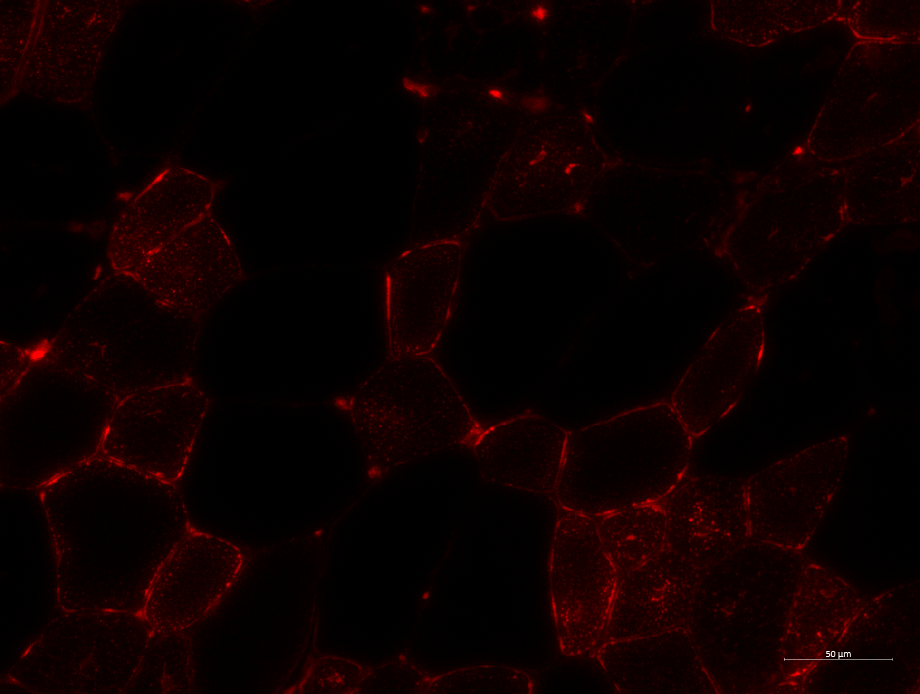

Supplement: Supplementary file 6 — Source data Fig. 4 [file 44321_2025_273_MOESM6_ESM.zip › Fig.4/Fig4-C/WT-mdx-Pax7.tif]

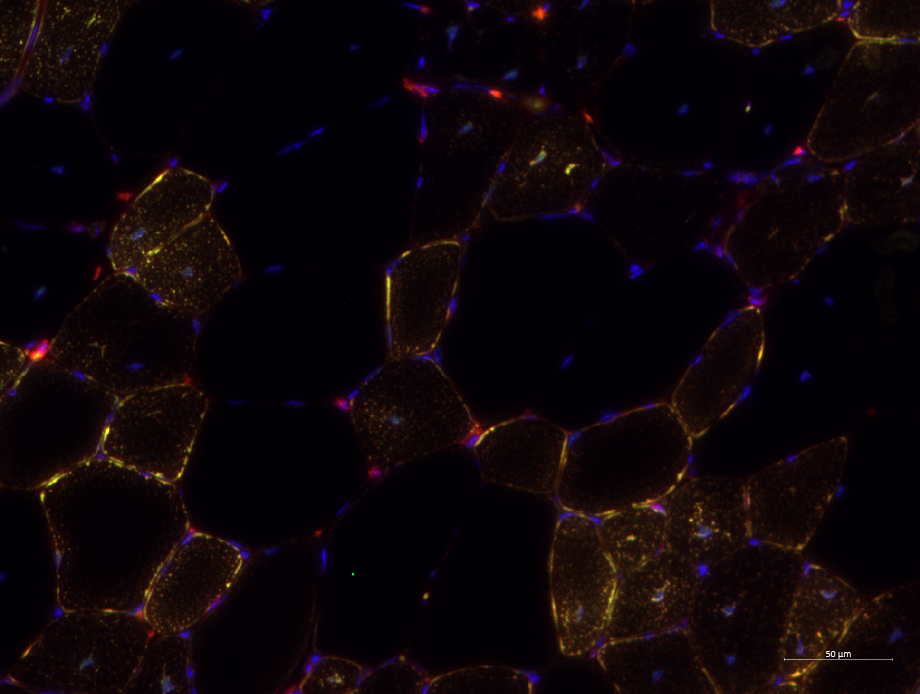

Supplement: Supplementary file 6 — Source data Fig. 4 [file 44321_2025_273_MOESM6_ESM.zip › Fig.4/Fig4-C/WT-mdx-Merge.tif]

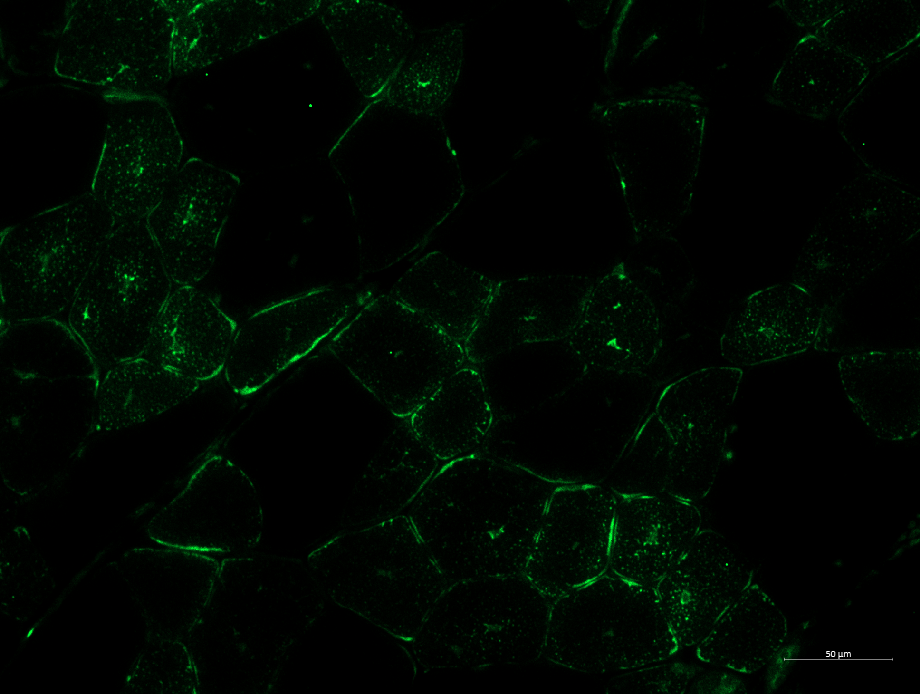

Supplement: Supplementary file 6 — Source data Fig. 4 [file 44321_2025_273_MOESM6_ESM.zip › Fig.4/Fig4-C/miR-33b-KI-mdx-MyoD.tif]

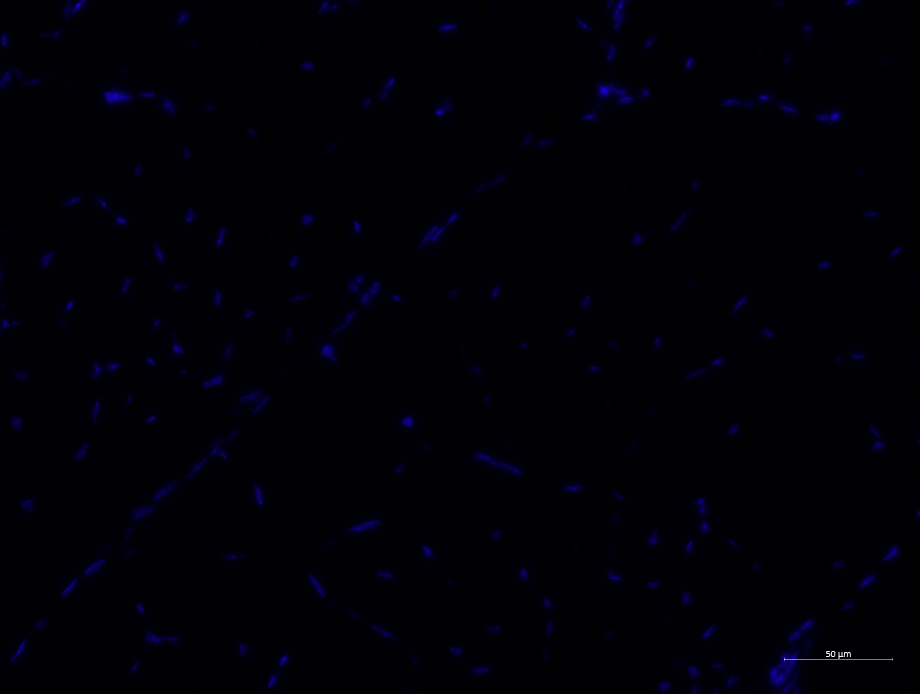

Supplement: Supplementary file 6 — Source data Fig. 4 [file 44321_2025_273_MOESM6_ESM.zip › Fig.4/Fig4-C/miR-33b-KI-mdx-DAPI.tif]

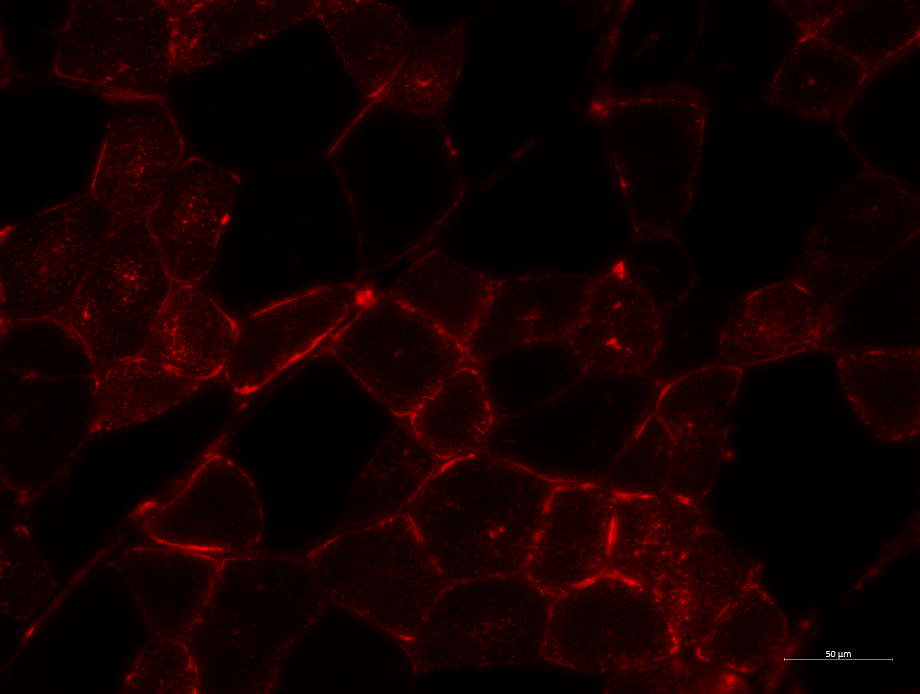

Supplement: Supplementary file 6 — Source data Fig. 4 [file 44321_2025_273_MOESM6_ESM.zip › Fig.4/Fig4-C/miR-33b-KI-mdx-Pax7.tif]

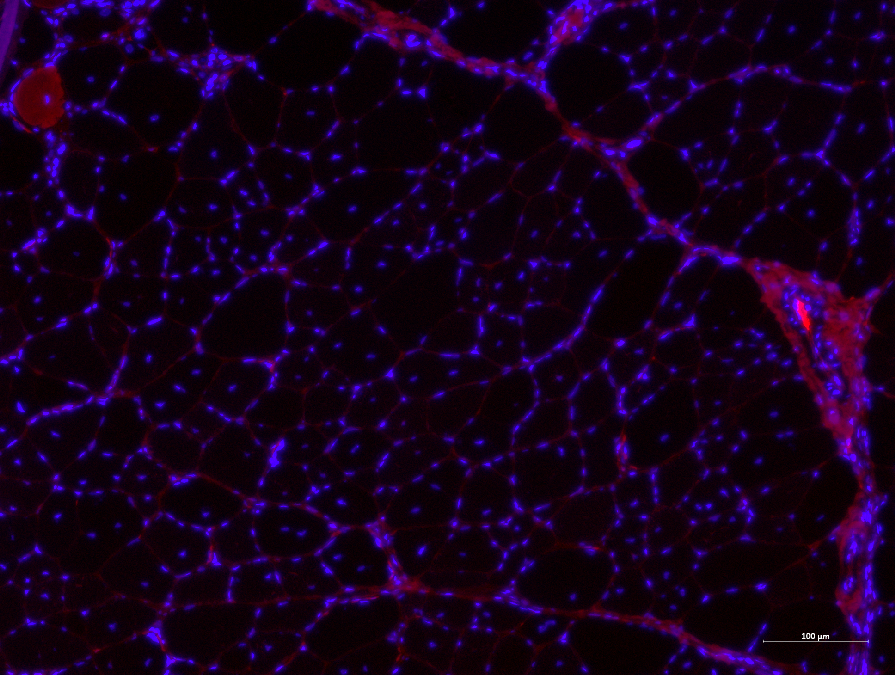

Supplement: Supplementary file 6 — Source data Fig. 4 [file 44321_2025_273_MOESM6_ESM.zip › Fig.4/Fig4-F/miR-33bKI-mdx-Merge.tif]

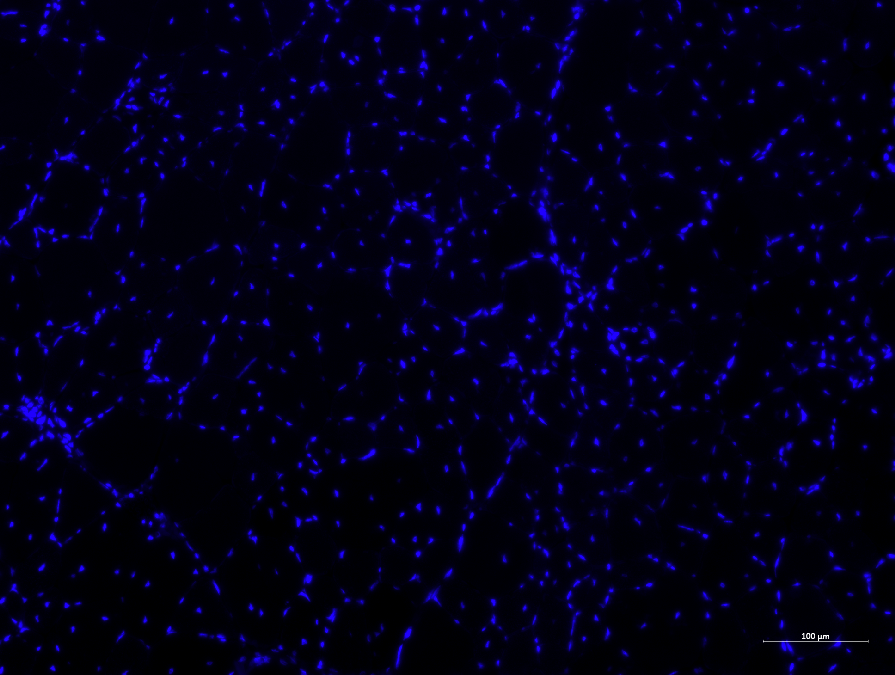

Supplement: Supplementary file 6 — Source data Fig. 4 [file 44321_2025_273_MOESM6_ESM.zip › Fig.4/Fig4-F/WT-mdx-DAPI.tif]

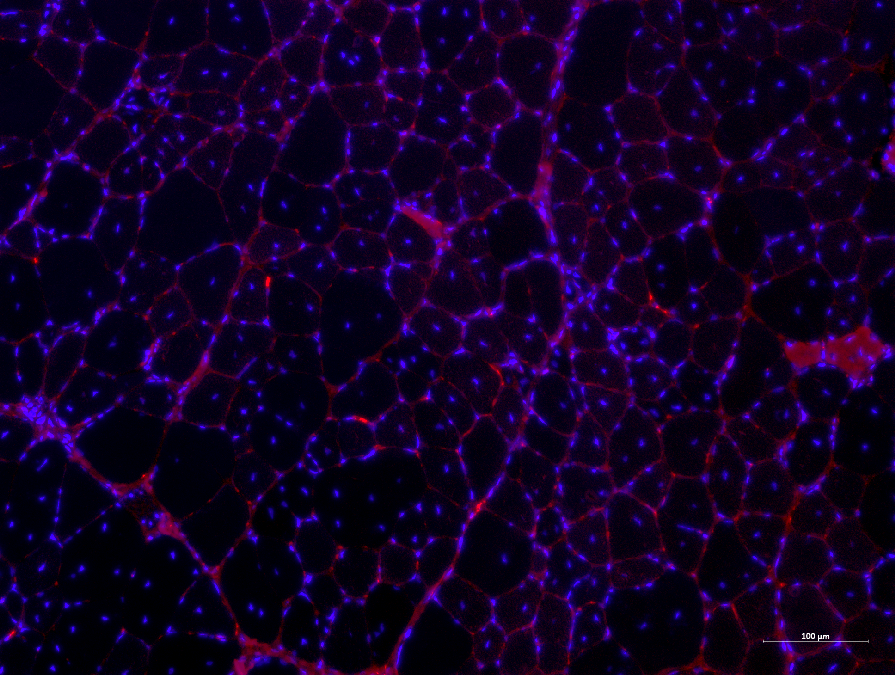

Supplement: Supplementary file 6 — Source data Fig. 4 [file 44321_2025_273_MOESM6_ESM.zip › Fig.4/Fig4-F/WT-mdx-Merge.tif]

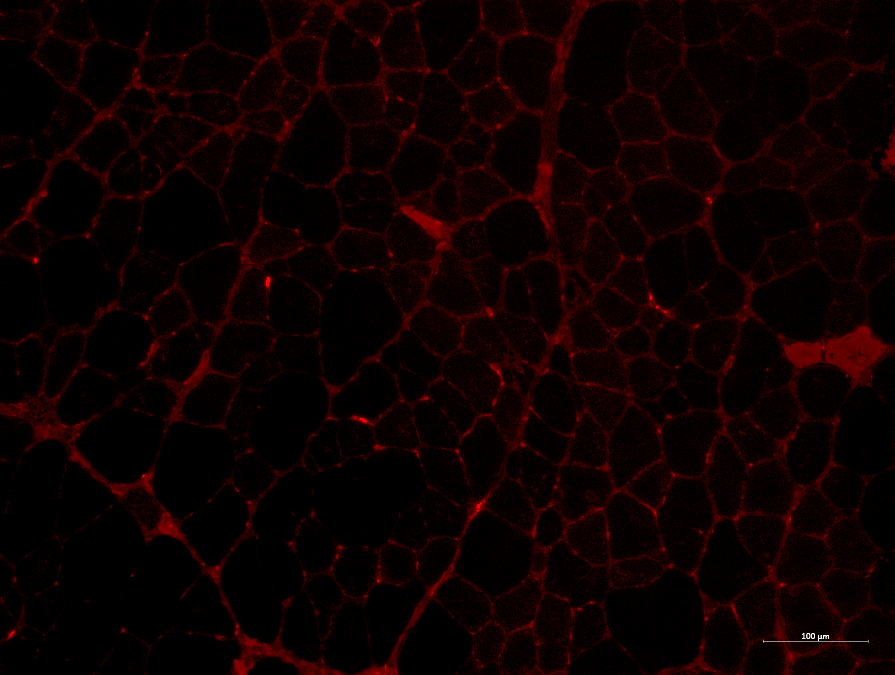

Supplement: Supplementary file 6 — Source data Fig. 4 [file 44321_2025_273_MOESM6_ESM.zip › Fig.4/Fig4-F/WT-mdx-Utrophin.tif]

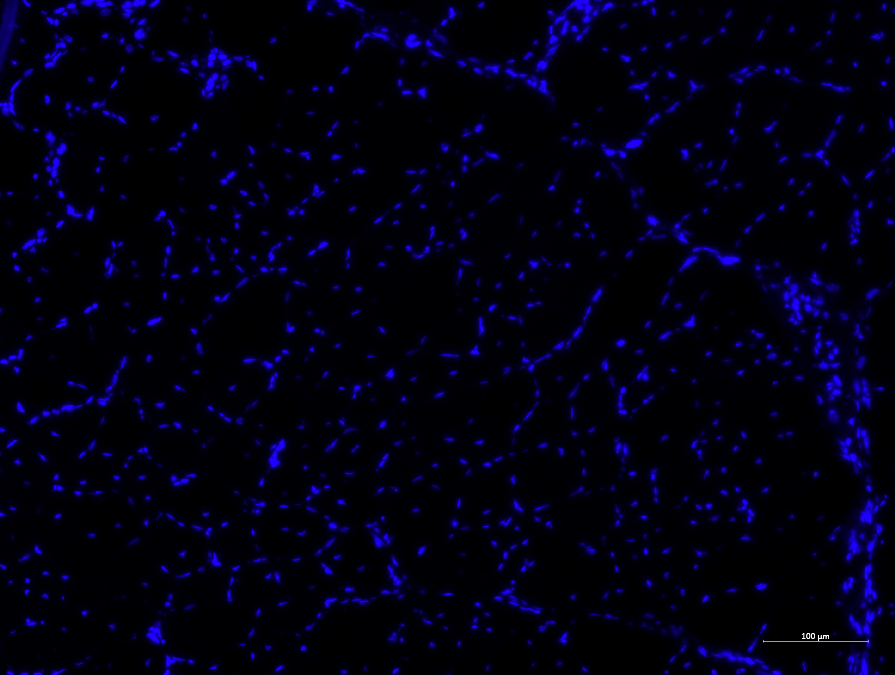

Supplement: Supplementary file 6 — Source data Fig. 4 [file 44321_2025_273_MOESM6_ESM.zip › Fig.4/Fig4-F/miR-33bKI-mdx-DAPI.tif]

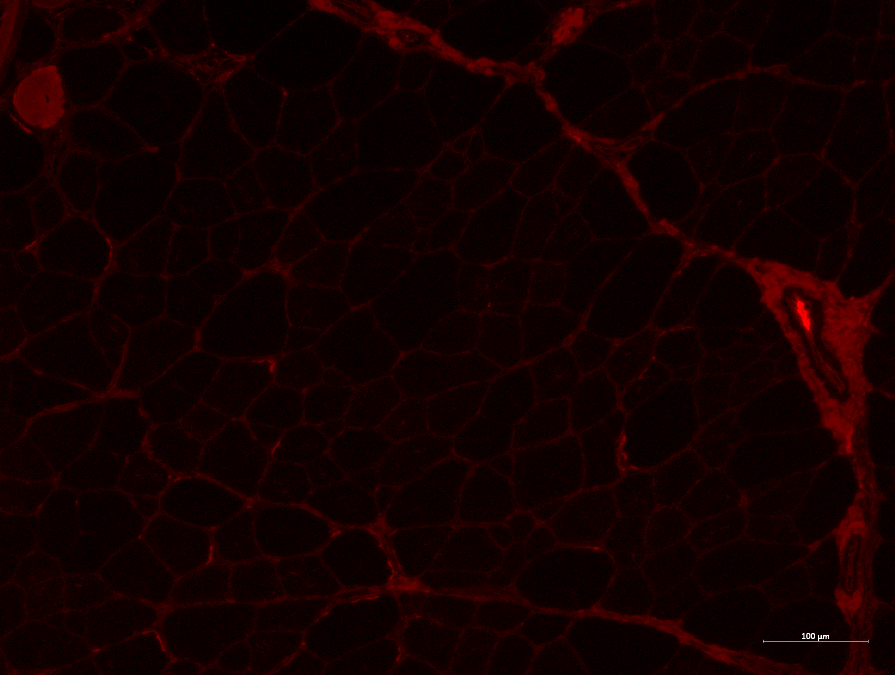

Supplement: Supplementary file 6 — Source data Fig. 4 [file 44321_2025_273_MOESM6_ESM.zip › Fig.4/Fig4-F/miR-33bKI-mdx-Utrophin.tif]

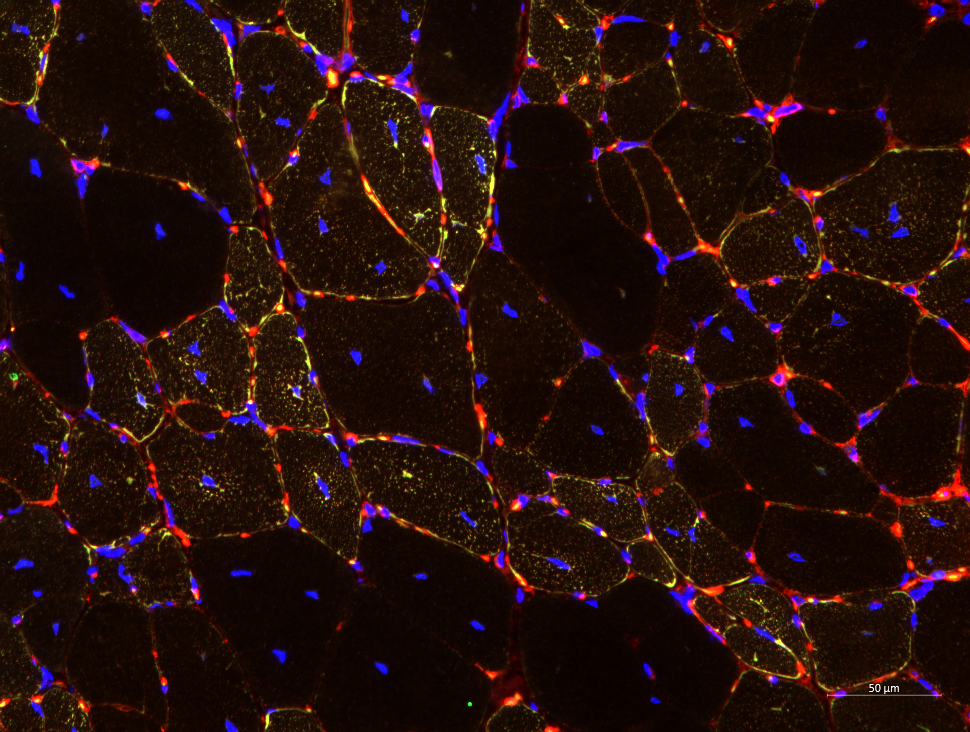

Supplement: Supplementary file 7 — Source data Fig. 5 [file 44321_2025_273_MOESM7_ESM.zip › Fig.5/Fig5-G/sh-ctl-Merge.tif]

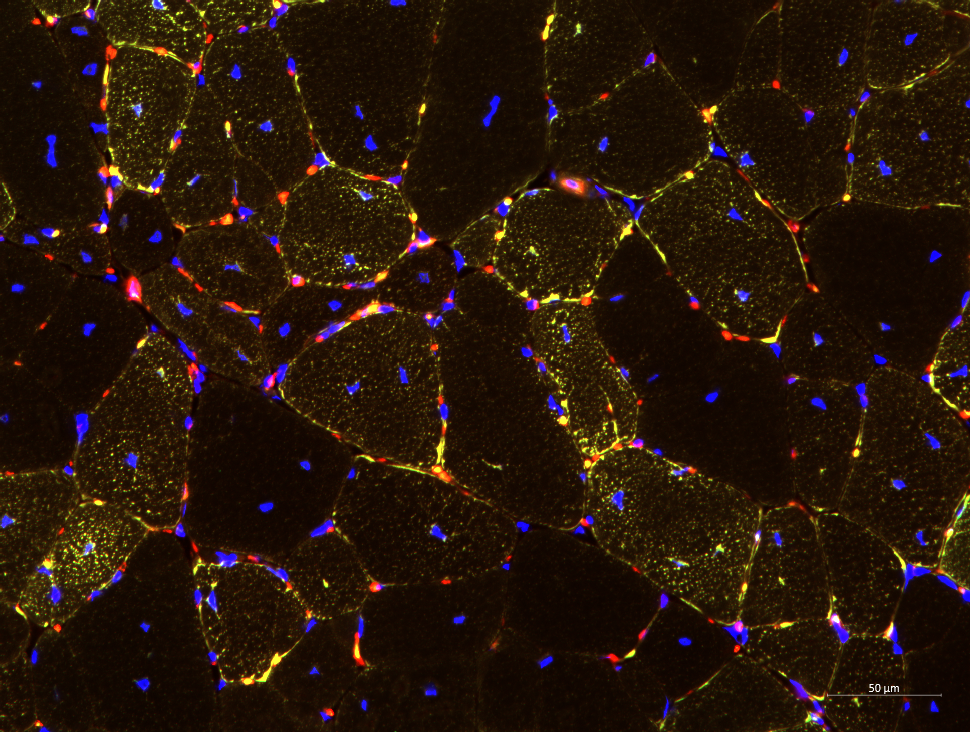

Supplement: Supplementary file 7 — Source data Fig. 5 [file 44321_2025_273_MOESM7_ESM.zip › Fig.5/Fig5-G/sh-Cdk6-Merge.tif]

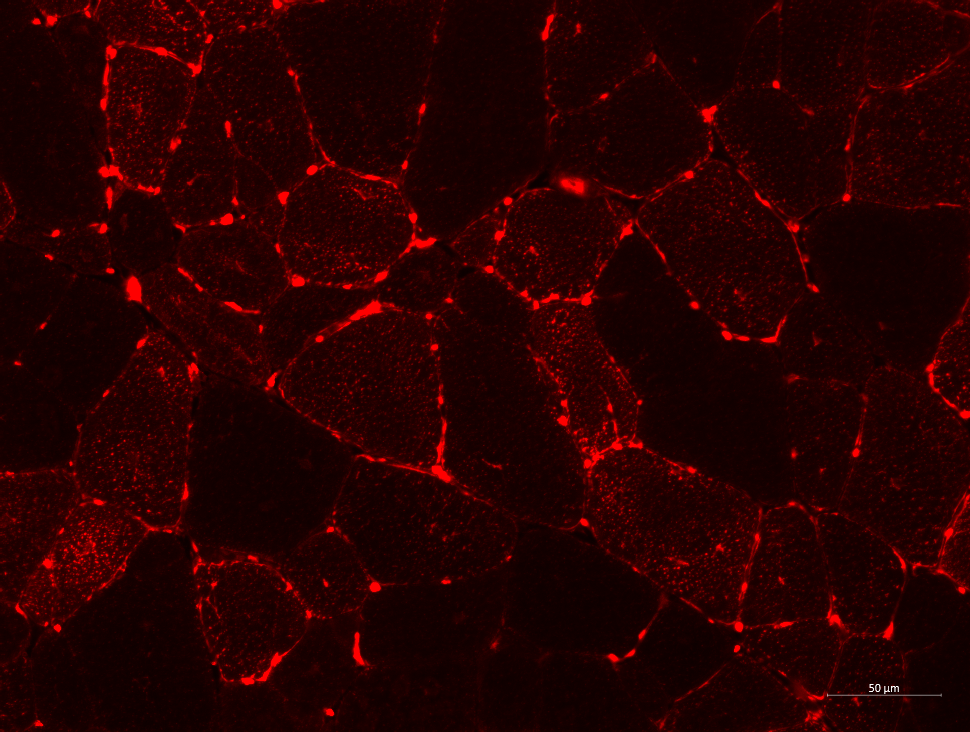

Supplement: Supplementary file 7 — Source data Fig. 5 [file 44321_2025_273_MOESM7_ESM.zip › Fig.5/Fig5-G/sh-Cdk6-pax7.tif]

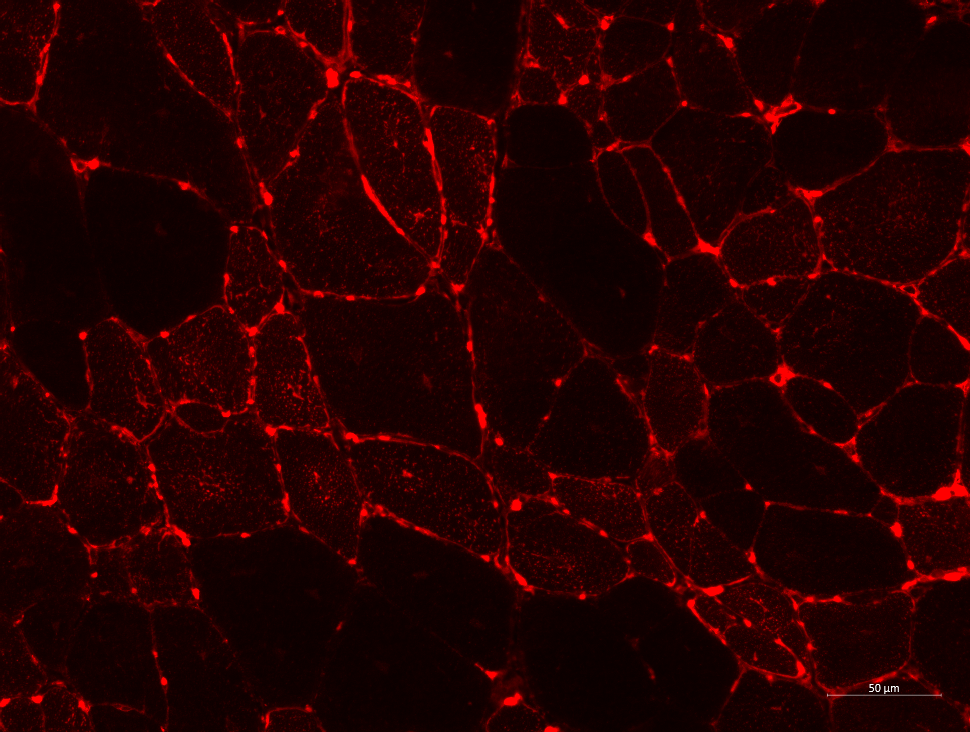

Supplement: Supplementary file 7 — Source data Fig. 5 [file 44321_2025_273_MOESM7_ESM.zip › Fig.5/Fig5-G/sh-ctl-pax7.tif]

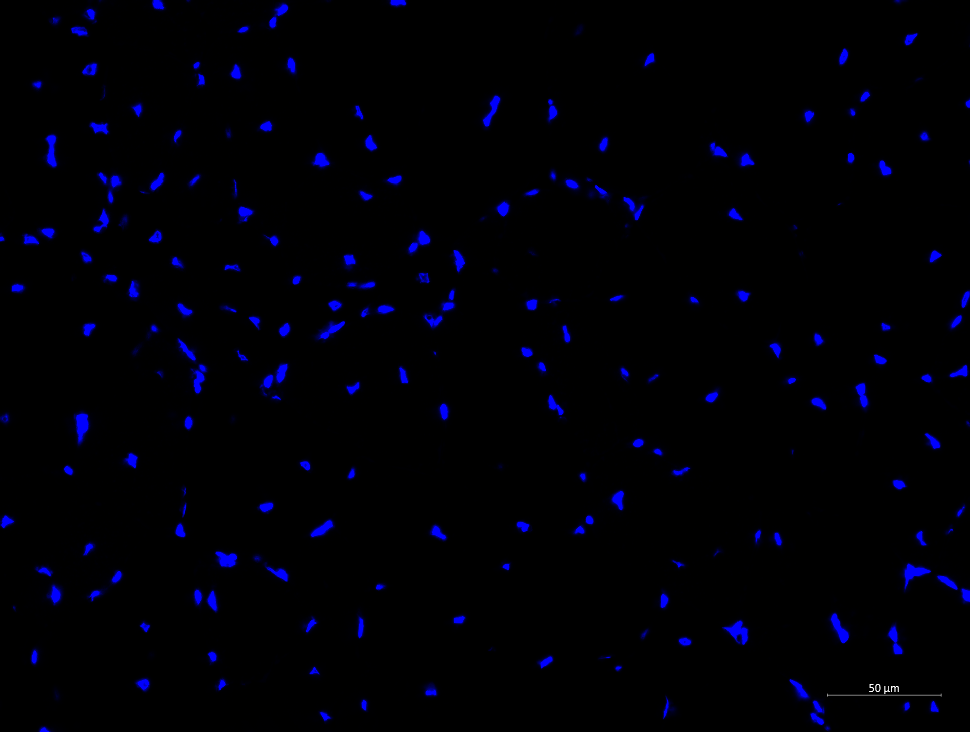

Supplement: Supplementary file 7 — Source data Fig. 5 [file 44321_2025_273_MOESM7_ESM.zip › Fig.5/Fig5-G/sh-Cdk6-DAPI.tif]

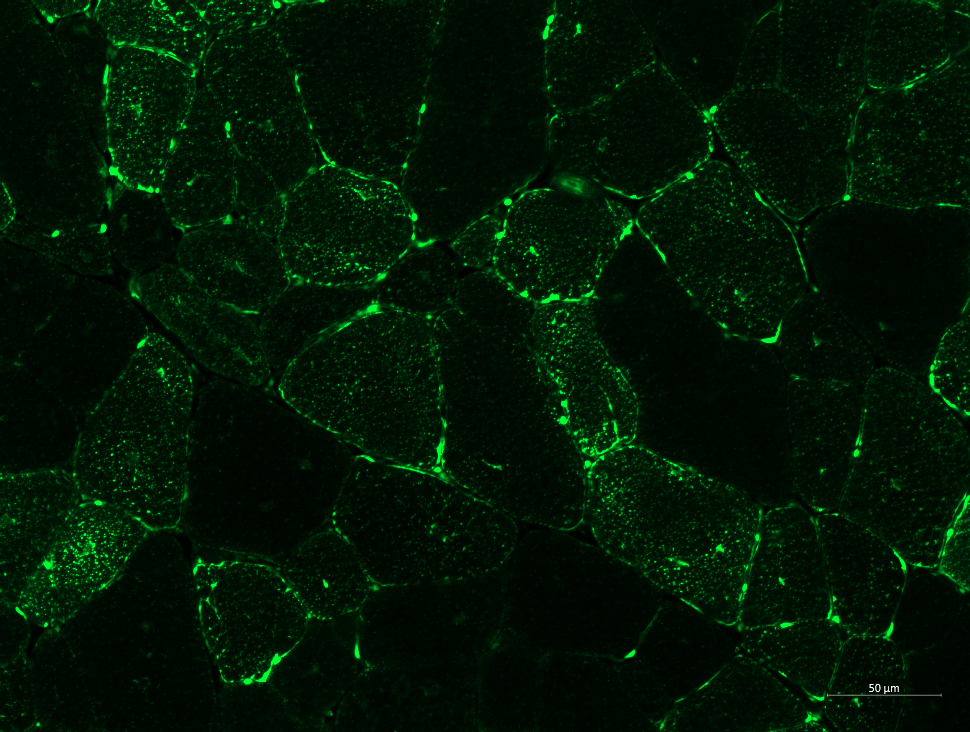

Supplement: Supplementary file 7 — Source data Fig. 5 [file 44321_2025_273_MOESM7_ESM.zip › Fig.5/Fig5-G/sh-Cdk6-MyoD.tif]

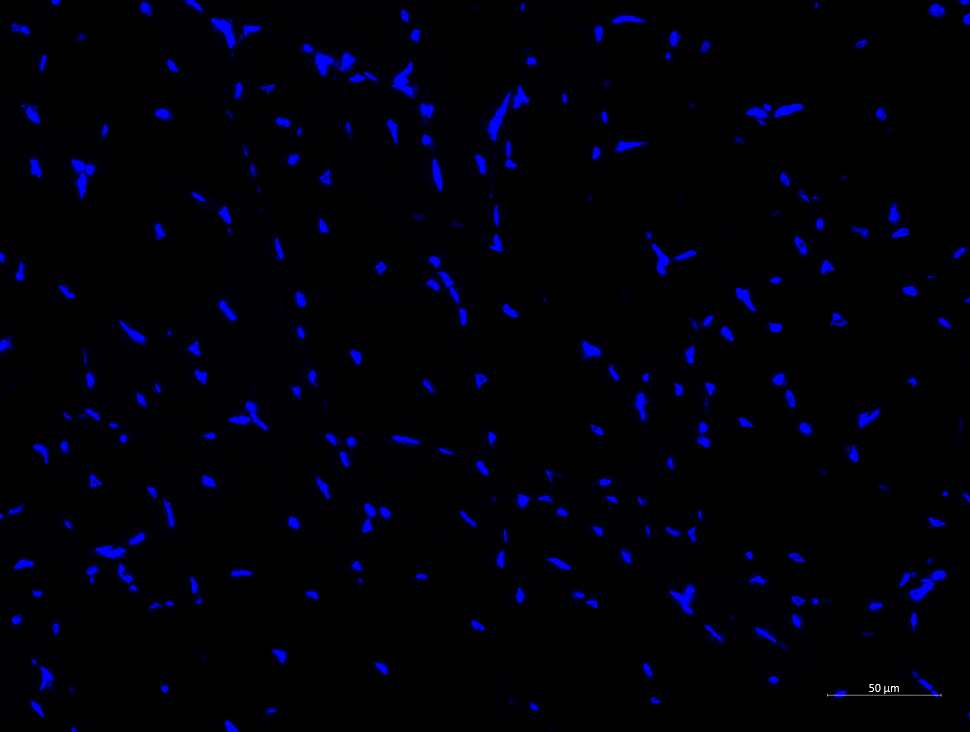

Supplement: Supplementary file 7 — Source data Fig. 5 [file 44321_2025_273_MOESM7_ESM.zip › Fig.5/Fig5-G/sh-ctl-DAPI.tif]

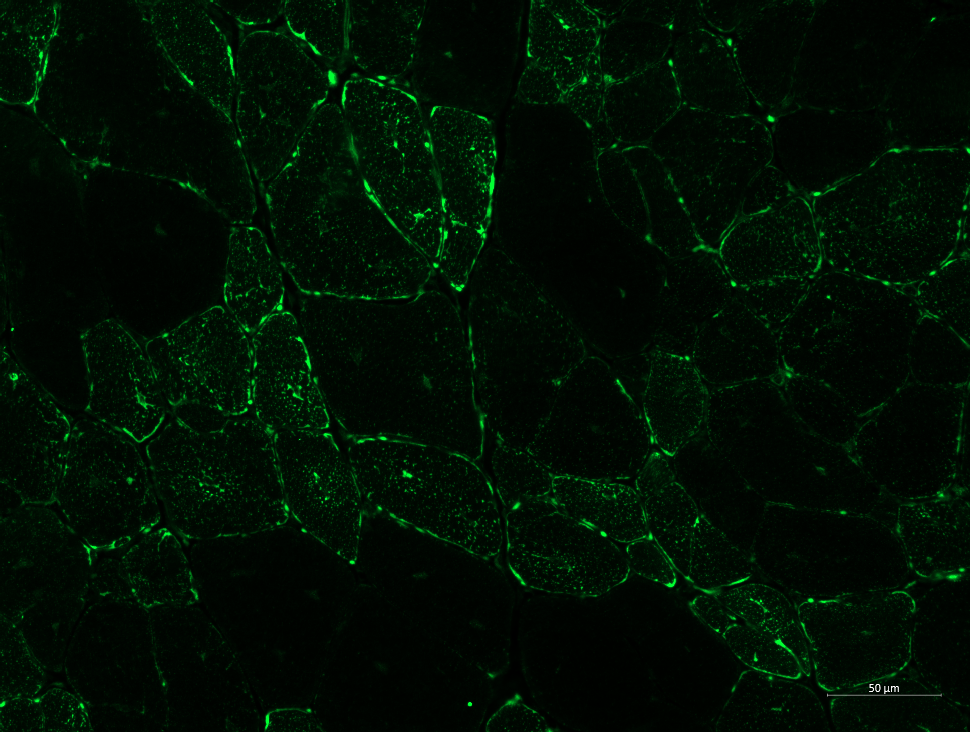

Supplement: Supplementary file 7 — Source data Fig. 5 [file 44321_2025_273_MOESM7_ESM.zip › Fig.5/Fig5-G/sh-ctl-MyoD.tif]

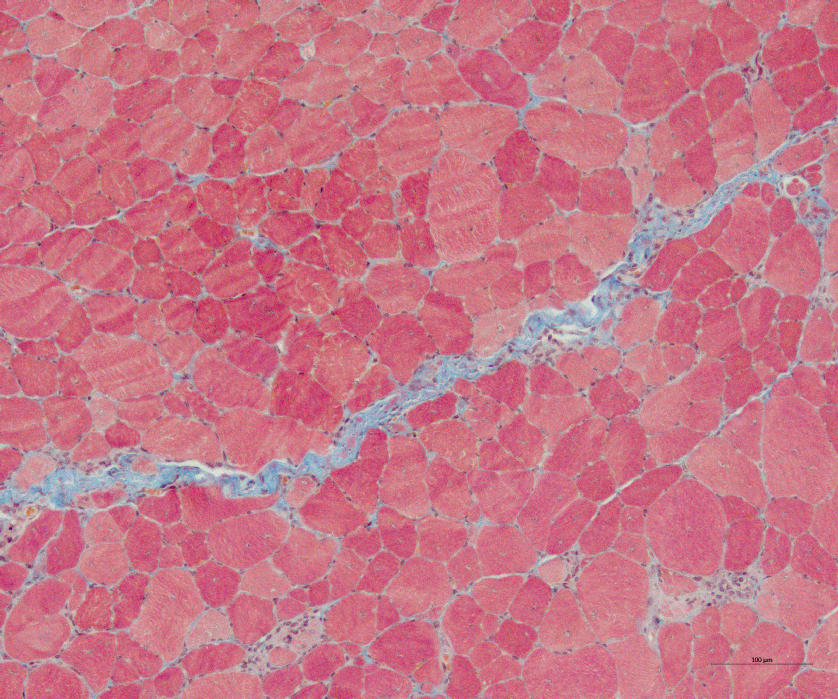

Supplement: Supplementary file 7 — Source data Fig. 5 [file 44321_2025_273_MOESM7_ESM.zip › Fig.5/Fig5-E/shCdk6.tif]

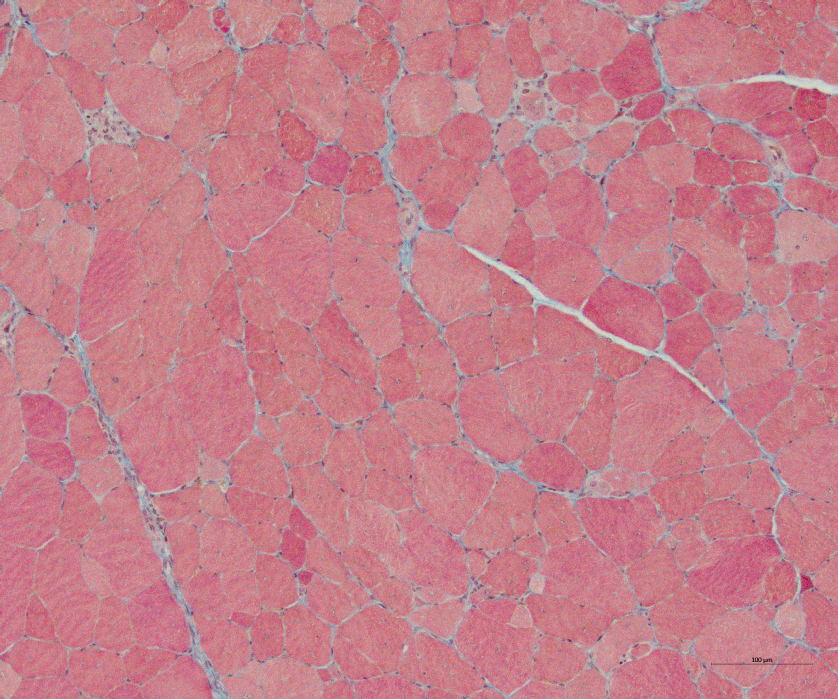

Supplement: Supplementary file 7 — Source data Fig. 5 [file 44321_2025_273_MOESM7_ESM.zip › Fig.5/Fig5-E/sh-Ctl.tif]

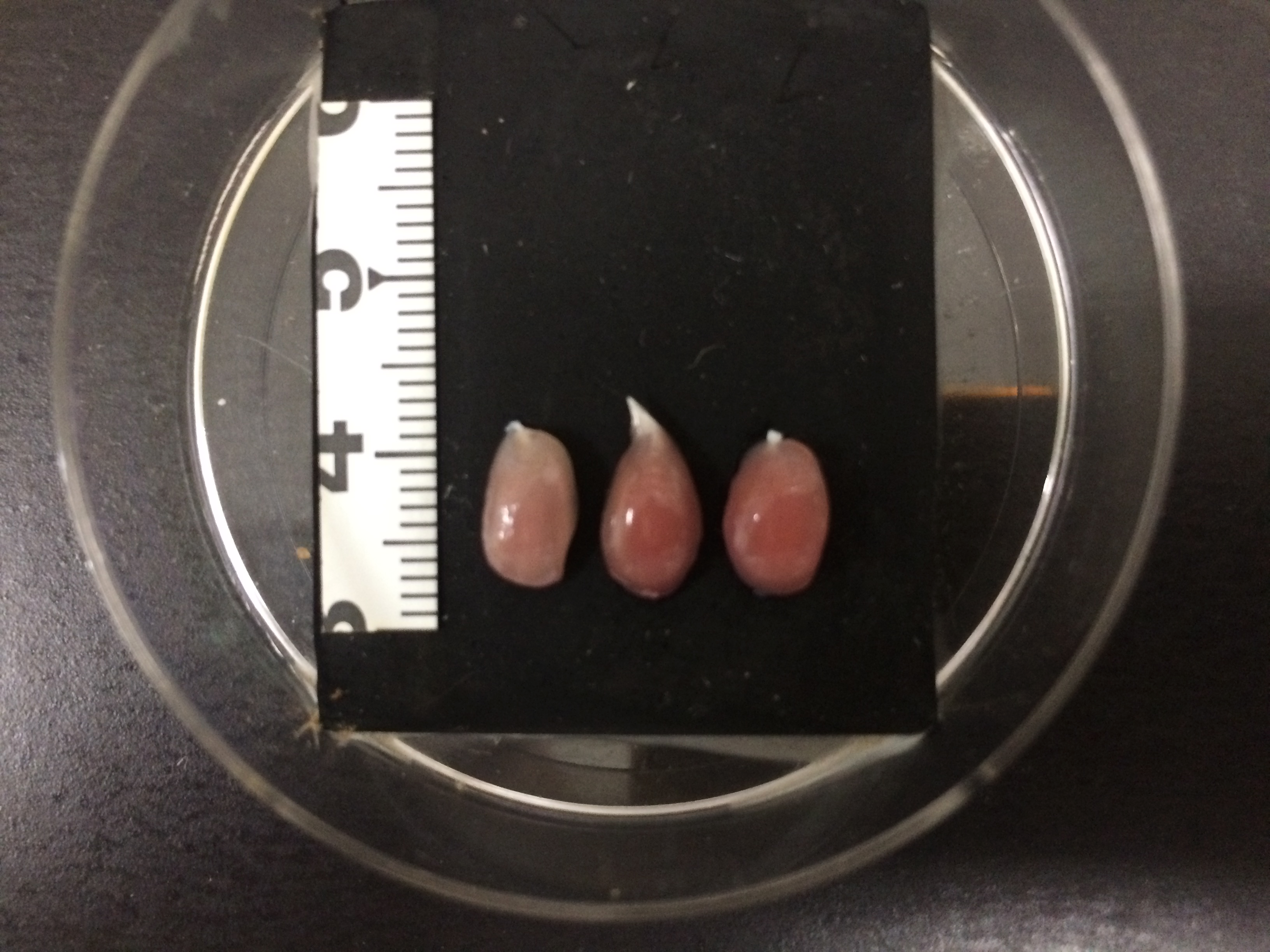

Supplement: Supplementary file 7 — Source data Fig. 5 [file 44321_2025_273_MOESM7_ESM.zip › Fig.5/Fig5-B/IMG_0602[1].tif]

shCtl      shCdk6

Cdk6

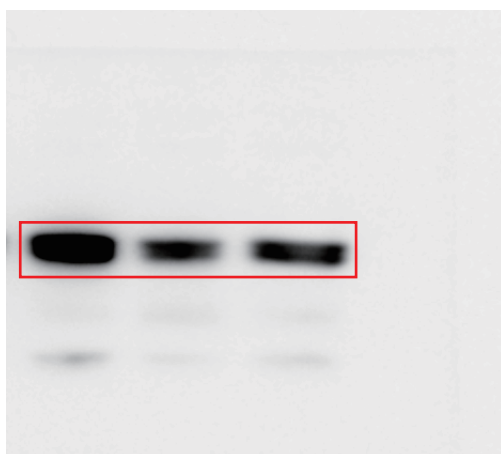

— 31

GAPDH

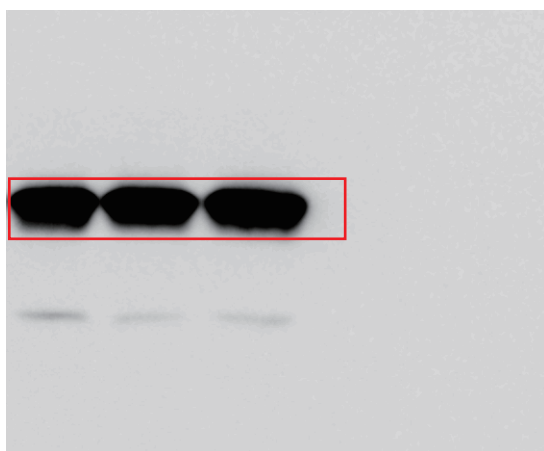

— 36

Supplement: Supplementary file 7 — Source data Fig. 5 [file 44321_2025_273_MOESM7_ESM.zip › Fig.5/Fig5-D/Fig5-D.pdf]

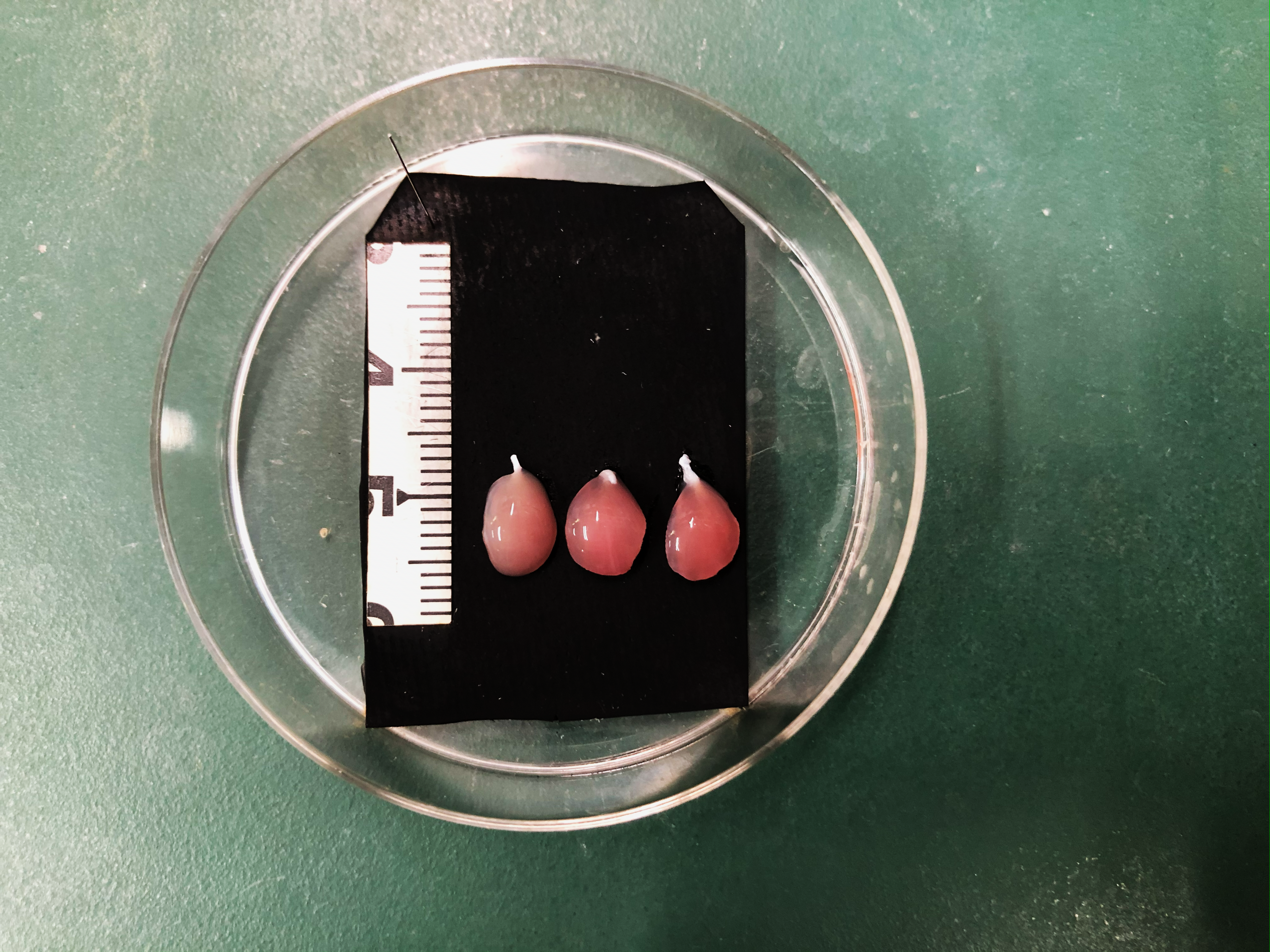

Supplement: Supplementary file 8 — Source data Fig. 6 [file 44321_2025_273_MOESM8_ESM.zip › Fig.6/Fig6-A/IMG_1160[1].tif]

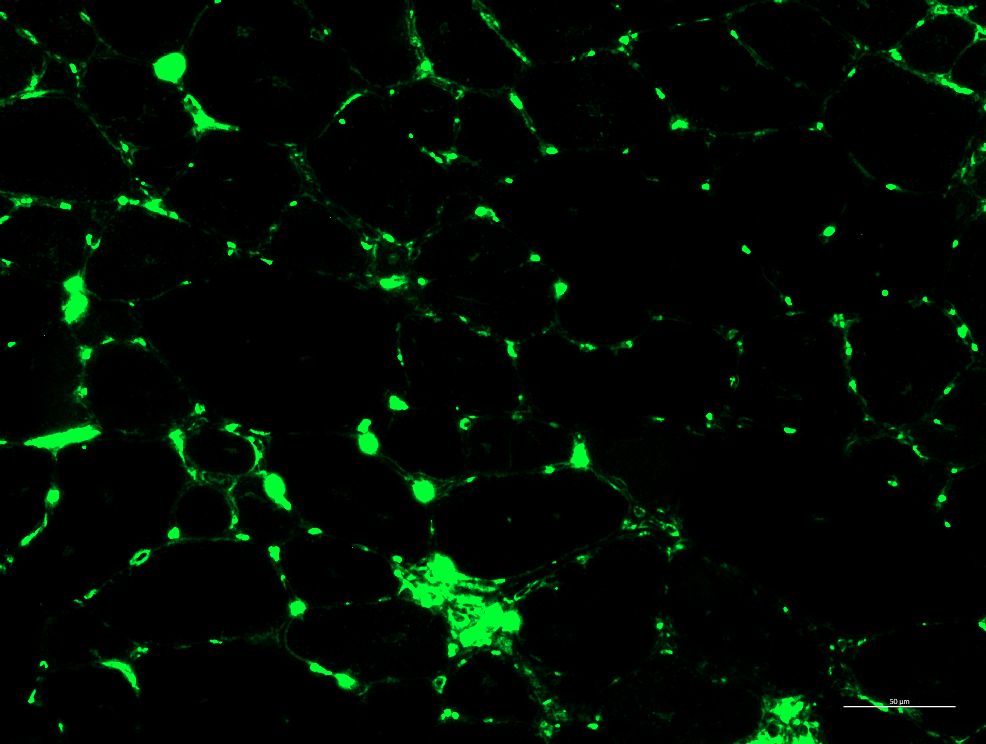

Supplement: Supplementary file 8 — Source data Fig. 6 [file 44321_2025_273_MOESM8_ESM.zip › Fig.6/Fig6-F/shFst-Myod.tif]

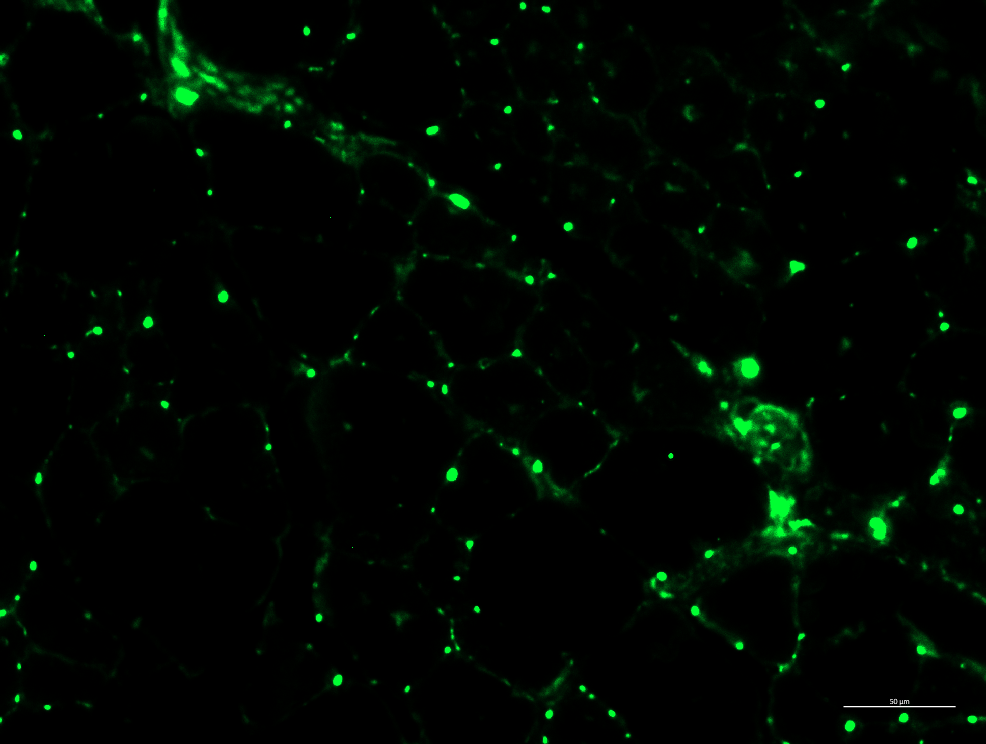

Supplement: Supplementary file 8 — Source data Fig. 6 [file 44321_2025_273_MOESM8_ESM.zip › Fig.6/Fig6-F/shCtl-Myod.tif]

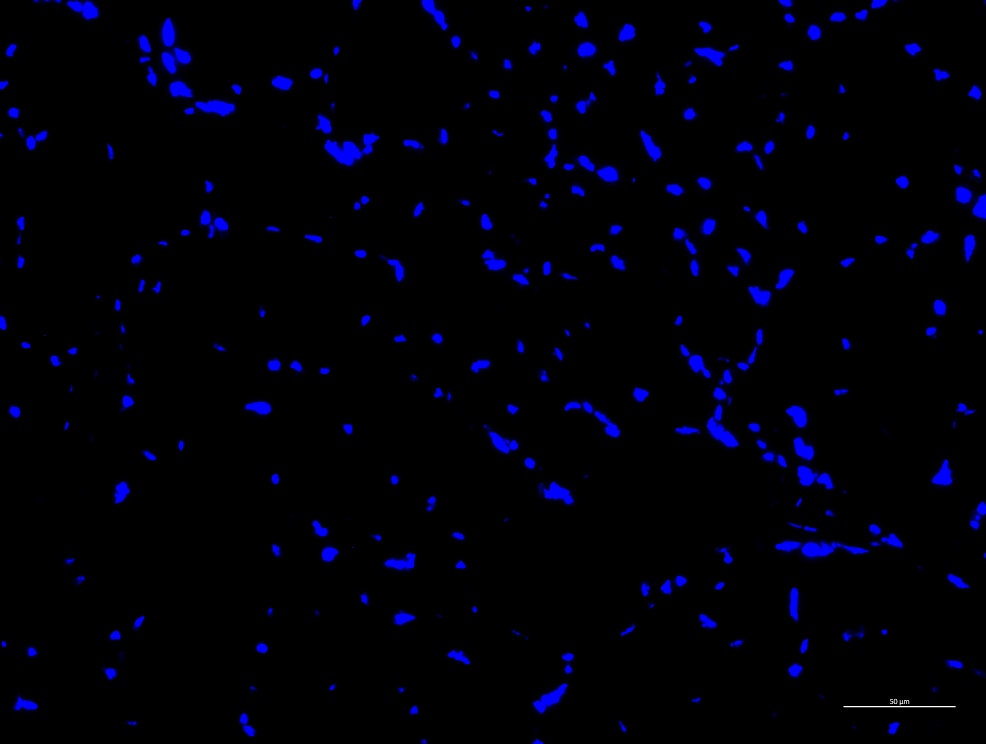

Supplement: Supplementary file 8 — Source data Fig. 6 [file 44321_2025_273_MOESM8_ESM.zip › Fig.6/Fig6-F/shCtl-DAPI.tif]

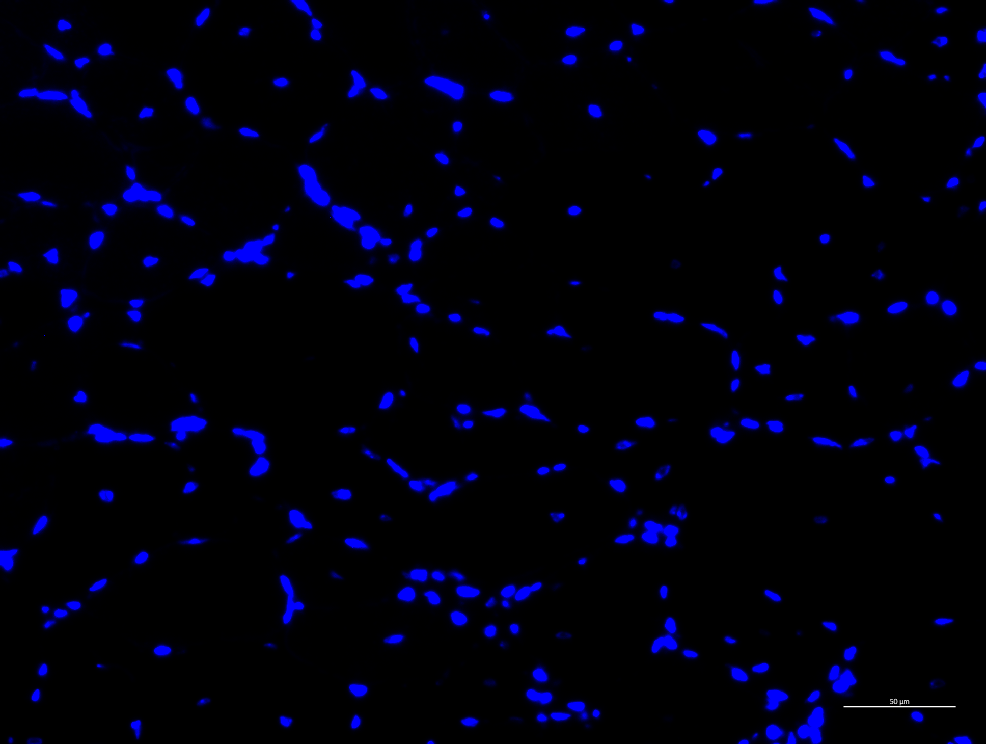

Supplement: Supplementary file 8 — Source data Fig. 6 [file 44321_2025_273_MOESM8_ESM.zip › Fig.6/Fig6-F/shFst-DAPI.tif]

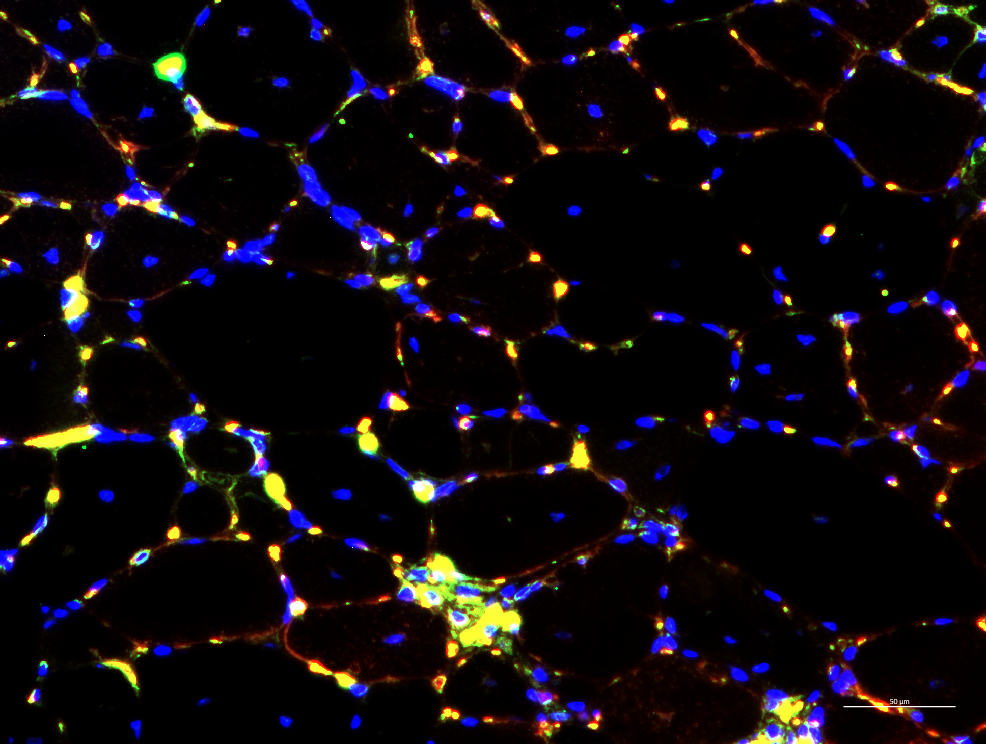

Supplement: Supplementary file 8 — Source data Fig. 6 [file 44321_2025_273_MOESM8_ESM.zip › Fig.6/Fig6-F/shFst-Merge.tif]

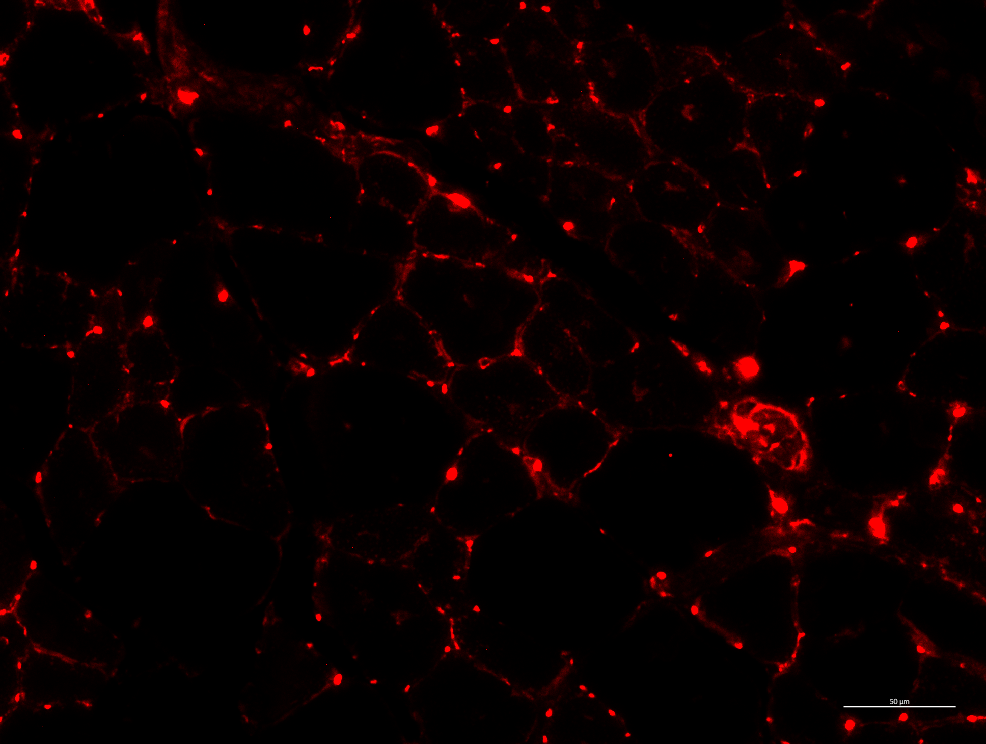

Supplement: Supplementary file 8 — Source data Fig. 6 [file 44321_2025_273_MOESM8_ESM.zip › Fig.6/Fig6-F/shCtl-Pax7.tif]

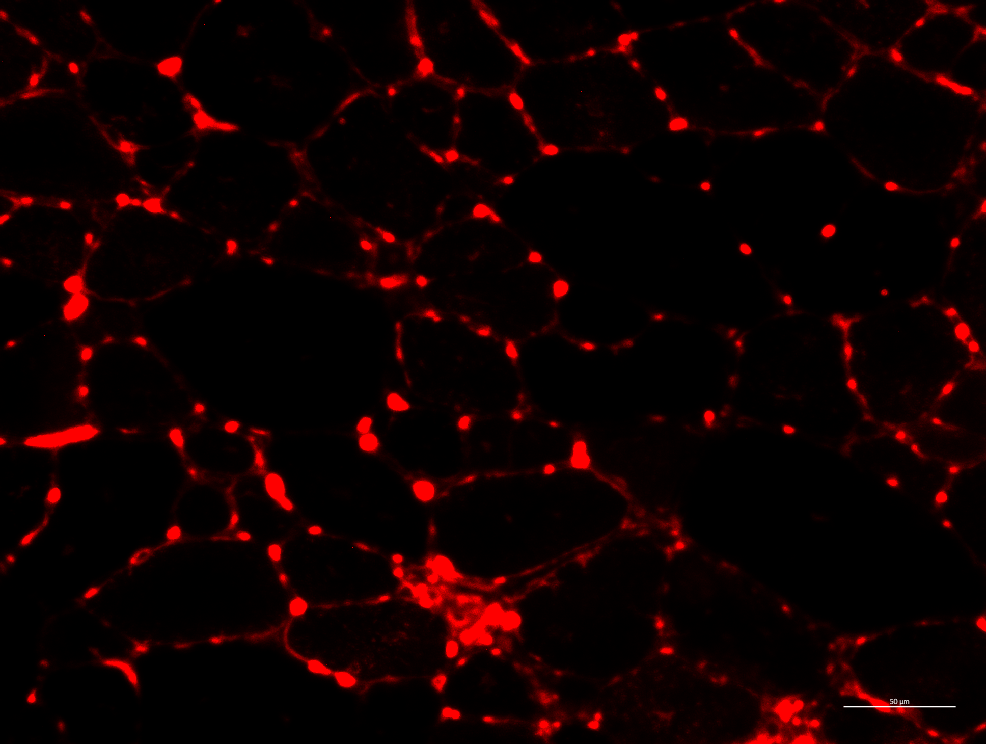

Supplement: Supplementary file 8 — Source data Fig. 6 [file 44321_2025_273_MOESM8_ESM.zip › Fig.6/Fig6-F/shFst-Pax7.tif]

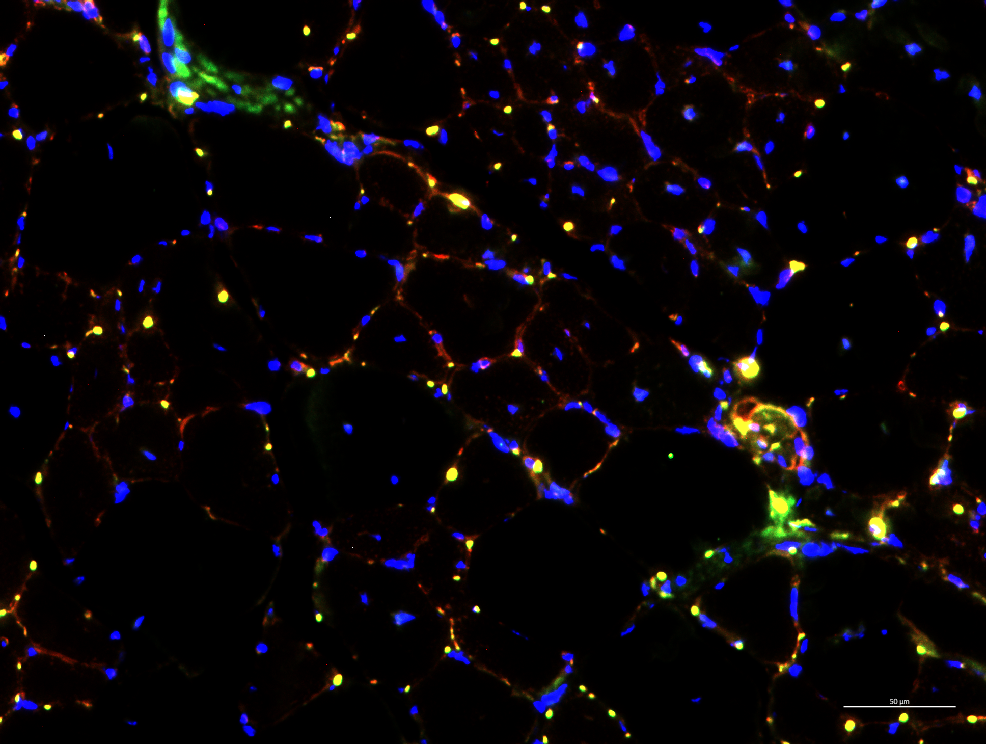

Supplement: Supplementary file 8 — Source data Fig. 6 [file 44321_2025_273_MOESM8_ESM.zip › Fig.6/Fig6-F/shCtl-Merge.tif]

**Fig6-C**

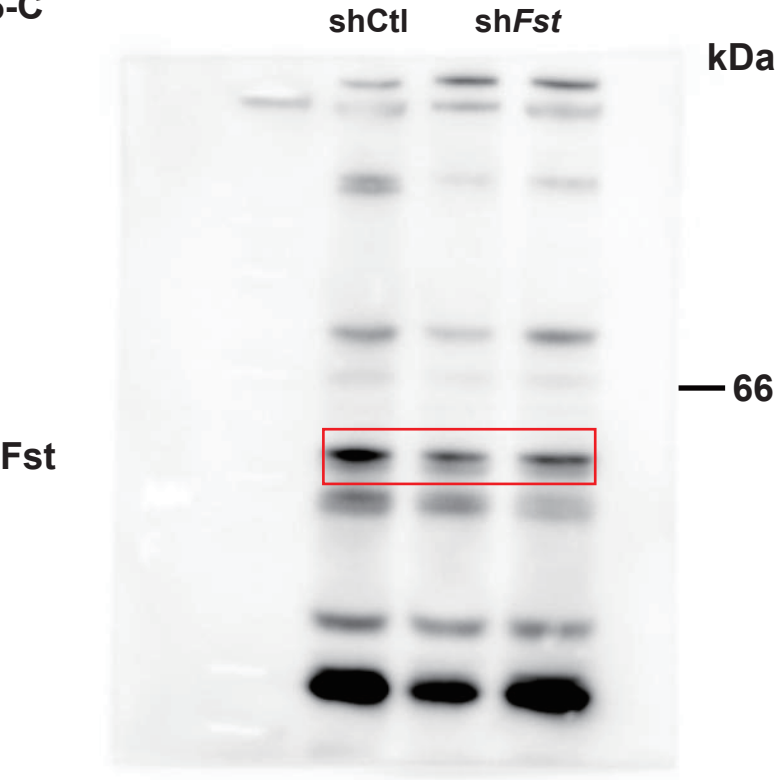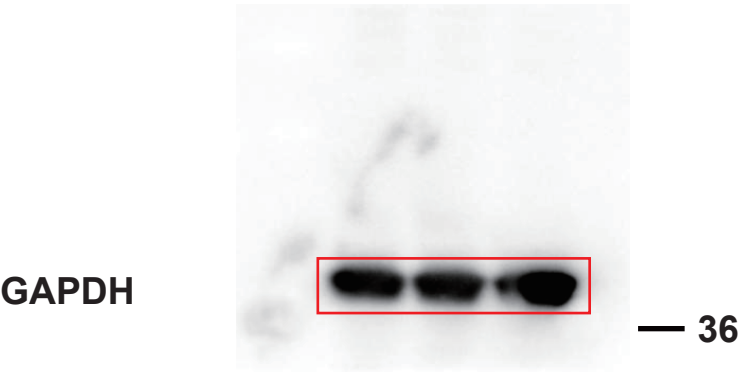

Supplement: Supplementary file 8 — Source data Fig. 6 [file 44321_2025_273_MOESM8_ESM.zip › Fig.6/Fig6-C/Fig.6-C.pdf]

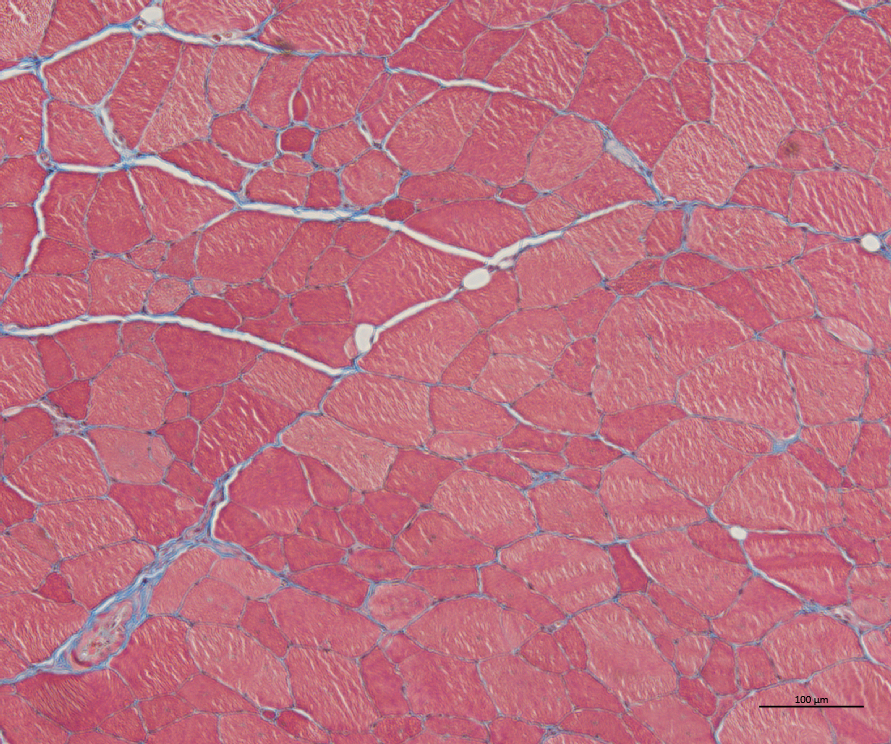

Supplement: Supplementary file 8 — Source data Fig. 6 [file 44321_2025_273_MOESM8_ESM.zip › Fig.6/Fig6-D/shctl.tif]

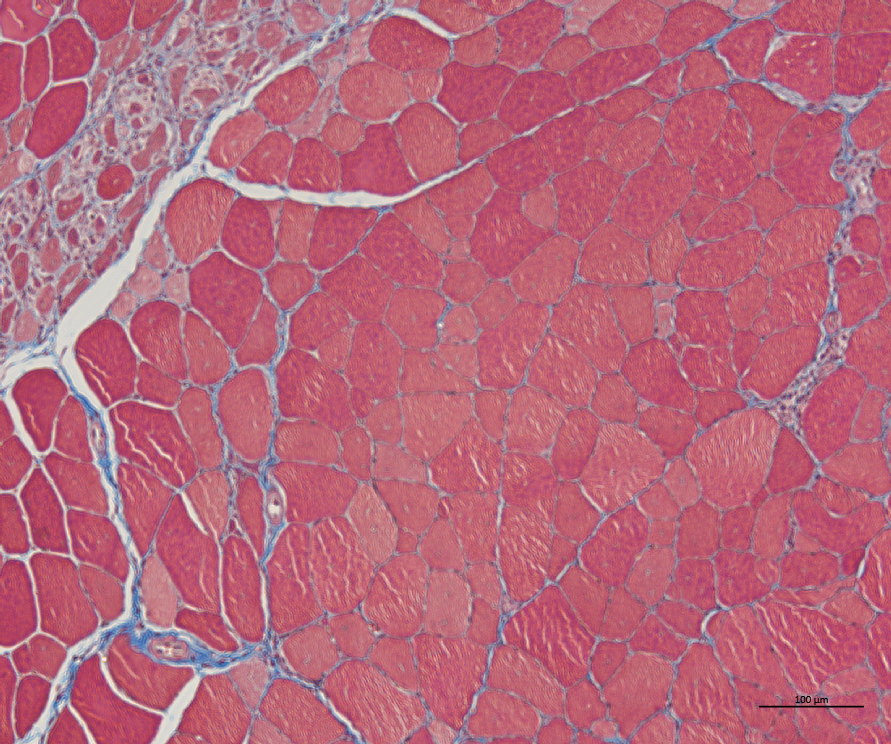

Supplement: Supplementary file 8 — Source data Fig. 6 [file 44321_2025_273_MOESM8_ESM.zip › Fig.6/Fig6-D/shFst.tif]

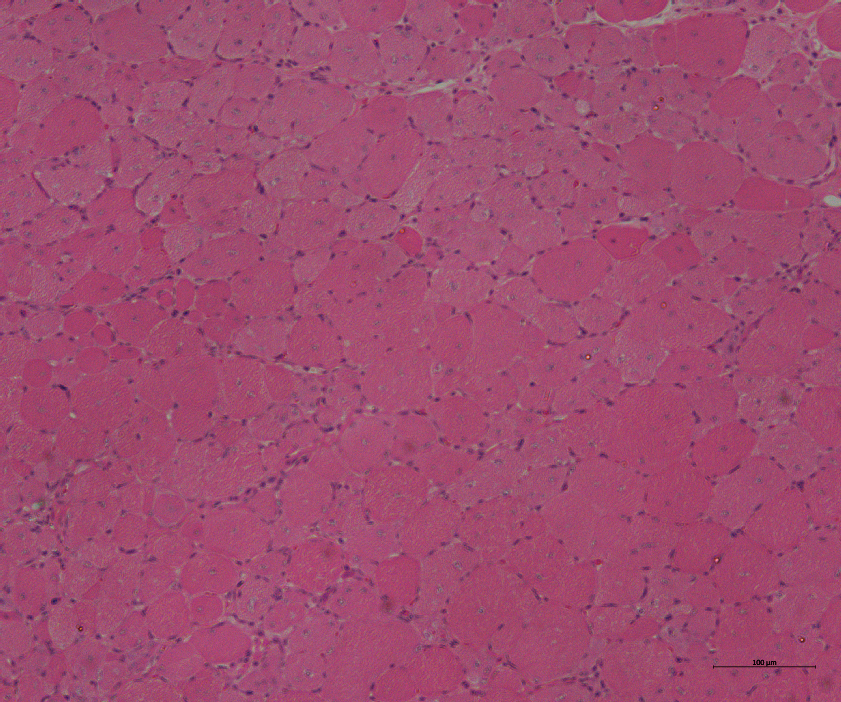

Supplement: Supplementary file 9 — Source data Fig. 7 [file 44321_2025_273_MOESM9_ESM.zip › Fig.7/Fig7-B/Anti-miR-33b.tif]

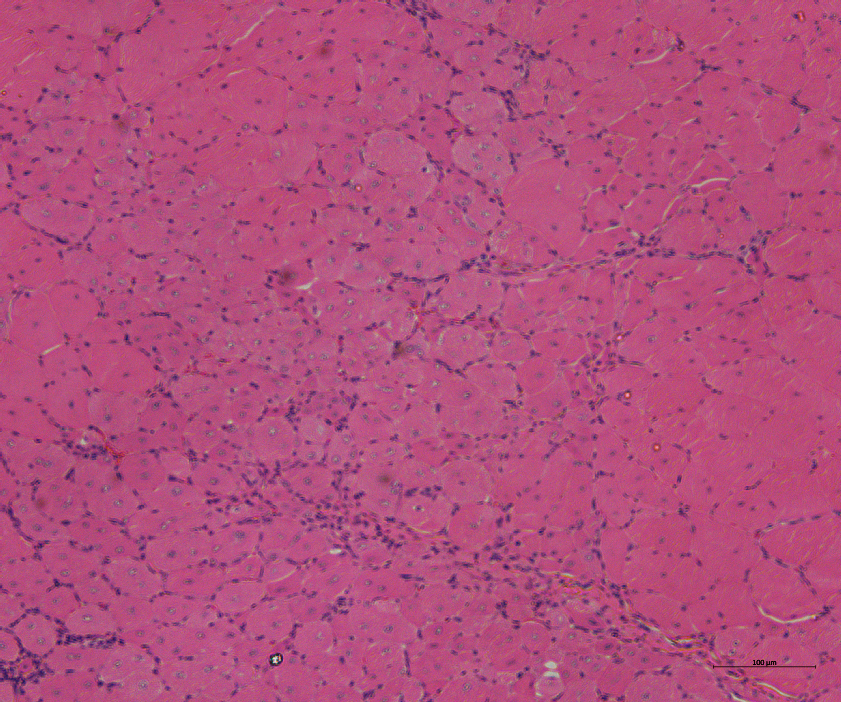

Supplement: Supplementary file 9 — Source data Fig. 7 [file 44321_2025_273_MOESM9_ESM.zip › Fig.7/Fig7-B/Control AmNA.tif]

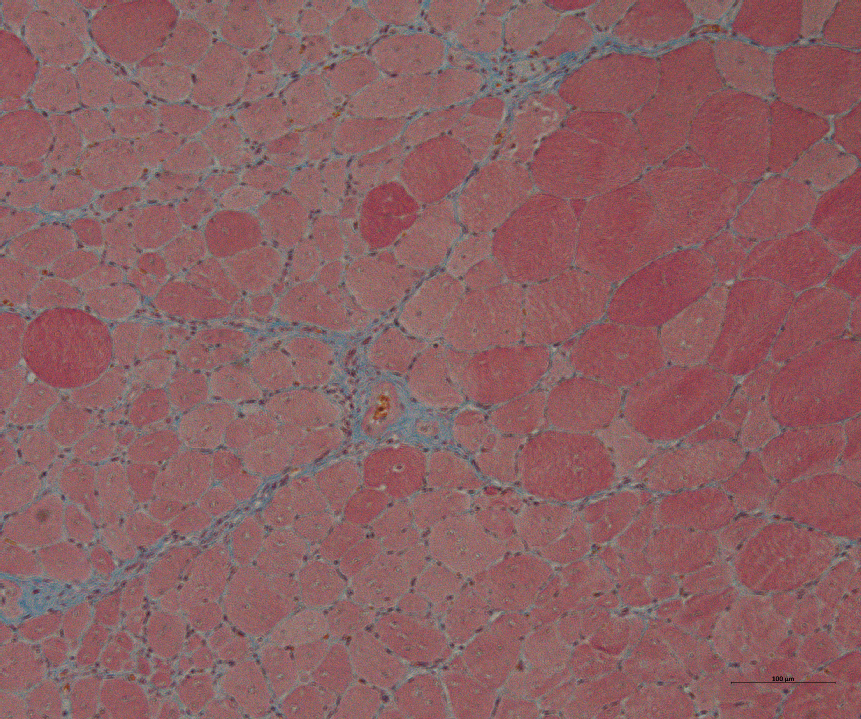

Supplement: Supplementary file 9 — Source data Fig. 7 [file 44321_2025_273_MOESM9_ESM.zip › Fig.7/Fig7-D/Anti-miR-33b.tif]

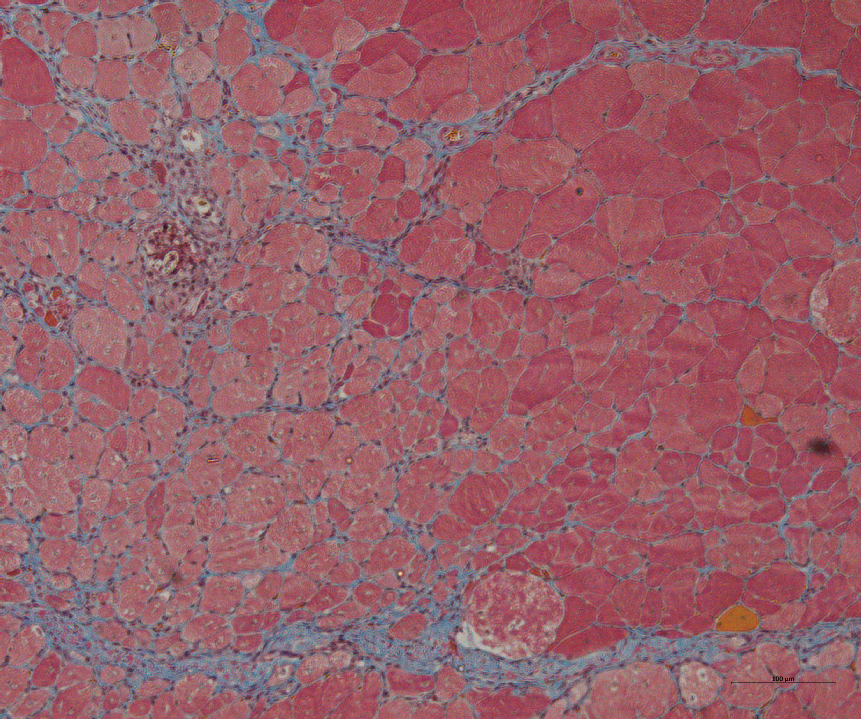

Supplement: Supplementary file 9 — Source data Fig. 7 [file 44321_2025_273_MOESM9_ESM.zip › Fig.7/Fig7-D/Control AmNA.tif]

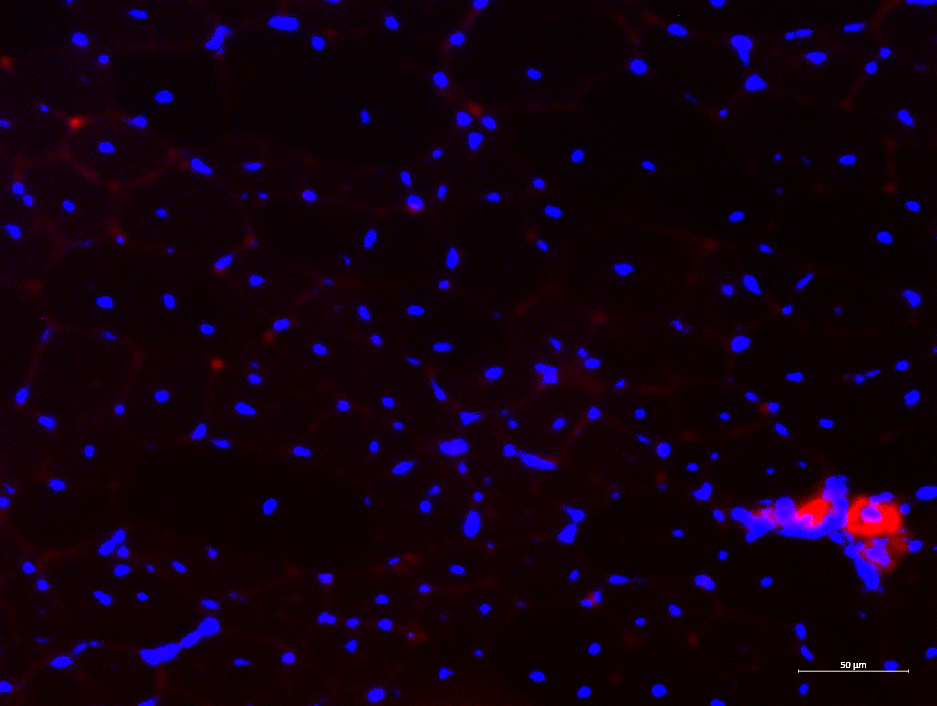

Supplement: Supplementary file 9 — Source data Fig. 7 [file 44321_2025_273_MOESM9_ESM.zip › Fig.7/Fig7-F/Control-AmNA-Merge.tif]

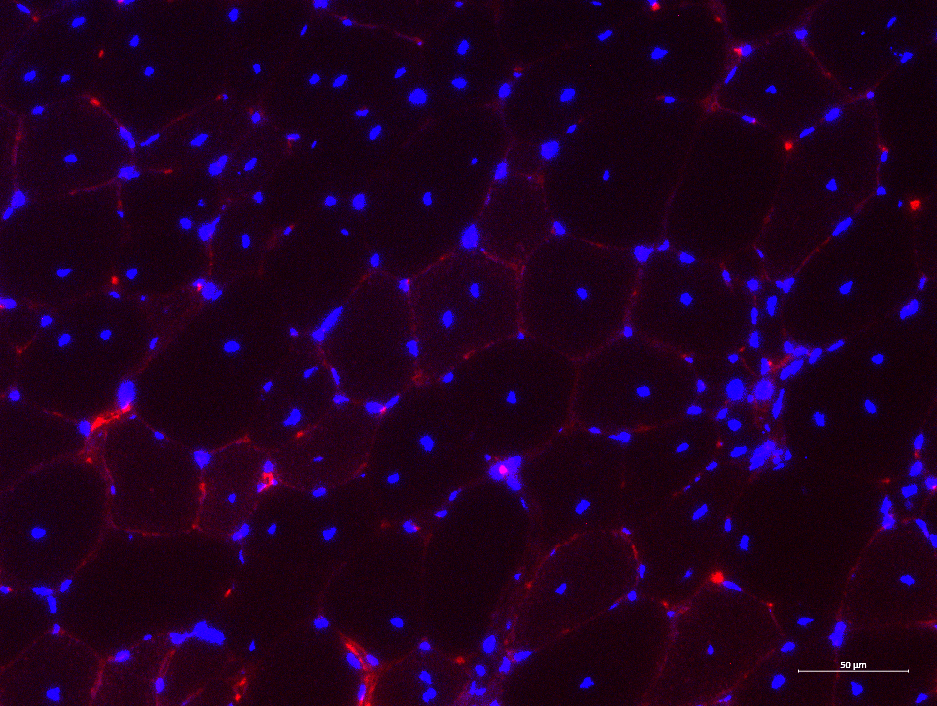

Supplement: Supplementary file 9 — Source data Fig. 7 [file 44321_2025_273_MOESM9_ESM.zip › Fig.7/Fig7-F/Anti-miR-33b-Merge.tif]

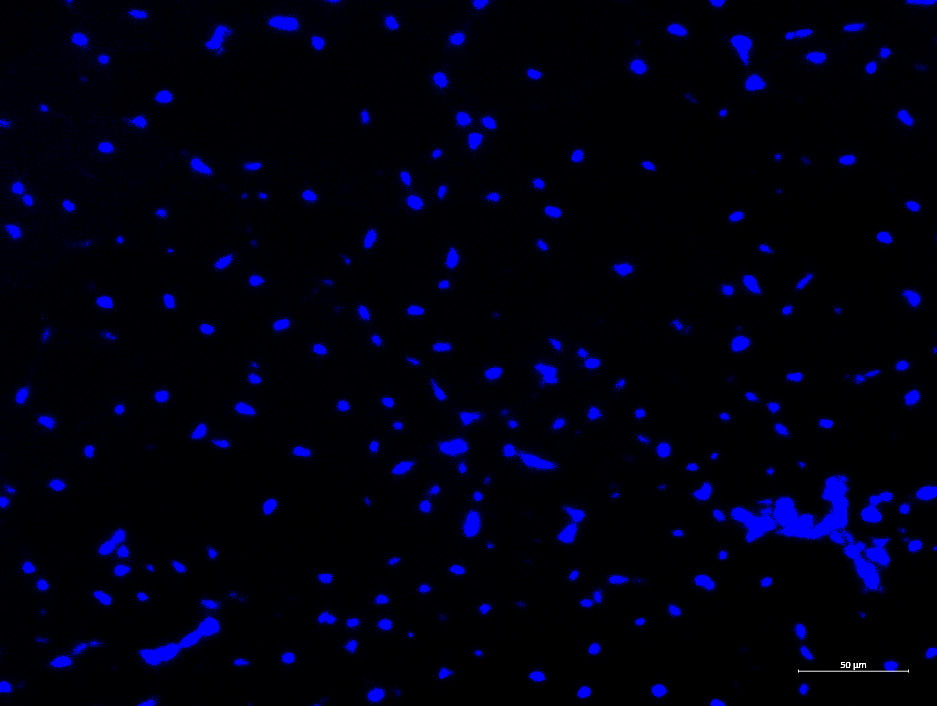

Supplement: Supplementary file 9 — Source data Fig. 7 [file 44321_2025_273_MOESM9_ESM.zip › Fig.7/Fig7-F/Control-AmNA-DAPI.tif]

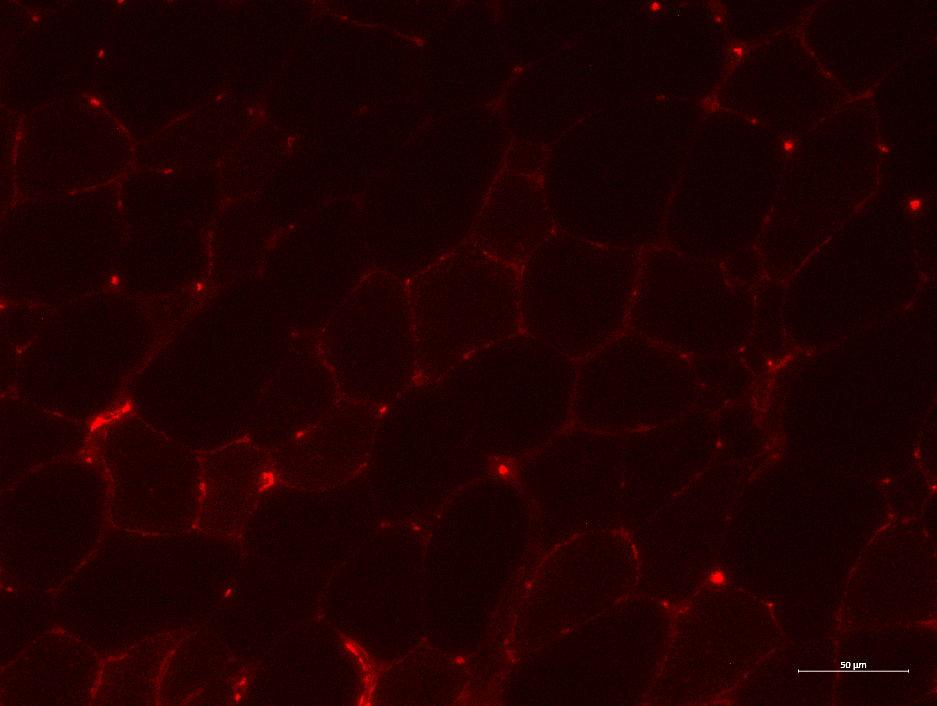

Supplement: Supplementary file 9 — Source data Fig. 7 [file 44321_2025_273_MOESM9_ESM.zip › Fig.7/Fig7-F/Anti-miR-33b-Utrophin.tif]

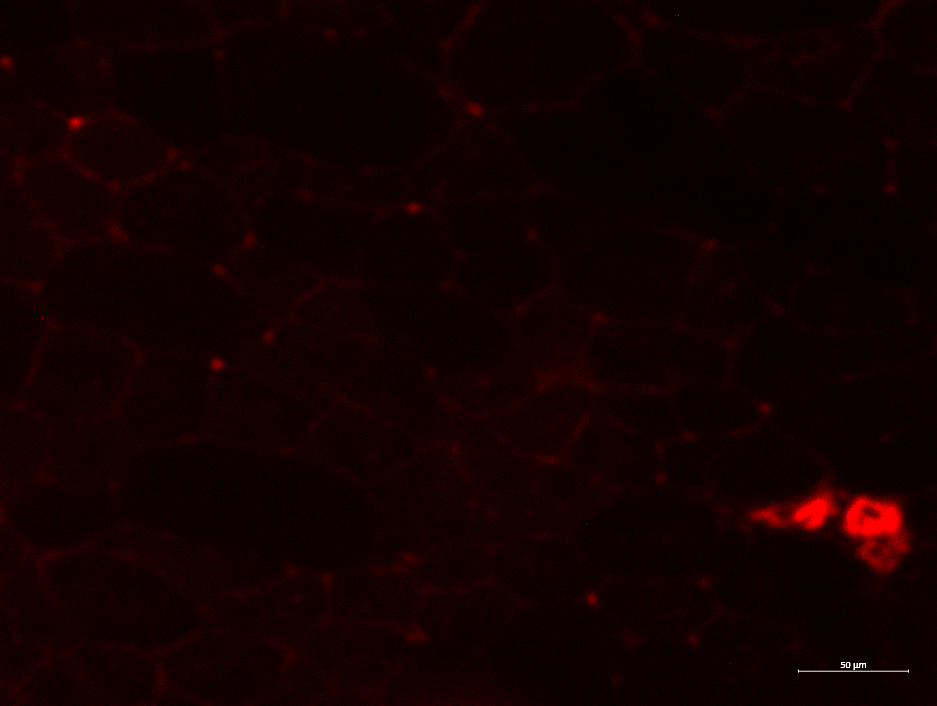

Supplement: Supplementary file 9 — Source data Fig. 7 [file 44321_2025_273_MOESM9_ESM.zip › Fig.7/Fig7-F/Control-AmNA-Utrophin.tif]

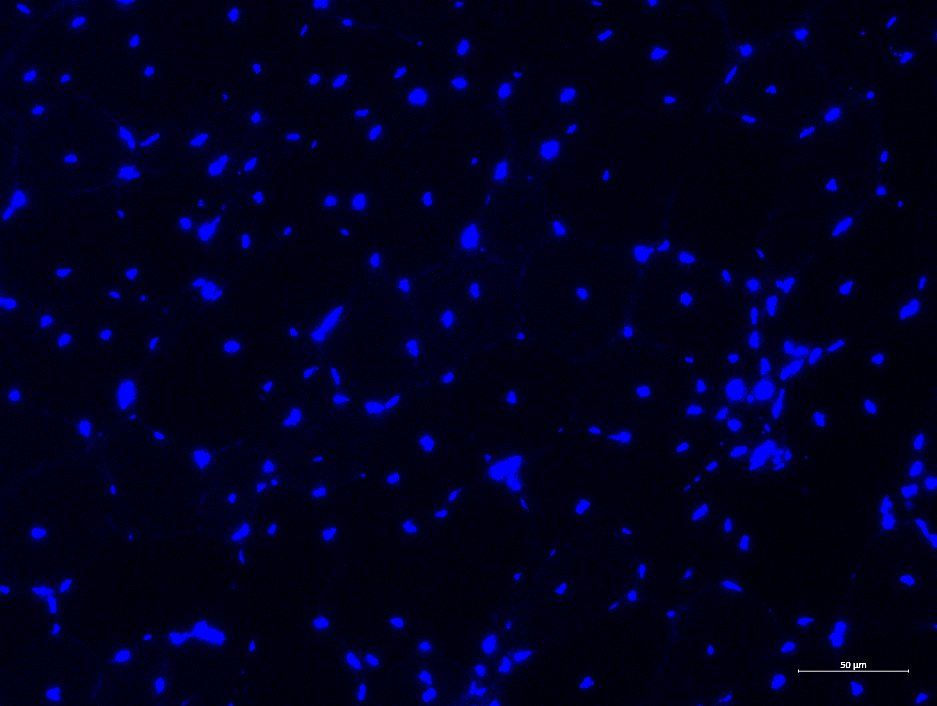

Supplement: Supplementary file 9 — Source data Fig. 7 [file 44321_2025_273_MOESM9_ESM.zip › Fig.7/Fig7-F/Anti-miR-33b-DAPI.tif]

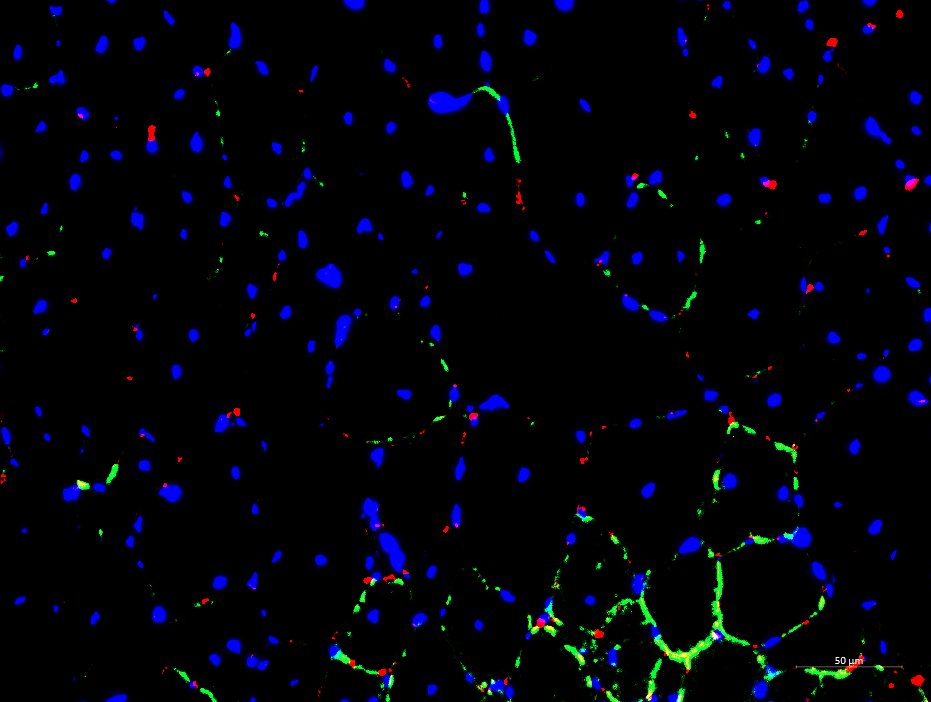

Supplement: Supplementary file 9 — Source data Fig. 7 [file 44321_2025_273_MOESM9_ESM.zip › Fig.7/Fig7-G/Control-AmNA-Merge.tif]

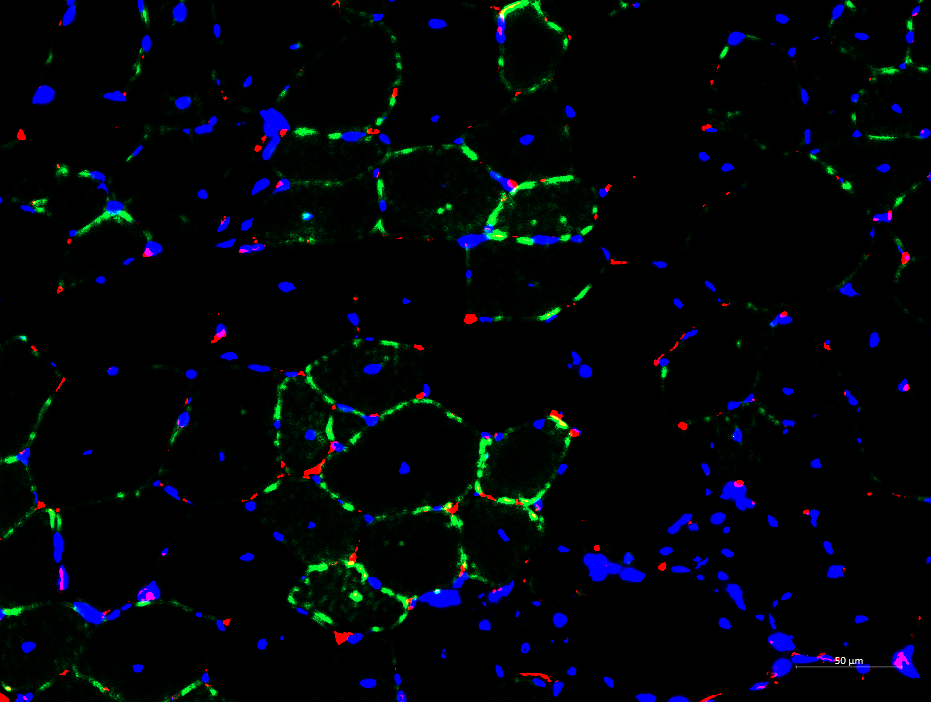

Supplement: Supplementary file 9 — Source data Fig. 7 [file 44321_2025_273_MOESM9_ESM.zip › Fig.7/Fig7-G/Anti-miR-33b-Merge.tif]

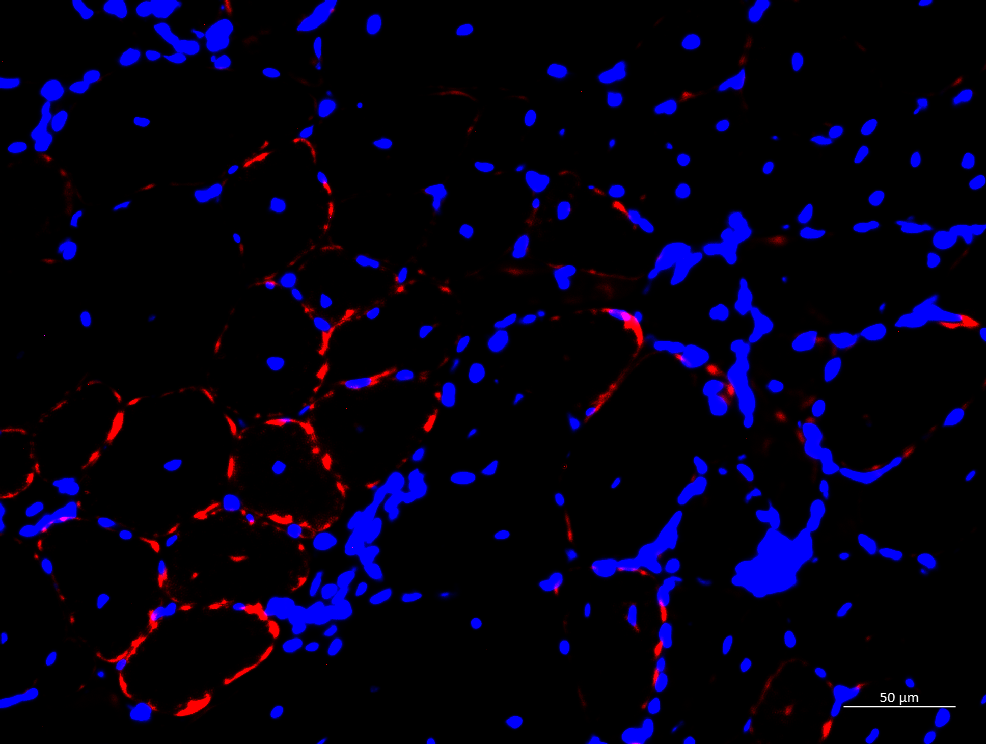

Supplement: Supplementary file 10 — Source data Fig. 8 [file 44321_2025_273_MOESM10_ESM.zip › Fig.8/Fig8-G/Control-AmNA-Merge.tif]

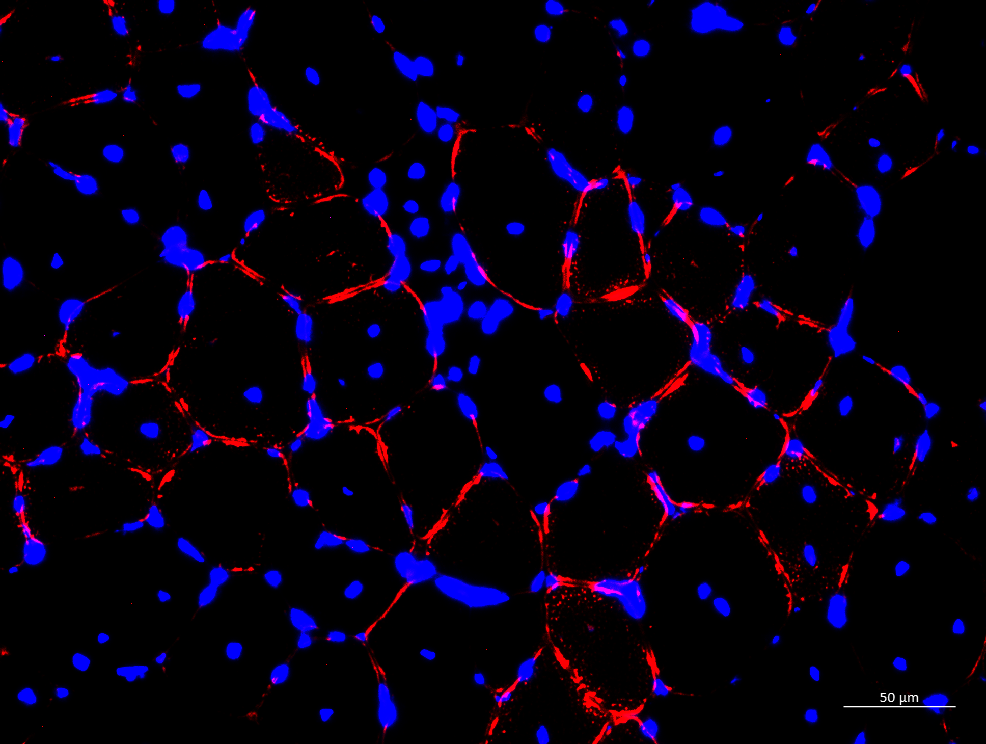

Supplement: Supplementary file 10 — Source data Fig. 8 [file 44321_2025_273_MOESM10_ESM.zip › Fig.8/Fig8-G/Anti-miR-33b-Merge.tif]

Fig8-A

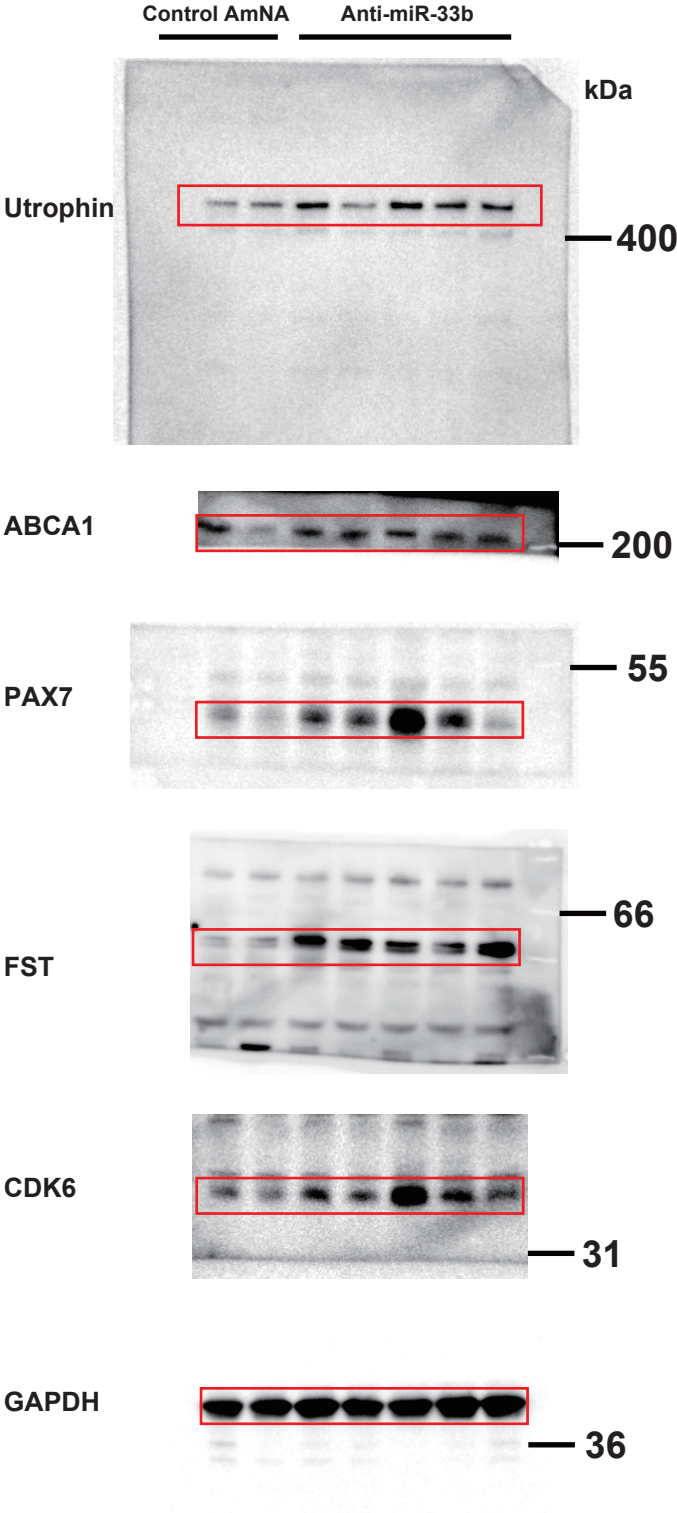

Supplement: Supplementary file 10 — Source data Fig. 8 [file 44321_2025_273_MOESM10_ESM.zip › Fig.8/Fig8-A/Fig8-A.pdf]

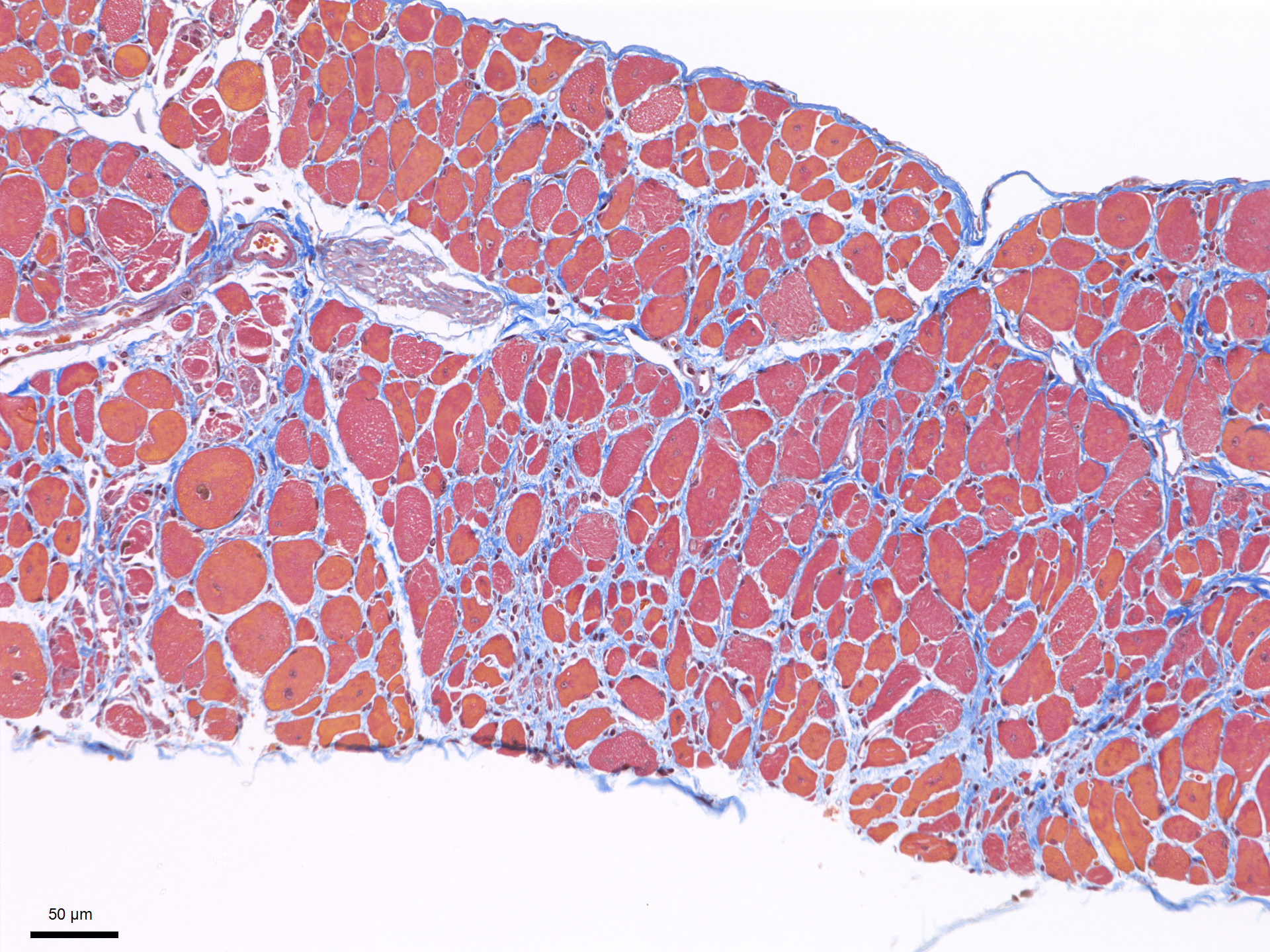

Supplement: Supplementary file 10 — Source data Fig. 8 [file 44321_2025_273_MOESM10_ESM.zip › Fig.8/Fig8-D/Control AmNA-Diaphragm.tif]

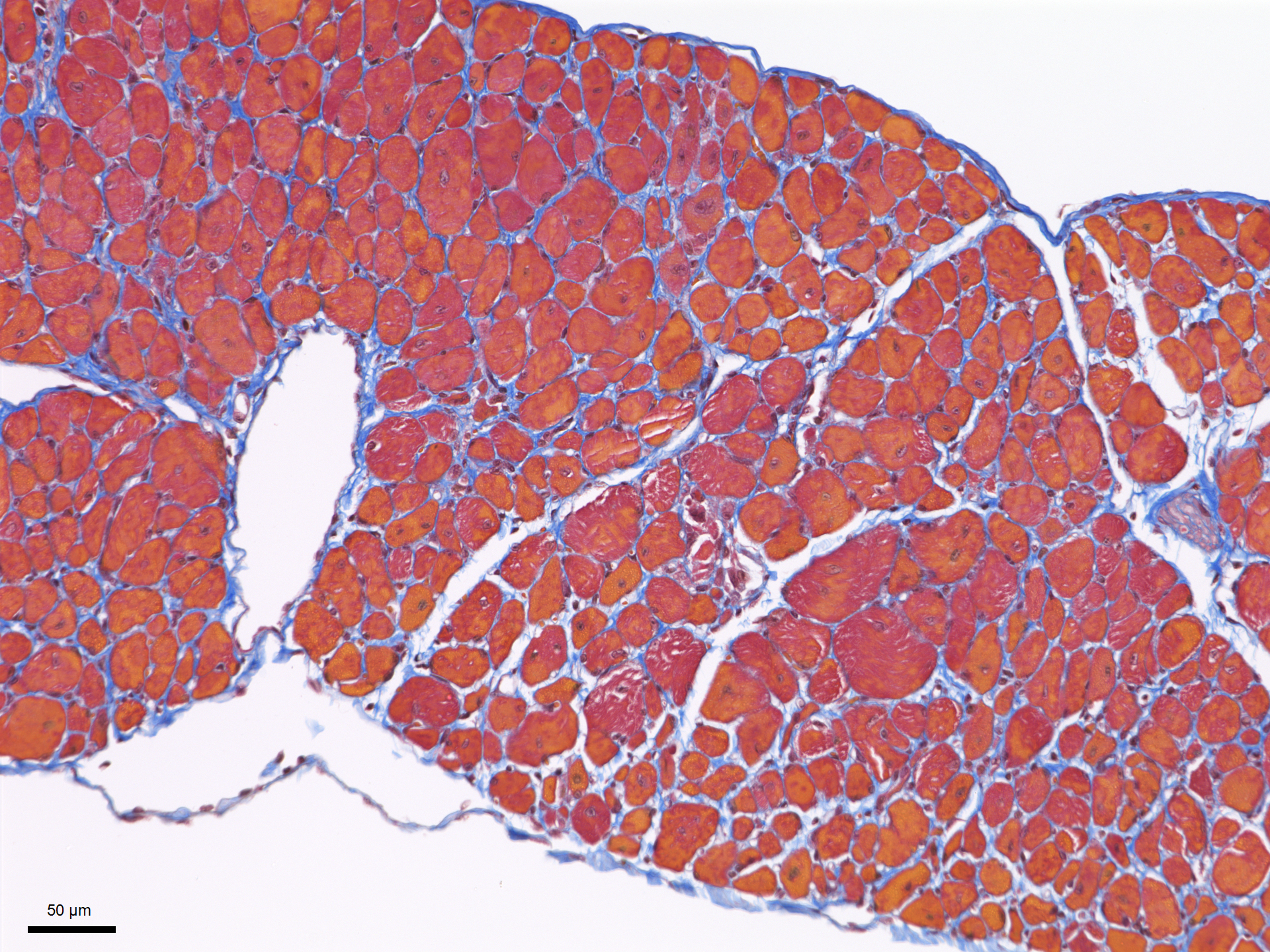

Supplement: Supplementary file 10 — Source data Fig. 8 [file 44321_2025_273_MOESM10_ESM.zip › Fig.8/Fig8-D/Anti-miR-33b-Diaphragm.tif]

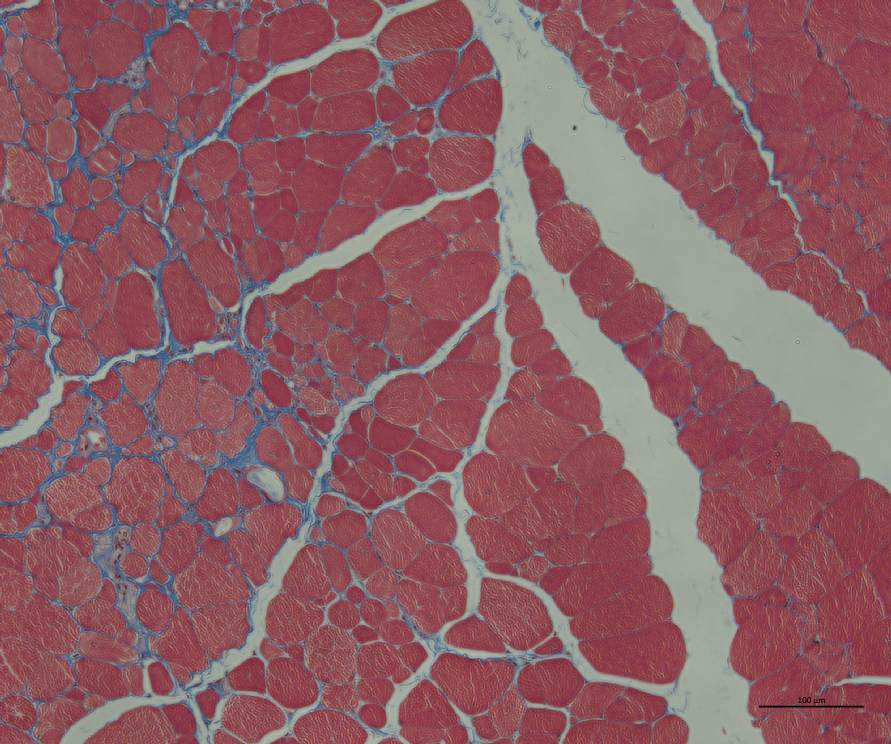

Supplement: Supplementary file 10 — Source data Fig. 8 [file 44321_2025_273_MOESM10_ESM.zip › Fig.8/Fig8-D/Control AmNA-GAS.tif]

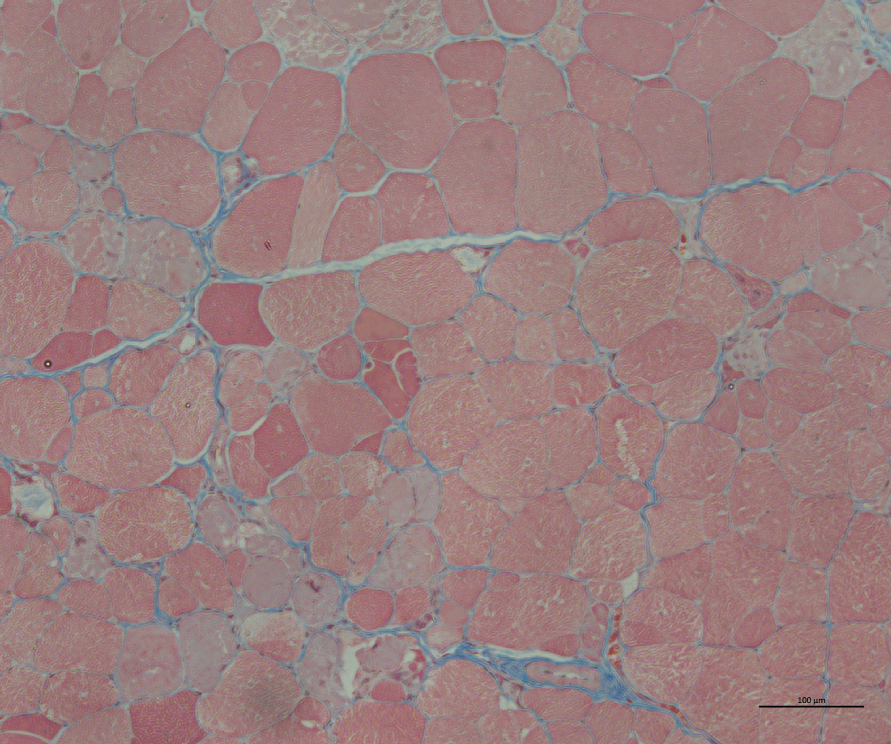

Supplement: Supplementary file 10 — Source data Fig. 8 [file 44321_2025_273_MOESM10_ESM.zip › Fig.8/Fig8-D/Control AmNA-TA.tif]

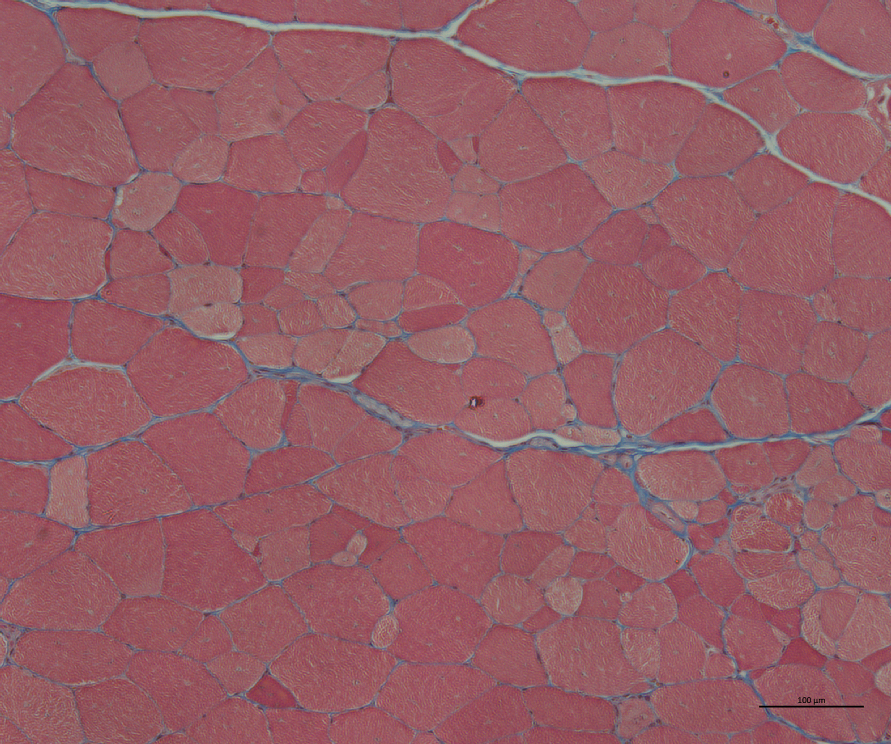

Supplement: Supplementary file 10 — Source data Fig. 8 [file 44321_2025_273_MOESM10_ESM.zip › Fig.8/Fig8-D/Anti-miR-33b-TA.tif]

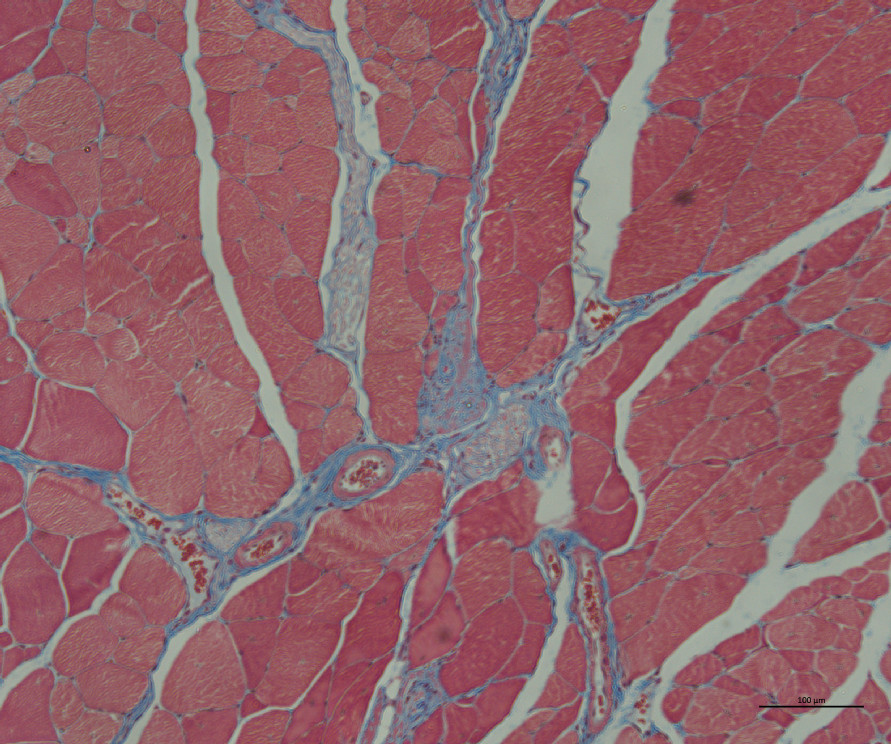

Supplement: Supplementary file 10 — Source data Fig. 8 [file 44321_2025_273_MOESM10_ESM.zip › Fig.8/Fig8-D/Anti-miR-33b-GAS.tif]

Fig9-F

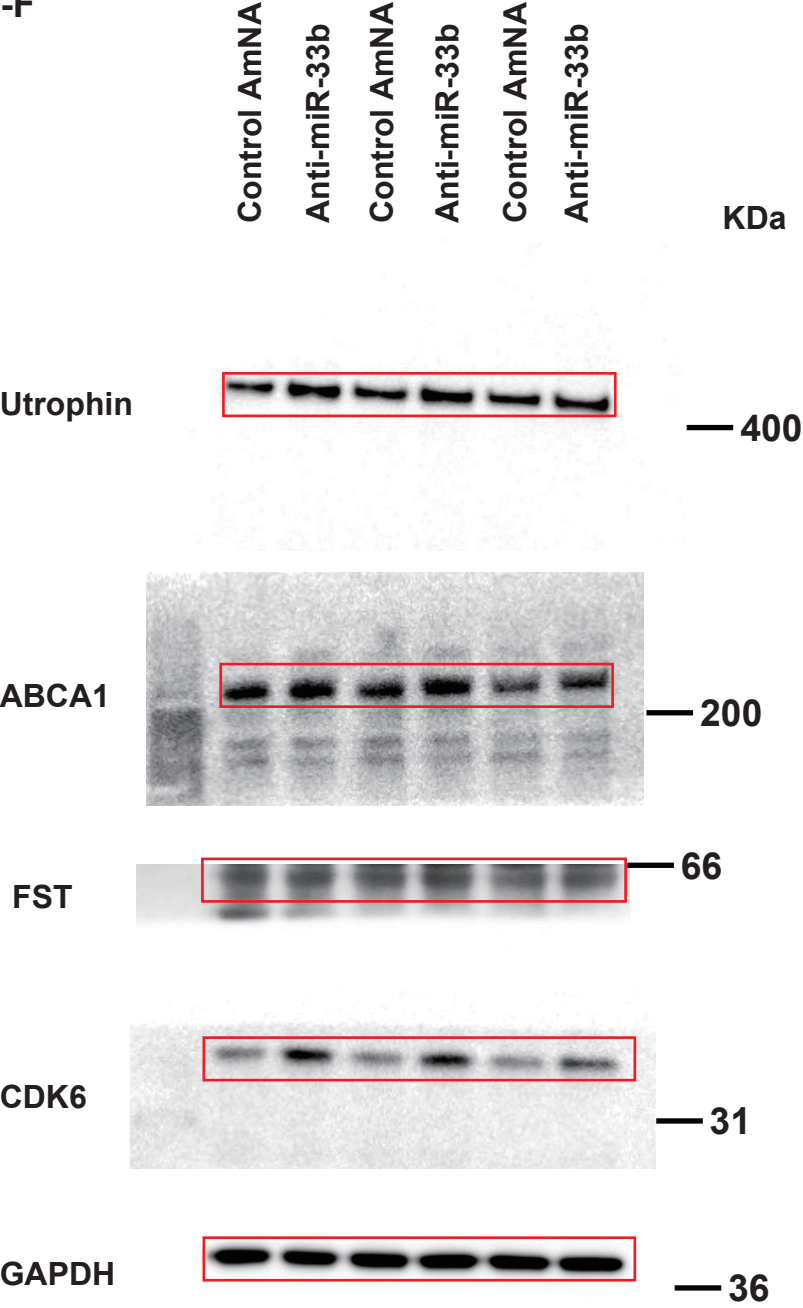

Supplement: Supplementary file 11 — Source data Fig. 9 [file 44321_2025_273_MOESM11_ESM.zip › Fig.9/Fig9-F/Fig9-F.pdf]
